# Aberrant Autolysosomal Regulation Is Linked to The Induction of Embryonic Senescence: Differential Roles of Beclin 1 and p53 in Vertebrate Spns1 Deficiency

**DOI:** 10.1371/journal.pgen.1004409

**Published:** 2014-06-26

**Authors:** Tomoyuki Sasaki, Shanshan Lian, Jie Qi, Peter E. Bayliss, Christopher E. Carr, Jennifer L. Johnson, Sujay Guha, Patrick Kobler, Sergio D. Catz, Matthew Gill, Kailiang Jia, Daniel J. Klionsky, Shuji Kishi

**Affiliations:** 1 Department of Metabolism & Aging, The Scripps Research Institute, Jupiter, Florida, United States of America; 2 Campbell Family Cancer Research Institute, Ontario Cancer Institute, Princess Margaret Cancer Centre, University Health Network, Toronto, Ontario, Canada; 3 Department of Earth, Atmospheric and Planetary Sciences, Massachusetts Institute of Technology, Cambridge, Massachusetts, United States of America; 4 Department of Molecular and Experimental Medicine, The Scripps Research Institute, La Jolla, California, United States of America; 5 Department of Biological Sciences, Florida Atlantic University, Jupiter, Florida, United States of America; 6 Life Sciences Institute, Department of Molecular, Cellular, and Developmental Biology, Department of Biological Chemistry, University of Michigan, Ann Arbor, Michigan, United States of America; University of Pennsylvania School of Medicine, United States of America

## Abstract

Spinster (Spin) in *Drosophila* or Spinster homolog 1 (Spns1) in vertebrates is a putative lysosomal H^+^-carbohydrate transporter, which functions at a late stage of autophagy. The Spin/Spns1 defect induces aberrant autolysosome formation that leads to embryonic senescence and accelerated aging symptoms, but little is known about the mechanisms leading to the pathogenesis *in vivo*. Beclin 1 and p53 are two pivotal tumor suppressors that are critically involved in the autophagic process and its regulation. Using zebrafish as a genetic model, we show that Beclin 1 suppression ameliorates Spns1 loss-mediated senescence as well as autophagic impairment, whereas unexpectedly p53 deficit exacerbates both of these characteristics. We demonstrate that ‘basal p53’ activity plays a certain protective role(s) against the Spns1 defect-induced senescence via suppressing autophagy, lysosomal biogenesis, and subsequent autolysosomal formation and maturation, and that p53 loss can counteract the effect of Beclin 1 suppression to rescue the Spns1 defect. By contrast, in response to DNA damage, ‘activated p53’ showed an apparent enhancement of the Spns1-deficient phenotype, by inducing both autophagy and apoptosis. Moreover, we found that a chemical and genetic blockage of lysosomal acidification and biogenesis mediated by the vacuolar-type H^+^-ATPase, as well as of subsequent autophagosome-lysosome fusion, prevents the appearance of the hallmarks caused by the Spns1 deficiency, irrespective of the basal p53 state. Thus, these results provide evidence that Spns1 operates during autophagy and senescence differentially with Beclin 1 and p53.

## Author Summary

Spinster homolog 1 (Spns1) in vertebrates, as well as Spinster (Spin) in *Drosophila*, is a hypothetical lysosomal H^+^-carbohydrate transporter, which functions at a late stage of autophagy. The Spin/Spns1 defect induces aberrant autolysosome formation that leads to embryonic senescence and accelerated aging symptoms, while the molecular mechanisms of the pathogenesis are unknown *in vivo*. Using zebrafish, we show that Beclin 1 suppression ameliorates Spns1 loss-mediated senescence as well as autolysosomal impairment, whereas p53 deficit unexpectedly exacerbates these characteristics. We demonstrate that basal p53 activity has a certain protective role(s) against the Spns1 defect via suppressing autophagosome-lysosome fusion, while p53 activated by ultraviolet radiation amplifies the Spns1 deficit. In addition, we found that excessive lysosomal biogenesis and prolonged suboptimal acidification, modulated by v-ATPase, could be the primary reason for the appearance on the hallmarks of Spns1 deficiency. Our findings thus suggest that Spns1 is critically involved in lysosomal acidification and trafficking during autophagy, and differentially acts in a pathway with Beclin 1 and p53 in the regulation of senescence.

## Introduction

Autophagy is an evolutionarily conserved intracellular catabolic process whereby cytoplasmic proteins and organelles are engulfed into autophagosomes and subsequently degraded in autolysosomes, following fusion with lysosomes. Biologically significant roles of autophagy have been illuminated in a variety of physiological and pathophysiological conditions, such as occurs during the adaptation to nutrient starvation, the clearance of damaged proteins and cell organelles, development, cell survival and death, tumor progression and suppression, elimination of pathogens, and aging [1]. It has also been suggested that autophagy can have a beneficial effect on longevity in many lower organisms from yeast to flies, although a clear role in lifespan extension still remains elusive in vertebrates [2]. Furthermore, several interventions that promote longevity, including caloric restriction and chemical treatment with rapamycin, have exploited their impact through autophagy [3].

Zebrafish is an ideal organism to study the entire developmental process *ex utero* and are easily accessible for both experimental and genetic manipulations. Therefore, the zebrafish model system has become a popular platform to explore the mechanisms of human diseases [4]. Recently in our laboratory, we screened mutagenized zebrafish embryos for the altered expression of senescence-associated β-galactosidase (SA-β-gal), which is a versatile senescence biomarker widely used in both cellular senescence and organismal aging studies [5], [6], [7]. SA-β-gal has also been utilized for various detection of embryonic/larval senescence in our studies and those of others [8], [9], [10], [11]. We successfully validated the use of embryonic SA-β-gal production as a valuable screening tool by analyzing over 500 zebrafish mutants [12]. Of our identified mutants, the highest SA-β-gal activity was found to be associated with an insertion in the gene denoted “*not really started*” (*nrs*) (currently denoted as zebrafish *spinster homolog 1*, *spns1*), which is a homolog of *Drosophila spinster*, a gene that regulates aging and lifespan in flies [13]. Zebrafish harboring a homozygous mutation in the *spns1* gene revealed embryonic/larval lethality, associated with yolk opaqueness and senescence [12], [14]. Adult zebrafish with a heterozygous deletion of *spns1* show accelerated signs of aging, including an increased accumulation of the “aging pigment” lipofuscin in the muscle and liver, and have shortened lifespan [12]. Spinster has been implicated in a lysosomal storage function in flies [13], [15], and Spns1 deficiency leads to impaired autophagic termination and lysosome reformation problems in the mammalian cell culture system [16]. However, it remains unknown how Spns1 physiologically and pathophysiologically has an impact on autophagic homeostasis in conjunction with senescence in higher organisms *in vivo*, where we lack an appropriate vertebrate model system except for zebrafish.

Beclin 1, an autophagic regulator, is essential for early embryonic development, and is a haploinsufficient tumor suppressor [17]. During starvation of cultured cells, the accumulation of large and long-lasting autolysosomes caused by Spns1 deficiency is attenuated by concurrent *beclin 1* knockdown, suggesting dependence on autophagy induction and progression [16]. p53, the most extensively characterized tumor suppressor, is a master regulator with pleiotropic effects on genomic stability, cell cycle, proliferation, cell death, tumorigenesis, stress response, senescence and energy metabolism, and is also involved in autophagic regulation [18]. p53 had been exclusively considered as a positive regulator of autophagy [19], but was recently found also to act as an autophagic inhibitor [20], [21]. Thus, the role of p53 in autophagy regulation requires further study since it may underlie key aspects of metabolism, aging, and cancer biology.

We examined the impact of Spns1 impairment on the autophagic process and on the induction of embryonic senescence in zebrafish, in order to clarify how autolysosomal processing is linked to these two tumor suppressors, Beclin 1 and p53. In this study, we found that inhibition of Beclin 1 can attenuate the yolk opacity and senescence caused by the Spns1 defect, whereas deficiency of “basal” p53 augments them (“basal” meaning in the absence of extrinsic genotoxic stress, e.g., ultraviolet light). Conversely, p53 “activated” by DNA damage apparently induced autophagy and apoptosis, intensifying the Spns1-deficient phenotype. Moreover, a chemical and genetic blockage of lysosomal acidification by inhibition of vacuolar-type H^+^-ATPase (v-ATPase) prevented the appearance of the hallmarks of Spns1 deficiency irrespective of the p53 state, while at the same time preventing autophagosome-lysosome fusion. Our findings thus suggest that Spns1 is critically involved in lysosomal acidification and trafficking during autophagy, and acts in the same pathway as Beclin 1 and p53 in the regulation of senescence.

## Results

### Accumulation of cytoplasmic membranous inclusions and LC3 puncta in *spns1*-mutant fish

**Figure 1:**
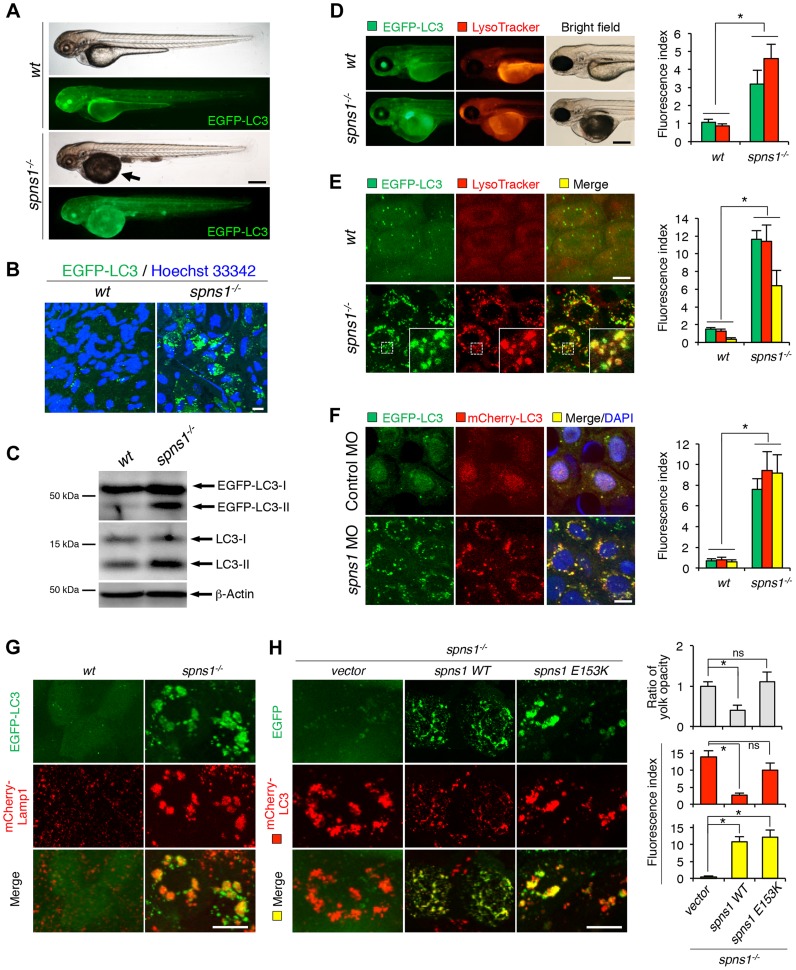
Aberrant autophagosome and autolysosome formation in *spns1*-mutant zebrafish. (**A**) Yolk opaqueness and LC3 puncta formation in *spns1*-mutant zebrafish embryos. For EGFP-LC3 transgenic *spns1*-mutant [*Tg(CMV:EGFP-LC3);spns1^hi891/hi891^*] fish siblings, bright-field and fluorescence images of wild-type (*wt*) control (upper) and *spns1* mutant (*spns1^−/−^*) (lower) embryos at 84 hpf are shown. The black arrow indicates the yolk-opaqueness phenotype in the *spns1* mutant. The gross expression of EGFP-LC3 at head and trunk in the *spns1*-mutant animal is relatively stronger than in the *wt* animal. Occasionally, however, a high intensity signal can be observed at the liver region in the mutant (as seen in **D**). Scale bar, 250 µm. (**B**) EGFP-LC3 punctate compartments in the liver cells of the *spns1* mutant. Through high magnification (×600) confocal microscopy, intracellular EGFP-LC3 puncta were visualized in live animals at 84 hpf. Nuclei were counterstained with Hoechest 33342 (blue), and peri-nuclear EGFP-LC3 puncta were evident in the *spns1* mutant, but not in *wt* animals. Scale bar, 10 µm. (**C**) Immunoblotting to detect the conversion of LC3-I to LC-II. Using an anti-LC3 antibody, both endogenous LC3 and transgenic (exogenous) EGFP-LC3 expression was detected and an increase of LC3-II conversion/accumulation was seen in the *spns1* mutant compared with *wt* fish at 84 hpf. (**D**–**F**) Identification of autophagosome and autolysosome/lysosome formation in the *spns1* mutant. (D, E) LysoTracker (DND-99; red) staining of EGFP-LC3 transgenic *spns1*-mutant [*Tg(CMV:EGFP-LC3); spns1^hi891/hi891^*] embryos was performed at 84 hpf. At the whole animal levels (D), the EGFP-LC3 signal is relatively higher throughout in the *spns1* mutant than in wild type, and a particularly strong signal can be seen in the liver, as shown in (A). In the head and trunk portions of the animals (D), a distinctive increase in the intensity of LysoTracker can be observed in the *spns1* mutant. At the intracellular level (E), several small LC3 spots and largely diffuse green signal in the cells and cytosolic LysoTracker staining is seen. A number of enlarged LC3- and LysoTracker-positive yellow punctate structures can be seen in the *spns1* mutant by confocal microscopy at a higher magnification (inset; enlarged from dotted square area). (F) EGFP-LC3 and mCherry-LC3 double-transgenic [*Tg(EGFP-LC3:mCherry-LC3)*] zebrafish were used to monitor autolysosome formation in *spns1* MO-injected embryos at 84 hpf. A number of enlarged yellow LC3 puncta were detected in the *spns1* morphant, while only small yellow LC3 spots can be seen in control-injected embryos. Nuclei were counterstained with 4′, 6-diamidino-2-phenylindole, dihydrochloride (DAPI). Scale bar, 250 µm in (D). Scale bar, 10 µm in (E, F). Quantification of data presented in D (n = 12), E (n = 6), and F (n = 6) is shown in the right graph; the number (n) of animals is for each genotype. Three independent areas (periderm or basal epidermal cells above the eye) were selected from individual animals. (**G**) Transgenic expression of mCherry-Lamp1 in *wt* [*Tg(CMV:EGFP-LC3)*] and *spns1*-mutant [*Tg(CMV:EGFP-LC3);spns1^hi891/hi891^*] animals 84 hpf. Scale bar, 10 µm. (**H**) Transgenic expression of EGFP-Vector (*vector*), EGFP-wild-type Spns1 (*spns1 WT*), or EGFP-mutant Spns1 (*spns1 E153K*) in [*Tg(CMV:mCherry-LC3);spns1^hi891/hi891^*] animals at 84 hpf. Scale bar, 10 µm. Quantification of data presented in H is shown for ratio of yolk opaqueness phenotype (n = 48), mCherry intensity (red) (n = 6), and merge intensity of EGFP and mCherry (yellow) (n = 6) in the right graphs; the number (n) of animals is for each genotype. Three independent areas (periderm or basal epidermal cells above the eye) were selected from individual animals. Error bars represent the mean ± standard deviation (S.D.), *p<0.005; ns, not significant.

Spin/Spns1 has been implicated in the regulation of autophagic lysosomal homeostasis in mammalian cells and flies [15], [16]. In fact, in zebrafish, electron microscopy revealed that compared with the wild-type control, *spns1*-mutant larvae accumulated cytoplasmic membranous inclusions corresponding to late endosomal, autophagic, and lysosomal structures in the hypodermal and retinal epithelial cells (**Figure S1A**). To verify that the autophagic process of *spns1-*deficient (*spns1^hi891/hi891^*) vertebrates is fundamentally disturbed, we generated EGFP-tagged microtubule-associated protein 1 light chain 3 (LC3) transgenic zebrafish with the *spns1*-mutant background. In the resulting EGFP-LC3-transgenic *spns1*-mutant [*Tg(CMV:EGFP-LC3); spns1^hi891/hi891^*] fish line, grossly enhanced EGFP intensity was observed throughout the body in comparison with the original *Tg(CMV:EGFP-LC3)* line [22], [23] (**Figure 1A**). In addition, intracellular localization of EGFP-LC3 was detectable as aggregated puncta in periderm or basal epidermal cells of the skin (above the eye on the head or in the caudal fin) and epithelial cells of several other organs including yolk sac, retina, and liver (**Figure 1B**), suggesting excessive autophagosome and/or autolysosome accumulation.

To gain additional information concerning the site of action of Spns1, we examined LC3 conversion as a hallmark of autophagy induction in whole zebrafish embryos by immunoblotting to distinguish the autophagosome-associated phosphatidylethanolamine-conjugated LC3-II from the cytosolic LC3-I form by showing the increased mobility of LC3-II. In *spns1* mutants, both endogenous LC3-II and exogenous EGFP-LC3-II were detected at higher levels (**Figure 1C**).

Extending our analysis to a second animal model, we also examined autophagy activity in *Caenorhabditis elegans* containing a loss-of function mutation in the gene homologous to *spin-1* (C13C4.5) [24]. Similar to our results in zebrafish, the *C. elegans spin-1* mutation conferred augmented autophagic induction, as demonstrated by the increased expression and cytoplasmic aggregation of the *EGFP::LGG-1* reporter gene product (LGG-1 is the ortholog of LC3) in seam cells of mutant animals (**Figure S1B and C**). We found the *spin-1* mutant worms were more sensitive to starvation-induced death (**Figure S1D**), consistent with defective autophagy. In addition, decrease of Spns1 in heterozygous zebrafish as well as loss of Spin-1 in homozygous worms resulted in significant reductions in their adult lifespan (**Figure S1E and F**). These data suggest that across these different species, the defects in the *spns1/spin-1* gene induce autophagic abnormality with excessive autophagosomes and/or autolysosomes, potentially leading to the accumulation of undegraded macromolecules and organelles in cells of mutant animals, which subsequently have a shortened life expectancy.

### Lysosomal, but not mitochondrial, abnormalities in the pathogenesis of *spns1* mutants

Spin/Spns1 is a multi-pass transmembrane protein localized in late endosomes and lysosomes [15], [25]. In mammalian cells, however, Spns1 has been reported to occasionally localize to mitochondria [26]. To elucidate a potential relationship between lysosomal and mitochondrial biogenesis with the pathogenesis induced by the Spns1-defective animals *in vivo*, we performed double staining of these two organelles by using LysoTracker (red) and MitoTracker (green) probes. In whole animal images, we found prominent increases of LysoTracker intensity in *spns1*-mutant fish, whereas no significant difference was detected by MitoTracker staining (**Figure S2A**). By further utilizing *Tg(CMV:EGFP-LC3);spns1^hi891/hi891^* animals, concurrent LysoTracker staining revealed significant numbers of intracellular yellow (both green- and red-positive) puncta. Since the EGFP green signal is normally lost by quenching in acidic compartments such as the lysosome [27], this finding suggests the existence of insufficiently acidic autolysosomes (**Figure 1D and E**). In contrast, staining with a mitochondrial superoxide indicator, MitoSOX, revealed no critical abnormality of superoxide generated in the mitochondria (**Figure S2B**). These results suggest that Spns1 deficiency fundamentally leads to impaired lysosomal and/or autolysosomal acidification, but not to any significant modulation of mitochondrial biogenesis and oxidative stress.

### Formation of enlarged mal-acidic cellular deposits caused by the Spns1 defect

Autophagosomes subsequently fuse with lysosomes to degrade their contents. The Spns1 defect causes excessively enlarged undegraded deposits of autolysosomal compartments in cells [16]. The inability of *spns1* mutants to degrade protein aggregates, despite the apparent induction of autophagosomes, prompted us to ask whether Spns1 is required for degradation of autophagic cargos by ensuring proper acidification in autolysosomes. To address this question, we generated *EGFP-LC3;mCherry-LC3* double-transgenic zebrafish [*Tg(CMV:EGFP-LC3;mCherry-LC3); spns1^hi891/hi891^*] to determine the acidification efficiency. As EGFP fluorescence is lost in acidic compartments, but mCherry red fluorescence is not, the coexpression of EGFP-LC3 and mCherry-LC3 can label insufficiently acidified autolysosomes as well as non-acidic autophagosomes to produce yellow fluorescence (positive for both green EGFP and red mCherry), whereas acidic autolysosomes would only show a red fluorescent signal.

To first validate that the EGFP signal was decreased or lost by quenching in acidic autolysosomes of wild-type animals, we utilized two lysosomal protease inhibitors, pepstatin A, an inhibitor of cathepsins D and E, and E-64-d, an inhibitor of cathepsins B, H and L. Because these inhibitors can target the proteases without altering autolysosomal acidity, we anticipated that the EGFP signal would only be reduced in truly acidic vesicles. In wild-type animals, as expected, only the large punctate signals of EGFP-LC3 were faded, whereas neither the LysoTracker nor mCherry-LC3 signals were affected (**Figure S2C and D**). On the other hand, as shown in **Figure 1F**, once *spns1* morpholino antisense oligonucleotide (MO) was injected into the GFP- and mCherry-LC3-double transgenic fish embryos to knockdown the gene expression, we observed a prominent increase in the number of yellow-fluorescent enlarged intracellular vesicles as compared with those in standard control MO-injected animals, consistent with the accumulation of insufficiently acidified autolysosomes. The EGFP-LC3-positive vesicles in the *spns1* mutants were further confirmed to be autolysosomes by the co-expression of a mCherry-tagged lysosomal membrane marker, lysosomal-associated membrane protein 1 (Lamp1) (**Figure 1G**). mCherry-LC3-positive enlarged vesicular aggregations that accumulated in the *spns1*-mutant fish were suppressed by expression of EGFP-tagged Spns1 vector (Spns1 WT) but not by that of an empty EGFP vector or an EGFP-tagged mutant Spns1 vector (Spns1 E153K; presumably disrupted for the transporter activity) [15], [16] (**Figure 1H**).

In addition, the vast majority of EGFP-LC3-positive vesicles in *spns1* mutants were found to be still positive for a fluorogenic lysosomal substrate DQ Red BSA at the earlier phenotypic stages (∼60 hours post fertilization; hpf) (**Figure S2E**). DQ Red BSA fluoresces upon lysosomal degradation due to dequenching; the released peptide fragments are brightly fluorescent. Thus, the autolysosomes of *spns1*-mutant fish appeared to still contain hydrolytic activity at least in early autolysosomes, indicating that the primary reason for the retained EGFP-LC3 signal is probably due to suboptimal acidity at later stages. Therefore, the observed increase in both EGFP-LC3 and mCherry-LC3 double-positive yellow fluorescent intracellular vesicles in *spns1*-mutant fish could be attributed to ineffective or insufficient acidification (“mal-acidification”) at the late autolysosomal stage.

### Rescue of the Spns1 deficit in zebrafish by suppression of Beclin 1

**Figure 2:**
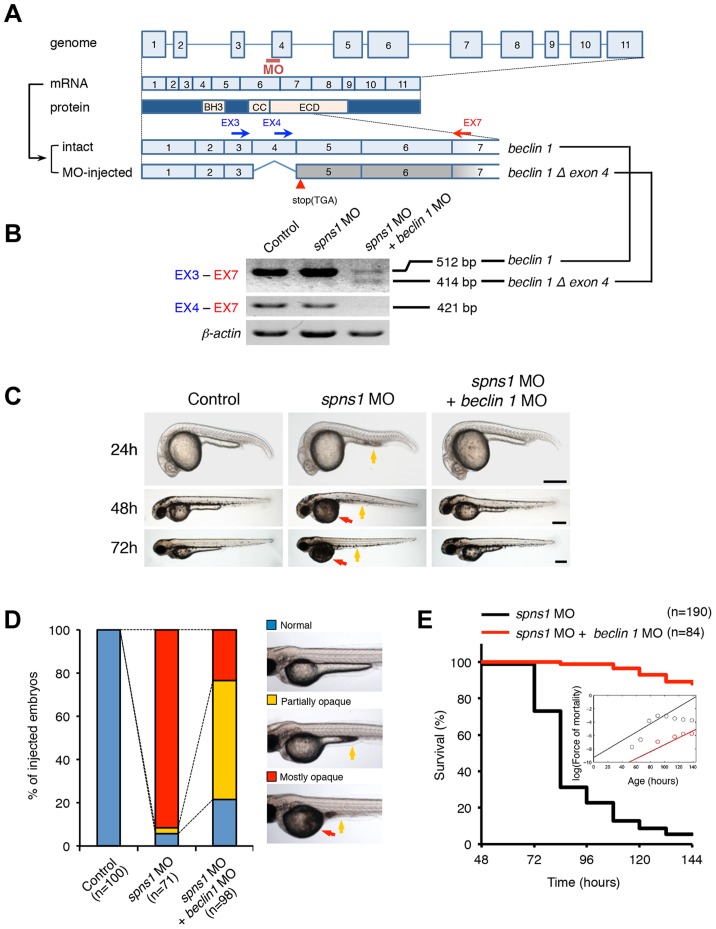
Knockdown of *beclin 1* suppresses the Spns1 deficiency in zebrafish. (**A**) Schematic representation of the zebrafish *beclin 1* (*zbeclin 1*) gene, its mRNA and protein products. A splice-blocking *beclin 1* MO was designed to overlap the intron-exon boundary at the 5′-splice junction of exon 4 in the zebrafish *beclin 1* gene. To detect aberrantly spliced RNA products, two forward primers were designed for exon 3 (EX3 primer) and exon 4 (EX4 primer), and one reverse primer was designed for exon 7 (EX7 primer) within the *beclin 1* gene. The zebrafish *beclin 1* gene has a total of 11 exons having three unique domains [BH3 domain, coiled-coil (CCD) domain, and evolutionarily conserved (ECD) domain], and the *beclin 1* MO was anticipated to disrupt the BH3 domain encoded by exon 4 and exon 5. (**B**) Splicing detection of *zbeclin 1* mRNA by RT-PCR. Amplified PCR fragments show the intact sizes of the two amplicons for EX3-EX7 and EX4-EX7 following control (water) injection or only *spns1* MO injection. Either *beclin 1* MO (12 ng/embryo) injection or coinjection of *spns1* MO (4 ng/embryo) and *beclin 1* MO (12 ng/embryo) generated a skipping of exon 4 (*beclin 1Δexon4*). This was detected by the presence of an altered EX3-EX7 amplicon and undetectable EX4-EX7 product. The deletion of exon 4 was confirmed by sequencing. Injected embryos were harvested for total RNA isolation at 54 hpf. (**C and D**) Rescue of the *spns1* morphant by *beclin 1* knockdown. (C) The yolk opaqueness phenotype appearance in control-injected (water), *spns1* MO-injected, and *spns1* and *beclin 1* MOs-coinjected embryos was followed through 72 hpf. At 24 hpf, opaqueness commenced from the yolk extension region, which had almost disappeared or was severely damaged (more than 95% of *spns1* MO-injected animals) with an extension of opacity to the entire yolk at 48 hpf. By 72 hpf, yolk opaqueness became highly dense throughout most of the *spns1* MO-injected embryos, which usually died within another 24 h. Scale bar, 250 µm. (D) Clarification of the yolk opaqueness phenotype in *spns1* morphants at 72 hpf. As described in (C), more than 95% of the *spns1* MO-injected embryos showed a ‘mostly opaque’ yolk at 48 hpf, and such embryos subsequently died. Animals showing a ‘partially opaque’ yolk could sometimes be recovered and subsequently survived 96 h and beyond. *beclin 1* MO coinjection dramatically increased (more than 10 times) the animal numbers with the partial yolk opaque phenotype. (**E**) Survival curve for *spns1* morphant and *spns1;beclin 1*-double morphant larvae (log rank test: χ^2^ = 162.5 on one degree of freedom; *p*<0.0001).

Based on a recent report of an autophagy-dependent effect of *spns1* knockdown in a mammalian cell culture [16] and our current observations described above in the zebrafish model, we assumed that inhibition of the early stages of autophagy by blocking the class III phosphatidylinositol 3-kinase (PtdIns3K) complex containing Vps34/Pik3c3 and Beclin 1 would reduce aggregated LC3 puncta in cells of *spns1* mutants and ameliorate yolk opaque abnormalities induced by the Spns1 deficiency. We therefore designed a splice-block morpholino antisense oligonucleotide (MO) targeting the zebrafish *beclin 1* (*becn1*; *zbeclin 1*) gene at the 5′ end of exon 4 (**Figure 2A**). RT-PCR and DNA sequencing results showed this splice-block MO (*beclin 1* MO) generated a loss of exon 4 and a premature stop codon, resulting in a truncated protein lacking the entire Bcl2 homology domain 3 (BH3 domain) (**Figure 2B**). The phenotype induced by the knockdown of *beclin 1* by the MO during early development was not particularly evident at the gross morphology level apart from some minor developmental retardation at 24 hpf, without any obvious abnormality later on (**Figure S3A**). In contrast, the concurrent suppression of both *spns1* and *beclin 1* by MO targeting strikingly diminished the yolk opaqueness seen with the *spns1* morphants and produced an increased number of viable larvae that survive beyond 72 hpf (**Figure 2C–E**). We also performed *beclin 1* MO injections into *spns1*-mutant embryos, and reproducibly confirmed the ameliorated yolk phenotype through 3 dpf (**Figure S3B**), but mutant animals subsequently relapsed into deterioration, presumably due to the persistent impact of the Spns1 mutation and/or transient activity of the *beclin 1* MO. These results indicate that suppression of the early stage of autophagy by *beclin 1* knockdown can offset the deleterious effect of Spns1 deficiency that occurs at the late stage of autophagy.

**Figure 3:**
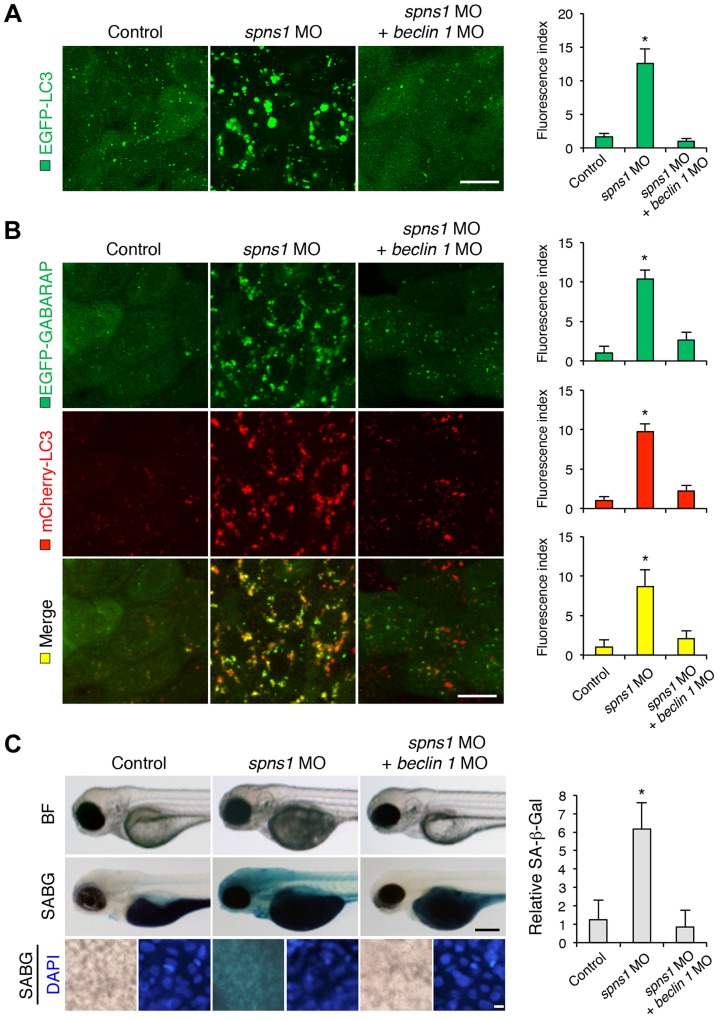
Knockdown of *beclin 1* suppresses abnormal autolysosomal puncta formation and embryonic senescence caused by Spns1 deficiency in zebrafish. (**A**) Effect of *beclin 1* knockdown on EGFP-LC3 puncta formation in *spns1*-depleted zebrafish embryos. Injection of control (water) injection, *spns1* MO (4 ng/embryo) or coinjection of *spns1* MO (4 ng/embryo) and *beclin 1* MO (12 ng/embryo) into *Tg(CMV:EGFP-LC3)* fish was performed to assess whether the *beclin 1* knockdown reduces or eliminates aggregated LC3 puncta induced by Spns1 depletion at 84 hpf. Scale bar, 10 µm. Quantification of data presented in panel A (n = 12) is shown in the right graph; the number (n) of animals is for each morphant or water-injected control. Three independent areas (periderm or basal epidermal cells above the eye) were selected from individual animals. (**B**) Effect of *beclin 1* knockdown on EGFP-GABARAP as well as mCherry-LC3 puncta formation in *spns1*-depleted zebrafish embryos. Injection of control (water), *spns1* MO or coinjection of *spns1* MO and *beclin 1* MO into *Tg(CMV:EGFP-GABARAP;mCherry-LC3)* fish was performed to evaluate whether the *beclin 1* knockdown reduces or eliminates the aggregation of GFP-GABARAP puncta in comparison with those of LC3 caused by the Spns1 depletion at 84 hpf. Scale bar, 10 µm. Quantification of data presented in the top row (green; EGFP) (n = 9), middle row (red; mCherry) (n = 12), and bottom row (yellow; merge of EGFP and mCherry) (n = 9) in panel B is shown in the right graphs; the number (n) of animals is for each morphant or water-injected control. Three independent areas (periderm or basal epidermal cells above the eye) were selected from individual animals. (**C**) Effect of *beclin 1* knockdown on embryonic senescence in *spns1* morphant. By using the same injection samples [injection of control (water), *spns1* MO or coinjection of *spns1* MO and *beclin 1* MO into *Tg(CMV:EGFP-GABARAP;mCherry-LC3)* fish], SA-β-gal staining was performed to assess whether the *beclin 1* knockdown has any impact on the embryonic senescence caused by Spns1 depletion at 84 hpf. Representative images of individual fish by bright field (BF, live samples) and SA-β-gal (SABG) staining are shown in the upper and middle panels, respectively. Scale bar, 250 µm. Lower panels are larger magnification images of corresponding SA-β-gal samples shown in the middle panels and the fluorescence images of nuclei counterstained with DAPI. Scale bar, 10 µm. Quantification of data presented in the middle row (SABG) in panel C (n = 12) is shown in the right graph; the number (n) of animals is for each morphant or water-injected control. Error bars represent the mean ± S.D., *p<0.005.

We next examined whether the enlarged aggregations of LC3 in *spns1* morphants and mutants can be restored by Beclin 1 knockdown. *spns1* MO and/or *beclin 1* MO were introduced into *Tg(CMV:EGFP-LC3)* fish embryos and resultant specimens were observed by confocal microscopy at the cellular level. The appearance of punctate vesicle-like intracellular aggregates and deposits observed in *spns1* morphants was diminished by the *beclin 1* knockdown (**Figure 3A**). LC3 has several functional homologs, including gamma-aminobutyric acid A (GABA)-receptor associated protein (GABARAP) and GABARAPL2/GATE-16. It has been reported that both LC3 and GABARAP are indispensable for the autophagic process in mammalian cells [21]. The restorative effect of *beclin 1* knockdown was also demonstrated in *spns1*-depleted *Tg(CMV:EGFP-GABARAP;mCherry-LC3)* fish. The concomitant microinjection of *spns1* MO and *beclin 1* MO showed consistently similar outcomes in terms of the obvious reduction of both EGFP-GABARAP and mCherry-LC3 puncta (**Figure 3B**), as observed with the EGFP-LC3 puncta (**Figure 3A**).

Another hallmark of *spns1*-mutant fish is the striking induction of senescence-associated β-galactosidase (SA-β-gal), which is an endogenous lysosomal β-D-galactosidase detectable at pH 6.0 [12], [28]. Previously we demonstrated that an embryonic (or larval) senescence phenotype caused by specific gene mutations (or MO-mediated knockdowns) and also by stress is readily detectable via SA-β-gal staining of zebrafish embryos and larvae [12]. Additionally, we also tested another lysosomal hydrolase/glycosidase, α-L-fucosidase (α-fuc) that has been reported in mammalian cells as a novel sensitive biomarker, senescence-associated α-fuc (SA-α-fuc) [29]. We found that higher activity of SA-α-fuc, as well as of SA-β-gal, was detected in *spns1*-mutant fish, compared with wild-type control fish, with SA-β-gal being the more sensitive assay (**Figure S3C**; see also **Text S1 and S2**). We therefore examined the effect of *beclin 1* MO by staining with SA-β-gal in both *spns1* morphants and mutants. Consistent with the restored yolk clarity and reduced LC3 puncta observed with *beclin 1* knockdown in conditions of Spns1 deficiency, the *beclin 1* MO markedly decreased the intensity of SA-β-gal at 3.5 dpf (**Figure 3C** **and Figure S3B**), whereas control injections (water and standard control MO) did not significantly affect the SA-β-gal activity in *spns1* morphant and mutant animals (**Figure 3C** **and Figure S3B**). These results suggest that the aberrant SA-β-gal activity in *spns1*-defective animals coincides with autophagic initiation and its progression, and is accompanied by an increase in autolysosomes at the late autophagy stage.

While the excessive accumulation of autophagosomes and autolysosomes was observed in *spns1*-deficient animals, we anticipated that induction of apoptosis may be accompanied or preceded by the autophagic abnormality. We found, however, that such apoptotic induction was undetectable in *spns1* mutants and morphants (**Figure S4 and Figure S5**; see also **Text S1 and S2**). Acridine orange (AO) staining, which can correspond to the detection of acidified compartments [30], [31] as well as of apoptotic and necrotic cell death [32], [33], showed positive signals co-stained by LysoTracker in *spns1* mutants (**Figure S4**). However, when we performed a TUNEL assay for detecting DNA fragmentation associated with apoptosis, we found no staining upon knockdown of *spns1* (**Figure S5**), while the positive control of ultraviolet light (UV) irradiation produced a TUNEL-positive signal, as reported previously [34]. The UV irradiation-mediated apoptosis was slightly but not significantly inhibited by *beclin 1* knockdown (**Figure S6a**), which can fully suppress autophagy induced by UV (**Figure S6B**), suggesting that Beclin 1 plays a critical role in initiating autophagy, but is potentially dispensable for the induction of UV-mediated apoptosis in zebrafish embryos.

### Impact of the p53 status on Spns1 deficiency in zebrafish

It has been reported that cells deficient in Beclin 1 exhibit an elevated DNA damage response [35], along with an increase in reactive oxygen species (ROS) [36]. In addition, a reduction of p53 by proteasomal degradation has been documented under the condition of *beclin 1* knockdown [37]. The stress-responsive function of p53 still remains poorly understood with regard to how it is linked to autophagy impairment. In fact, although activated nuclear p53 can induce autophagy [38], it has also been reported that a removal of basal cytosolic p53 can stimulate autophagy [20]. We wondered which state of p53, if either, is involved in the Spns1 impairment. Moreover, since p53 activation is ordinarily thought to induce cellular senescence, which is also the case for zebrafish embryonic senescence [33], we suspected that the suppression of senescence by Beclin 1 depletion might be due to an intrinsic reduction in p53 [37]. We therefore investigated the requirement of p53 in the Spns1 deficiency-mediated senescence in zebrafish embryos under various experimental conditions through the genetic manipulations described below.

**Figure 4:**
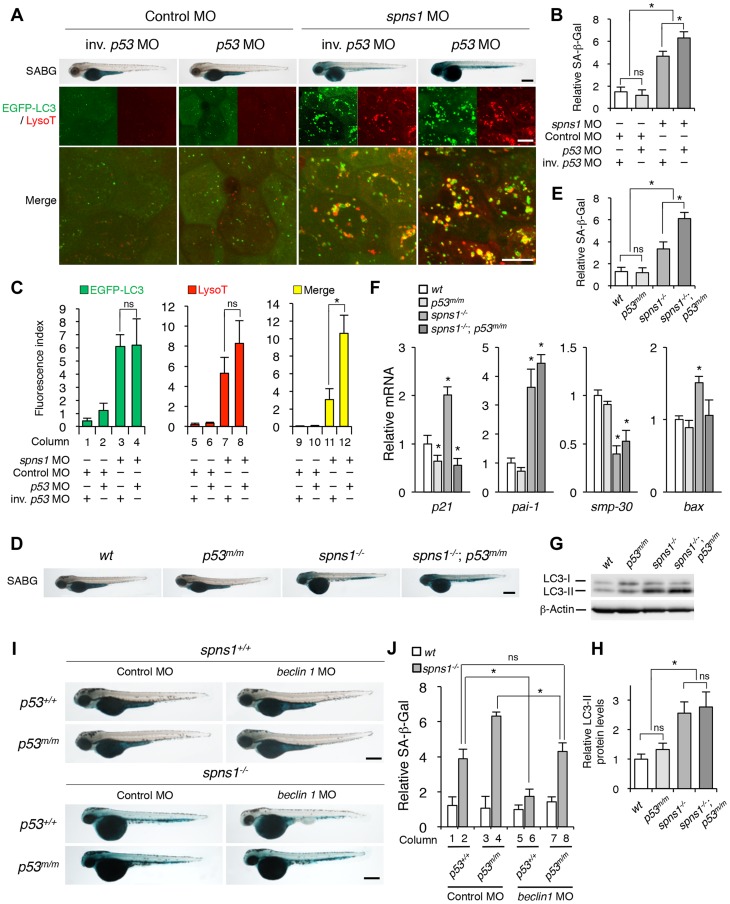
p53 depletion does not suppress but rather exacerbates Spns1 deficiency. (**A**) Effect of *p53* knockdown on embryonic senescence and autolysosome formation in *spns1* morphants. The impact of transient *p53* knockdown on SA-β-gal (SABG) induction, as well as on EGFP-LC3 and LysoTracker (LysoT) puncta, was determined in *spns1* morphants at 84 hpf, followed by the MO (4 ng/embryo) injections. Inverse-sequence *p53* MO (inv. *p53* MO) was used as a negative control for the original *p53* MO. Scale bar, 250 µm in the SABG images. Scale bar, 10 µm in the fluorescence images. (**B**) Quantification of the SA-β-gal intensities in MO-injected animals, as shown for the SABG images in (A). Quantification of data presented in the top row (SABG) in B (n = 12) is shown; the number (n) of animals is for each morphant. (**C**) Quantification of EGFP-LC3 and LysoTracker puncta in MO-injected animals shown in (A) (n = 9); the number (n) of animals is for each morphant. Three independent areas (periderm or basal epidermal cells above the eye) were selected from individual animals. (**D**) Effect of a *p53* mutation on embryonic SA-β-gal activity in the *spns1* mutant. The heritable impact of p53 and Spns1 on SA-β-gal induction was tested in each single gene mutant [*spns1^hi891/hi891^* (*spns1^−/−^*) or *tp53^zdf1/zdf1^* (*p53^m/m^*)] and double mutant *spns1^hi891/hi891^;tp53^zdf1/zdf1^* (*spns1^−/−^;p53^m/m^*) compared with wild-type (*wt*) animals at 84 hpf. Scale bar, 250 µm. (**E**) Quantification of the SA-β-gal intensities in *wt*, *tp53^zdf1/zdf1^*, *spns1^hi891/hi891^* and *spns1^hi891/hi891^;tp53^zdf1/zdf1^* animals, shown in (D). Quantification of data presented in panel D (n = 12) is shown; the number (n) of animals is for each genotype. (**F**) Quantitative RT-PCR analyses of senescence marker and/or mediator expression as well as p53-downstream target genes in *wt*, *tp53^zdf1/zdf1^*, *spns1^hi891/hi891^* and *spns1^hi891/hi891^;tp53^zdf1/zdf1^* at 72 hpf. Data are mean ±SD [n = 4 samples (3 embryos/sample) per genotype]. Asterisks denote significant changes compared to *wt* values. *p<0.05. (**G**) LC3 conversions in *p53* and *spns1*-mutant animals. Protein detection for the conversion/accumulation of LC3-I to LC-II was performed in the described mutant background animals in comparison with *wt* fish at 84 hpf. Western blot analysis using anti-LC3 antibody shows endogenous LC3 protein levels, which can confirm an increase of the total amount of LC3 in the *p53* mutant compared with *wt* fish. Increased LC3-II conversion/accumulation was detected in *p53* and *spns1* double-mutants as well as in *spns1* single-mutant fish. (**H**) The blotting band intensities of LC3-I, LC3-II and β-actin were quantified (n = 6), and the relative ratios between LC3-II/actin and LC3-I/actin are shown in the bar graph; the number (n) of animals is for each genotype. (**I**) *wt*, *tp53^zdf1/zdf1^*, *spns1^hi891/hi891^* and *spns1^hi891/hi891^;tp53^zdf1/zdf1^* embryos injected with *beclin 1* MO or control MO (12 ng/embryo) were assayed for SA-β-gal at 84 hpf. *beclin 1* MO-mediated suppression of SA-β-gal in *spns1^hi891/hi891^* animals was attenuated in the *p53* mutant background. Scale bar, 250 µm. (**J**) Quantification of the SA-β-gal intensities shown in (I). Quantification of data presented in H (n = 12) is shown; the number (n) of animals is for each genotype with MO. Error bars represent the mean ± S.D., *p<0.005; ns, not significant.

First, we examined the potential contribution of Spns1 and p53 separately in *spns1* and *p53* mutant fish backgrounds. We tested *spns1* MO and *p53* MO in *p53* mutants and *spns1* mutants, respectively (**Figure S7**). Unexpectedly, either *p53* mutation or knockdown enhanced, rather than suppressed, the senescence phenotype under the Spns1-defective conditions. This increased SA-β-gal activity that is induced by p53 suppression was further demonstrated by coinjection of *p53* MO and *spns1* MO into normal wild-type animals to rule out any background influence in the mutants (**Figure 4A** **, upper panels and B**).

Next, we performed coinjections of *p53* and *spns1* MOs into *Tg(CMV:EGFP-LC3)* fish to concurrently monitor the autophagic process with EGFP-positive LC3 aggregate formation, in addition to subsequent senescence induction (**Figure 4A** **, middle and lower panels, and C**). Upon transient knockdown, although the total EGFP fluorescence became brighter, the number of EGFP-LC3 puncta were only slightly increased by *p53* MO, compared with the control injected fish (**Figure 4C** **, columns 1 and 2**). On the one hand, enhanced LC3 puncta induction was observed when both MOs were coinjected, as similarly seen in the case of *spns1* MO injection only (**Figure 4C** **, columns 3 and 4**), suggesting that autophagy induction associated with Spns1 depletion does not require p53. On the other hand, there were more cumulative LysoTracker-positive aggregates (dysfunctional autolysosomes) colocalized with LC3 by the double knockdown than *spns1* knockdown alone, as EGFP-LC3 and LysoTracker double-positive yellow puncta were obviously increased by the p53 suppression in *spns1* morphants (**Figure 4A** **, middle panels, and C, columns 11 and 12**). Moreover, the enhancing effect of *p53* knockdown on senescence in *spns1* morphants was obviously seen (**Figure 4A** **, upper panels, and Figure S7**).

We further generated *spns1*-mutant fish harboring a *p53* mutation (*tp53^zdf1/zdf1^*), *spns1^hi891/hi891^;tp53^zdf1/zdf1^*, and confirmed that there was no requirement of normal p53 inheritance for the induction of embryonic senescence resulting from Spns1 deficiency, but instead there was an enhancement of SA-β-gal activity caused by the constitutive loss of wild-type p53 (**Figure 4D and E**). To further obtain robust hallmarks of senescence in zebrafish embryos, we examined the expression of other markers and/or mediators of senescence in *spns1*-defective animals. Quantitative RT-PCR (qPCR) as well as semi-qPCR in individual embryos demonstrated that the expression of *p21^waf1/cip1^* and *plasminogen activator inhibitor-1* (*pai-1*), which are known downstream targets of the p53 pathway [39], were upregulated in *spns1* morphants and mutants (**Figure 4F** **, and Figure S13**; see also **Text S1 and S2**). *Senescence marker protein-30* (*smp-30*) was downregulated in *spns1*-deficient animals compared with the corresponding controls. While the induction of *p21^waf1/cip1^* as well as *bax* was apparently regulated in a p53-dependent manner, both the *pai-1* induction and the *smp-30* reduction in *spns1* mutants were not influenced by the p53 defect.

We extended the analysis by monitoring the conversion of LC3-I into LC3-II among normal wild-type, *tp53^zdf1/zdf1^*, *spns1^hi891/hi89^*, and *spns1^hi891/hi891^;tp53^zdf1/zdf1^* fish through 4 dpf. Autophagy was minimally induced in *tp53^zdf1/zdf1^* fish based on detection of LC3-II conversion by immunoblotting, while the total amount of LC3 (LC3-I plus -II) was increased (**Figure 4G and H**). In *spns1^hi891/hi891^;tp53^zdf1/zdf1^* fish, the LC3-II conversion/accumulation was similar to that seen in *spns1-*mutant fish (**Figure 4G and H**). These results suggest that either decrease or loss of basal p53 enhances the Spns1 impairment, potentially by augmenting autophagy progression (but not initiation) and/or lysosomal biogenesis (i.e., subsequent autolysosomal formation and maturation).

We then proceeded to assess the epistasis among *spns1*, *beclin 1* and *p53*. We first confirmed that Beclin 1 suppression had no significant impact on *p53* morphants or *tp53^zdf1/zdf1^* fish (**Figure 4H and I** **, columns 1, 3, 5, and 7, and Figure S8**). Conversely, p53 depletion prevented the ability of *beclin 1* MO to suppress the appearance of the yolk opaqueness and senescence phenotypes of *spns1* mutants (**Figure 4H and I** **, and Figure S8**). Moreover, the *beclin 1* knockdown significantly suppressed the SA-β-gal activity in *spns1^hi891/hi891^;tp53^zdf1/zdf1^* fish to a similar extent as seen in standard control MO-injected *spns1^hi891/hi891^;tp53^+/+^* fish (**Figure 4H and I** **, columns 2 and 8, and Figure S8**). However, the reduction of the SA-β-gal activity was more obvious in *beclin 1* MO-injected *spns1^hi891/hi891^;tp53^+/+^* fish than in *spns1^hi891/hi891^;tp53^zdf1/zdf1^* fish (**Figure 4H and I** **, columns 6 and 8, and Figure S8**). Thus, basal p53 activity has a certain protective role(s) in preventing the deleterious phenotypes caused by genetic ablation of the *spns1* gene, by competing with Beclin 1-mediated autophagy progression.

Although basal p53 can contribute to attenuating the Spns1 deficiency conceivably through suppressing autophagic progression and lysosomal biogenesis, we also wondered whether “activated p53” in response to DNA damage (e.g., UV) has any impact on the Spns1 deficiency, based on the result that the UV irradiation activates and/or enhances autophagy in zebrafish embryos (**Figure S6B**). As anticipated, apoptosis was similarly induced in *spns1* mutants, compared with wild-type animals after UV treatment, whereas such apoptotic induction was almost undetectable under the *p53* mutant condition (**Figure S9A**; see also **Text S1 and S2**). The UV exposure apparently augmented both autophagic progress (i.e., GFP-LC3 puncta formation) and lysosomal biogenesis (i.e., LysoTracker-stained puncta) in *spns1* mutants only when functional p53 was present (**Figure S9B and C**).

A DNA damage response can be visualized as persistent foci of damaged nuclear DNA and its interacting proteins such as the phosphorylated histone variant, γH2AX [40]. DNA damage induced by UV treatment and the subsequent cell-cycle arrest in S phase were demonstrated by an increase of γH2AX intensity and a decrease of 5-bromo-2-deoxyuridine (BrdU) incorporation, respectively (**Figure S10**; see also **Text S1 and S2**). *spns1* mutants had a negligible difference in γH2AX levels but had a significant reduction of BrdU incorporation, irrespective of the p53 state (**Figure S10**), which is indicative of a slowdown of cell proliferation without apparent DNA damage. The immunostaining of a mitotic marker, phosphorylated histone H3 (pH 3) also showed a significant reduction in *tp53^+/+^*-*spns1*-mutant animals, even without UV treatment. There was a similar tendency of pH 3 reduction in non-irradiated *spns1;tp53*-double mutants, but it was not statistically significant (**Figure S11**). Embryonic SA-β-gal activity was consistently increased by the UV stimulation in both wild-type and *spns1*-mutant animals in the presence of p53 (**Figure S12**).

Finally, we extended our analysis to examine the expression profiles of *p21^waf1/cip1^*, *pai-1*, and *smp-30* as potential markers and/or mediators of senescence in *spns1*-defective animals (**Figure 4F** **and Figure S13**). *beclin 1* morphants did not show any significantly detectable changes in expression of these genes (**Figure S14A**). Importantly, however, the suppression of *beclin 1* significantly counteracted the impact of the *spns1* depletion by restoring expression of the *pai-1* and *smp-30* genes (**Figure S14A**). As described above, even in the absence of p53, the altered regulation of these two critical senescence markers was still detectable in *spns1*-deficient animals (**Figure S14B**), indicating that p53-independent regulation may be responsible for the expression of these genes. In contrast, the induction of *p21^waf1/cip1^*, *bax*, and *mdm2* genes in the *spns1*-defective condition was apparently p53 dependent and UV responsive, as confirmed by the level of their expression in *p53* mutants (**Figure S14B**). It is also important to note that expression of *ink4ab* (the zebrafish homolog of both *p15^ink4b^* and *p16^ink4a^*) was induced by UV treatment but not by Spns1 loss (**Figure S15**). Taken together, the upregulation of *pai-1* and *p21*, and the downregulation of *smp-30* in *spns1*-defective fish embryos are consistent with the induction of senescence characteristics in aging organisms [40], [41], [42], [43], [44], [45].

### Chemical modulation and monitoring of the autolysosomal acidification in *spns1*-mutant fish

**Figure 5:**
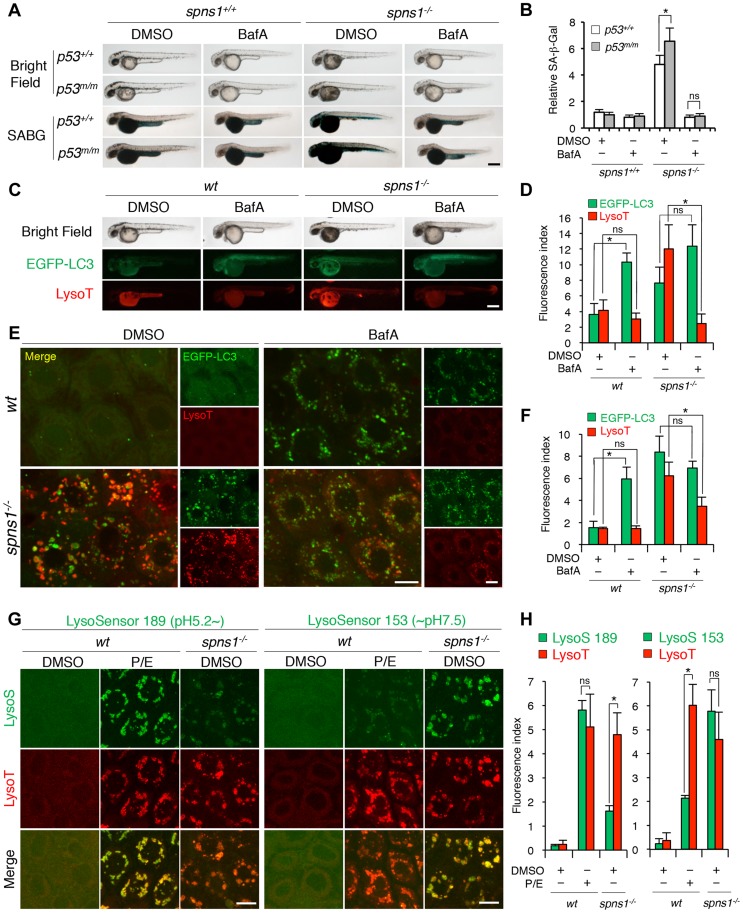
Acidity-dependent lysosomal biogenesis is rate limiting in *spns1*-mutant zebrafish. (**A**) Effect of bafilomycin A_1_ (BafA) on the yolk opaque phenotype (BF; bright field) and embryonic senescence (SABG; SA-β-gal) in the *spns1* mutant in the presence or absence of *p53* at 48 hpf. Normal wild-type (*spns1^+/+^;p53^+/+^*), *tp53^zdf1/zdf1^* (*p53^m/m^*), *spns1^hi891/hi891^* (*spns1^−/−^*) and *spns1^hi891/hi891^;tp53^zdf1/zdf1^* (*spns1^−/−^;p53^m/m^*) embryos at 36 hpf were incubated with BafA (200 nM) for 12 h, and stained with LysoTracker at 48 hpf, followed by SA-β-gal staining at 60 hpf. Scale bar, 250 µm. (**B**) Quantification of the SA-β-gal intensities shown in (A). Quantification of data presented in panel A (n = 12) is shown; the number (n) of animals is for each genotype with DMSO or BafA. (**C**) Gross morphology, EGFP-LC3 and LysoTracker intensities in wild-type (*wt*) and *spns1*-mutant animals treated with BafA shown at 48 hpf (12 h treatment starting at 36 hpf). Scale bar, 250 µm. (**D**) Quantification of the EGFP-LC3 and LysoTracker fluorescence intensities shown in (C). Quantification of data presented in the middle and bottom rows (green; EGFP, red; mCherry) in panel C (n = 12) is shown; the number (n) of animals is for each genotype with DMSO or BafA. (**E**) Intracellular autolysosome formation and lysosomal biogenesis in the BafA-treated *spns1* mutant. The samples analyzed in (C) were observed by using confocal microscopy at high magnification (×600). Scale bar, 10 µm. (**F**) Quantification of the EGFP-LC3 and LysoTracker fluorescence intensities shown in (E). Quantification of data presented for EGFP (green) and mCherry (red) signals in panel E (n = 6) is shown; the number (n) of animals is for each genotype with DMSO or BafA. Three independent areas (periderm or basal epidermal cells above the eye) were selected from individual animals. (**G**) Insufficient intracellular acidity constituent in the *spns1* mutants. Using two different acidic-sensitive probes, LysoSensor 189 and neutral-sensitive LysoSensor 153 (green), in combination with LysoTracker (red), *wt* and *spns1*-mutant animals showed detectable signals when stained at 72 hpf. In *spns1*-mutant animals, autolysosomal and/or lysosomal compartments were more prominently detectable by LysoSensor 153 than by LysoSensor 189, at the cellular level with enhanced signal intensity of these enlarged compartments. In stark contrast, the cellular compartments in *wt* fish treated with pepstatin A and E-64-d (P/E) (12 h treatment from 60 hpf through 72 hpf) were more prominently detectable by LysoSensor 189 than by LysoSensor 153 under the identical LysoTracker staining conditions. Of note, these autolysosomal and lysosomal compartments in *spns1* mutants, as well as in *wt* animals treated with pepstatin A and E-64-d, may still retain some weak (higher pH) and strong (lower pH) acidity, respectively, as short-term BafA treatment (for 1 h between 71 and 72 hpf) can abolish the acidic compartments stained by both LysoSensor and LysoTracker (**Figure S17C and D**). Scale bar, 10 µm. (**H**) Quantification of the LysoSensor (189 and 153) and LysoTracker fluorescence intensities shown in (G). Quantification of data presented for LysoSensor (green) and LysoTracker (red) signals in panel G (n = 6) is shown; the number (n) of animals is for each genotype with DMSO or pepstatin A and E-64-d (P/E). Three independent areas (periderm or basal epidermal cells above the eye) were selected from individual animals. Error bars represent the mean ± S.D., *p<0.005; ns, not significant.

Chemical genetic approaches are emerging in the zebrafish model system, and increasing numbers of chemical compounds are currently available for examining autophagic regulation [46], [47], [48]. We determined the effects of several chemical compounds and selective modulators of autophagy on Spns1 deficiency to see if any chemical(s) ameliorates or exacerbates the Spns1 phenotypes of zebrafish embryos. Of the chemicals tested, bafilomycin A_1_ (BafA) and other proton pump inhibitors (PPIs) such as the acid reducer omeprazole stood out due to their apparent inhibitory effect on overall phenotypic deterioration in *spns1* animals (**Figure 5A, B** **and Figure S16**). BafA is a specific inhibitor of vacuolar-type H^+^-ATPase (v-ATPase), and inhibits lysosomal acidification, slowing or blocking degradation of LC3-II within autolysosomes as well as inhibiting the fusion between autophagosomes and lysosomes [49], [50], and subsequently it also enhances EGFP-LC3 puncta accumulation corresponding to mammalian autophagosomes [27]. Consistently, we found that BafA significantly increased the formation of cellular LC3 puncta as well as their gross EGFP intensity in wild-type zebrafish (**Figure 5C–F**). Intriguingly, both the progression of yolk opacity and SA-β-gal activity in *spns1* mutants during the time period of 36–60 hpf were entirely suppressed by BafA treatment (**Figure 5A and B**). While EGFP-LC3 puncta signals in BafA-treated *spns1* mutants did not appear significantly different compared with those in untreated counterparts, LysoTracker-positive compartments in cells were reduced by BafA treatment (**Figure 5E and F**), similar to the result seen with whole animal staining (**Figure 5C and D**). This is likely due to ‘prior’ alkalinization in lysosomes/autolysosomes and reduction of their biogenesis (**Figure 5E and F**). Importantly, these effects of BafA on *spns1*-mutant animals were essentially unaltered under the *p53*-depleted condition. Thus, BafA-induced pre-alkalinization might compensate for vulnerability of the *spns1* mutants lacking basal p53 activity (**Figure 5A**).

BafA specifically binds to subunit c of the v-ATPase and thereby inhibits its enzymatic and proton-pump activity, but it has been shown that the concentration used and the duration of treatment with this drug are fairly critical to observe this effect [49]. In addition, BafA may have other off-target effects [51]. Therefore, we specifically knocked down the v-ATPase subunit gene *atp6v0c* by using a MO, whose effectiveness had already been demonstrated [52]. We obtained comparably consistent outcomes for the ameliorative effect of *atp6v0c* knockdown on the Spns1 deficiency (**Figure S17A and B**). In addition, we found that three other PPIs (omeprazole, lansoprazole, and pantoprazole), which can all interfere with the v-ATPase [53], [54], could also suppress the senescence phenotype in *spns1* mutants (**Figure S17C**).

We further utilized LysoSensor dye to monitor acidification levels in lysosomes and autolysosomes, to verify that possible pre-alkalinization by BafA treatment or direct *atp6v0c* knockdown can efficiently suppress the *spns1*-mutant phenotypes. In contrast to the LysoTracker probes, which exhibit fluorescence that is largely independent of the pH level, the LysoSensor reagents can show a pH-dependent increase in fluorescence intensity upon acidification [55]. The neutral pH-sensitive LysoSensor 153 fluoresces optimally at pH 7.5, while the acidic pH-sensitive LysoSensor 189 fluoresces optimally at pH 5.2. When these probes (green) were used in combination with LysoTracker (red), we found a much stronger signal with LysoSensor 153 than with LysoSensor 189 in *spns1*-mutant animals (**Figure S18A and B**), which was also quite obvious at the cellular level (**Figure 5G and H**). By contrast, treatment of wild-type animals with lysosomal protease inhibitors, pepstatin A and E-64-d, which allows the retention of intact autolysosomal/lysosomal acidity while preventing autolysosomal maturation and turnover, showed highly acidic vesicles stained by LysoSensor 189, rather than by LysoSensor 153 (**Figure 5G and H**). Lysosomal compartments in *spns1* mutants may still retain some weak acidification allowing lysosomal biogenesis and subsequent autophagosome-lysosome fusion, as short-term treatment (for 1 h) with BafA can completely abolish the acidic compartments stained by LysoSensor and significantly reduce the LysoTracker-positive signals (**Figure S18C and D**).

Finally, we examined the colocalization of EGFP-LC3 puncta and lysosomes in wild-type fish in the presence of BafA or pepstatin A and E-64-d, compared to that in the *spns1^hi891/hi891^* fish (**Figure S19A and B**). In wild-type animals, BafA caused the accumulation of EGFP-LC3 and colocalization of EGFP-LC3-mCherry-LC3 signals (**Figure S19C**), but no accumulation of LysoTracker, indicating a block in fusion of autophagosomes with lysosomes (**Figure S19B**). Inhibition of lysosomal hydrolase activity with pepstatin A and E-64-d resulted in accumulation of lysosomes (red) and autolysosomes (yellow by overlapping EGFP-LC3 and LysoTracker) (**Figure S19B**). In contrast, the *spns1^hi891/hi891^* fish (**Figure S19A**) and their cells (**Figure S19B and C**) displayed both an accumulation of autolysosomes (yellow by overlapping EGFP-LC3 and LysoTracker) and autophagosomes (yellow by overlapping of EGFP-LC3 and mCherry-LC3) without any inhibitors, again indicating defects in both fusion of autophagosomes with lysosomes and autolysosomal maturation. Collectively, these results demonstrate that the appearance of deleterious changes in *spns1* animals is due to aberrant autophagic progression caused by impaired suboptimal acidification in malformed autolysosomes, and that p53 may also be involved in the process of both lysosomal and autolysosomal pathogenesis in Spin1 deficiency.

## Discussion

We demonstrated that loss of Spns1 leads to defects in autophagic and lysosomal homeostasis in zebrafish, and the tumor suppressors Beclin 1 and p53 are differentially involved in autophagy and senescence pathways regulated by Spns1. A reduction of Beclin 1 as an autophagy regulator can attenuate the Spns1 defect, whereas a decrease/loss of basal p53 activity, as well as activated p53 by DNA damage, augments it and exacerbates the deleterious phenotype in zebrafish. If both Spns1 and p53 were abrogated, the Beclin 1 reduction was no longer effective in suppressing the *spns1*-mutant phenotypes sufficiently, whereas v-ATPase reduction was robust enough to suppress the phenotypes regardless of p53 state.

Importantly, we have successfully generated valuable zebrafish tools by crossing the fluorescent protein-tagged LC3- and GABARAP-transgenic lines with the *spns1*-mutant line to monitor real-time alterations of autophagic abnormalities *in vivo*. Vertebrates have approximately seven Atg8 homologs [56], and the best studied of these is LC3. GABARAP shows many similarities with LC3, but its conjugation is only mildly affected by starvation, and under certain conditions conjugation may be activated independent of target of rapamycin (TOR) inactivation [57], [58]. We have found, however, an indistinguishable behavior between LC3 and GABARAP in the transgenic animals harboring either *spns1* mutation or depletion, although it has been suggested that LC3 and GABARAP differentially act in autophagosome biogenesis [59].

The evolutionarily conserved autophagy gene *beclin 1* (*vps30/atg6*) is frequently inactivated at one locus in several cancers [60], [61]. Studies in mice have also demonstrated that *beclin 1* is a haploinsufficient tumor suppressor [17], [62]. It has been demonstrated that Spns1-loss-associated EGFP-LC3 puncta accumulation in cells, which reflects autophagic progression by autophagosome formation, is suppressed by the depletion of Beclin 1, Atg7, or Ulk1, as well as by treatment with a PtdIns3K inhibitor, 3-methyladenine [16]. Consistently, we also demonstrated that *beclin 1* morphants were resistant to forming LC3 puncta induced by Spns1 deficiency in zebrafish. However, once both *spns1* and *p53* were depleted, the *beclin 1* knockdown was no longer effective enough to suppress the punctate accumulation of LC3 as well as the mutant hallmarks of both yolk opaqueness and embryonic senescence characteristic of Spns1 deficiency in zebrafish.

*p53* is one of the most commonly mutated tumor suppressor genes found in many types of human cancers [63]. We found that the loss of basal p53 compromises the ability to properly adjust autolysosomal formation, and exacerbated the *spns1* deficiency, while *beclin 1* knockdown can ameliorate it by suppressing the early stage of autophagy. p53 has been linked to the regulation of autophagy, but the exact nature of its role still remains controversial. On the one hand, onocogenic and genotoxic stress events result in p53 stabilization and activation, which can stimulate autophagy through both transcription-independent mechanisms (e.g., AMP-activated protein kinase; AMPK activation and TOR inhibition) and transcription-dependent mechanisms (e.g., transcriptional upregulations of PTEN, tuberous sclerosis complex 1/TSC1 and damage-regulated autophagy modulator/DRAM1) [64]. On the other hand, it has been reported that genetic or chemical inhibition of basal cytoplasmic p53, or proteasomal depletion of p53 during starvation and/or endoplasmic reticulum stress, activates autophagy through transcription-independent mechanisms involving AMPK activation and TOR inhibition [20]. Loss of p53 may lead to homeostatic imbalance in cells, such as induction of bioenergetic compromise, increased ROS, and/or defective cell-cycle checkpoints, which can lead to autophagy induction. Thus, p53 depletion may promote or enhance autophagy indirectly as a result of imbalanced metabolic stress conditions. This therefore suggests that p53 maintains cellular homeostasis by adjusting the rate of autophagy in a context-dependent manner, as circumstances require.

**Figure 6:**
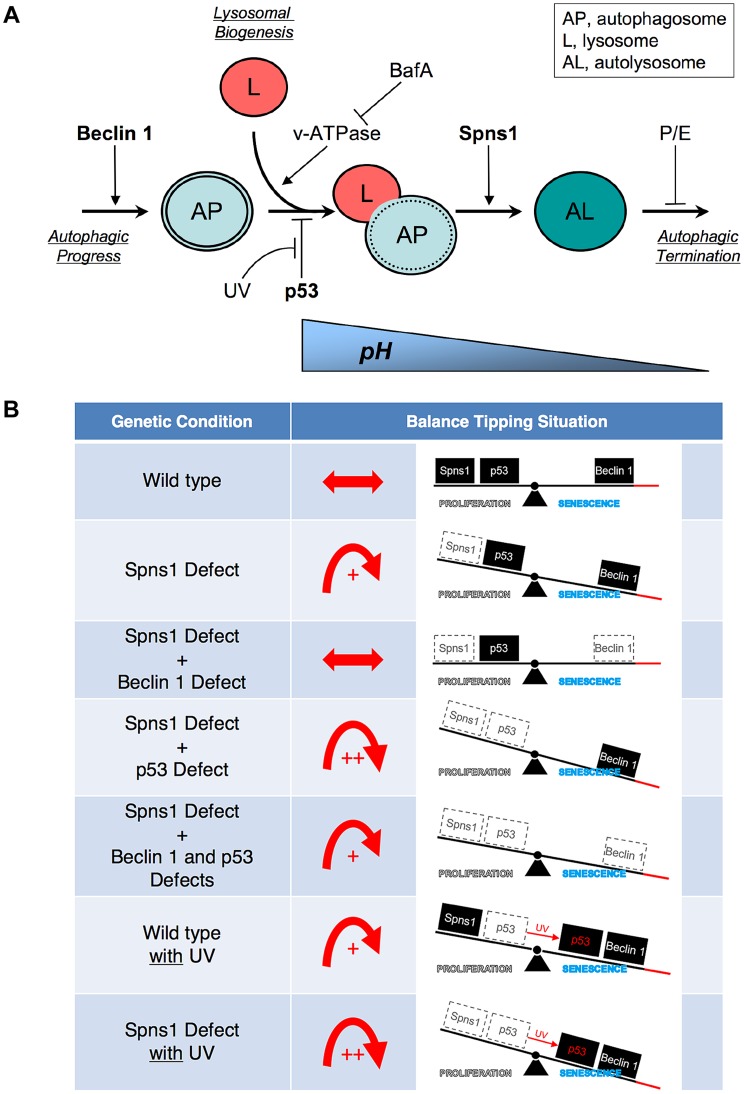
Schematic model for Spns1 function under the control of the network module of autophagy-senescence signaling cascades differentially regulated through Beclin 1 and p53. (**A**) Beclin 1 is essential for the early stage of autophagy and its depletion suppresses the Spns1 defect by blocking the ‘autophagic process’ and its progression. BafA can decelerate ‘lysosomal biogenesis’, which subsequently presumably prevents autophagosome-lysosome fusion, through the inhibition of the v-ATPase, and contributes to amelioration of the Spns1 defect at least temporarily. Basal p53 activity may suppress the intersection between the ‘autophagic progress’ and ‘lysosomal biogenesis’ where the Beclin 1 depletion was not sufficient, but the v-ATPase inhibition was still effective enough, to compete with the p53 loss to suppress the Spns1 deficiency. By switching the basal p53 state to the activated version with UV irradiation, p53 can promote autophagy. Spns1 might be a gatekeeper of autolysosomal maturation followed by lysosomal biogenesis. It remains unknown how p53 can mechanistically be linked to the lysosomal ‘efflux’ function of Spns1 as well as the lysosomal ‘influx’ function of v-ATPase, and further investigations will be required to explore this connection. (**B**) Roles of Spns1, p53 and Beclin 1 in senescence equilibrium. Loss of Spns1 leads to an imbalance in homeostasis and increased senescence. This effect can be ameliorated by concurrent knockdown of Beclin 1. p53 has a comparatively less dramatic impact on Spns1-loss-induced embryonic senescence. When in the “basal” state, p53 helps retain equilibrium. When p53 is “activated” by UV irradiation, a modest increase in senescence is observed. The higher level of senescence is seen during loss of Spns1 in the absence of basal p53 or in the presence of activated p53. During loss/knockdown of all three genes (*spns1*, *p53* and *beclin 1*), a state of moderate senescence is observed. An increase in senescence is accompanied by a p53-dependent decrease in cellular proliferation.

Intriguingly, Spns1-loss-induced embryonic senescence (SLIES) represents an atypical senescence response that is distinct from p53-induced senescence and can be suppressed by autophagy inhibition mediated through *beclin 1* knockdown (**Figure 6**). Since the Beclin 1 suppression may lead to reduction in the level of p53 [37], and then might subsequently prevent SLIES, we intensively examined the effect of p53 depletion on SLIES. To our initial surprise, SLIES cannot be suppressed by the loss of p53 at all, but is rather enhanced. This seems to contradict the conventional role of p53 as an inducer of cellular senescence in various contexts including the zebrafish model [33], [65]. However, given recent evidence of a certain anti-aging mechanism by p53 in mice and p53-mediated suppression of senescence in cells [66], [67], it might not be surprising that p53 can also act both as a suppressor of senescence and of autophagy in some contexts, although the exact molecular mechanism remains elusive at this point. In addition, there remains a p53-independent cellular senescence mechanism that still depends on its authentic downstream target, p21^Waf1/Cip1/Cdkn1a^, among others, such as Arf and p27^Kip1/Cdkn1b^ triggered by Skp2 inactivation [68]. Moreover, a recent report indicated that p21^Waf1/Cip1/Cdkn1a^ also has a tissue-selective and context-dependent modulation of senescence in BubR1 progeroid mice [69]. In addition, most recently, SA-β-gal-positive “developmental senescence” observed in mice, which shares some, but not all, regulatory pathways detectable in adults, was shown to involve the activation of p21^Waf1/Cip1/Cdkn1a^ in the absence of a DNA damage response and any alteration in p53, p16^Ink4a^, or p19^Arf^ [70], [71]. Interestingly, we found that in *spns1*-deficient fish embryos, the upregulation of *p21* and *pai-1* expression and the downregulation of *smp-30* expression were detected without a DNA damage response. Further investigation and elucidation of their functional roles as senescence mediators or attenuators will be required to determine how they are responsible for SLIES.

p53 is also well known for its pro-apoptotic cell death-inducing activities, but it can conversely possess pro-survival effects, particularly under mild stress conditions [72], [73], [74]. In zebrafish embryos, however, we determined that SLIES occurs regardless of p53-mediated impacts on apoptotic cell death and/or the cell-cycle checkpoint response as well. Thus, *spns1*-mutant animals show a new type of developmental senescence that can be triggered by autophagic initiation followed by autolysosomal fusion in the absence of the authentic senescence moderator p53, while basal p53 and activated p53 can play contrasting roles; attenuation in SLIES and mediation in apoptosis, respectively.

“Activated p53” is not specifically involved in the *spns1*-ablated condition but can generally induce and/or augment the deleterious condition caused by the DNA damage response and apoptosis. In contrast, “basal p53” may have an antagonistic effect on lysosomal biogenesis (or autolysosomal maturation) rather than on the autophagic progress in the absence of Spns1. Alternatively, the p53 status may rather influence endosomal-lysosomal homeostasis where Spns1 is primarily involved. It should be noted that p53 may be involved in lysosomal stabilization [75], as well as in various metabolic changes and the regulation of energy metabolism including aerobic glycolysis (the Warburg effect) in which the lysosome is also engaged for degradation [38].

Our preliminary observation suggests that SLIES and the yolk opaqueness hallmarks of *spns1* embryos are only mildly affected by chemical (e.g., rapamycin)-mediated autophagy induction. This may be a reflection of the consistent outcome of already attenuated TOR (re)activation due to impaired autophagic lysosome reformation by Spns1 deficiency, as has been demonstrated in mammalian cells and flies [16], [76]. We are also wondering if basal p53 depletion may have any effect on autophagy enhancing activity independent of or different from the rapamycin-sensitive TOR pathway.

Of note, rather than simple depletion of wild-type p53, the p53 mutant (*tp53^zdf1/zdf1^*) fish used here retain an accumulation of the mutant p53 protein (p53^M214K^) [77], which corresponds to the human p53^M246K^ mutant protein whose function is completely abolished [78]. A recent study suggests that this mutant p53 protein is degraded through chaperone-mediated autophagy (CMA) in a lysosome-dependent fashion [79]. Thus, the regulation of the stability of mutant p53 differs from that of wild-type p53. There is a possibility of activating the CMA pathway by inhibiting (macro)autophagy, to specifically promote the degradation of mutant p53, under a nutrient-starved condition. Therefore, it is also important to examine any involvement of the Spns1 function in the protein stability of mutant p53, whether the Spns1 defect selectively activates the CMA pathway for the removal of mutant p53 or not.

Altogether, our present results support the notion that the interruption of the intrinsic nutrient supply through autophagy, supposedly from yolk in zebrafish embryos and larvae [80], may lead to profound energetic exhaustion under the aberrant autolysosomal condition resulting from Spns1 deficiency, and this effect is dependent on the p53 state.

Since BafA can inhibit the import of H^+^ through the v-ATPase into the lysosome lumen, while the Spns1 defect presumably prohibits the symport of H^+^/sugar by loss of its function, it was anticipated that BafA might at least temporarily rescue the *spns1*-mutant animals, by restoring the balanced acidity condition of autolysosomes and/or lysosomes, as well as subsequent autophagosome-lysosome fusion. In fact, we found that BafA effectively inhibited the progression of both yolk opacity and embryonic senescence that appeared in *spns1* mutants. Moreover, a direct depletion of the v-ATPase subunit c (a direct target of BafA) by MO recapitulated the restorative effect on the mutant animals. Importantly, the lysosomal pH of *spns1* mutants was found to be less acidic, suggesting that protons may pass through the membrane via other H^+^-coupled transporters and/or channels such as lysosomal amino acid transporter 1 (LYAAT-1/SLC36A1) [81], chloride channel 7 (CLCN7) [82], and cystic fibrosis transmembrane conductance regulator (CFTR) [83].

It should be noted that SA-β-gal is acid β-D-galactosidase, a lysosomal glycoside hydrolase (glycosidase), which catalyses the hydrolysis of β-galactosides into monosaccharides [28], and its substrates also include various glycoproteins, gangliosides (glycosphingolipids), lactose, and lactosylceramidases [84], [85]. The aberrant increase of the *in situ* SA-β-gal activity induced by Spns1 deficiency indicates that such a glycosidase product itself can be preserved in autolysosomes and/or lysosomes, but may not function properly *in vivo* without an essential permease(s) to transport degradation products that need to be delivered into the cytoplasm as energy sources.

The extent to which our current observations of Spns1 functions during early development pertain to actual aging and age-related disease situations remains to be rigorously determined under both physiological and pathological conditions in animals. However, an increase in the abundance of the lysosomal hydrolases is presumably linked to the increased lysosomal biogenesis observed in senescent cells. Indeed, cumulative evidence suggests that an increased number of lysosomes and elevated lysosomal activity have been associated with replicative senescence [85]. Thus, the current finding suggests that temporal suppression of autophagy through Beclin 1 and/or v-ATPase by approved therapeutics (e.g., omeprazole) may be an effective therapeutic approach in the prevention of autophagic impairments similar to the Spns1 deficiency (**Figure 6**). Similar intervention has been demonstrated successfully in a mouse model of Pompe disease, a lysosomal glycogen storage disorder [86].

## Materials and Methods

### Zebrafish maintenance and ethics

Zebrafish (AB and *casper* strains) were maintained under a 14:10 h light/dark cycle and fed living brine shrimp twice daily. Brine shrimp were presented using 1 mL pipettes (about 0.75 mL brine shrimp per 20 fish). Flake food was also given every other day in proportion to the number of fish in the tanks. A continuously cycling aquarium system was used to maintain water quality (Aquatic Habitats Inc.). Zebrafish embryos were collected from pairwise matings of adults and raised at 28.5°C. The embryos to be used in the experiments were then staged by hours post fertilization (hpf) at 28.5°C and also by morphological appearance for experiments [87]. All animal experiments were approved by and conducted in accordance with the guidelines established by the Institutional Animal Care and Use Committee (IACUC) at The Scripps Research Institute, IACUC approval number 09-0009.

### Confocal microscopy and imaging

Zebrafish embryos (in the case of the AB fish line) were transferred into 0.003% (w/v) 1-phenyl-2-thiourea (PTU) prior to 24 hpf to prevent pigmentation. Embryos or larvae were then mounted live in water containing 0.16 mg/ml tricaine (Sigma, A5040) for imaging. Images were taken using the FluoView 1000 confocal laser scanning microscope system (Olympus) with a 60× objective lens). Since EGFP- or mCherry-LC3 and EGFP-GABARAP showed both a uniform cytosolic signal and more intense spots, threshold values were set to reduce the cytosolic signal and identify the more intense dots. The same threshold value was applied for all samples in the indicated experiments. The extent of colocalization between LysoTracker and LysoSensor signals and EGFP- or mCherry-LC3 and EGFP-GABARAP dots was quantified in three independent visual fields from three independent embryos. All values are represented as mean ± standard deviation (S.D.). Mounted animals were photographed using each specific fluorescent signal by confocal laser microscopy. Fluorescence intensities were quantified using Adobe Photoshop over a color range that was chosen according to 25 additive color selections of regions that showed visually positive signals. For analyses of cells within the zebrafish embryos, these regions were selected in each actual embryo only and not in the yolk. Following pixel selection, a fuzziness setting of 64 was used, and the chosen pixel number was determined using the image histogram calculation.

### Morpholino oligonucleotides

Morpholino oligonucleotides (MOs) were designed and synthesized by Gene Tools, LLC (Philomath, OR). The sequence of the *beclin 1* MO is 5′-CATCCTGCAAAACACAAATGGCTTA-3′, which overlaps the intron-exon boundary at the 5′-splice junction of exon 4 in the zebrafish *beclin 1* gene. The sequence of the standard control MO is 5′-CCTCTTACCTCAGTTACAATTTATA-3′. MOs were resuspended in sterile water at a concentration of 1 mM as stock solutions. For microinjection into embryos, the stock solutions (1 mM) were diluted to 125, 250, 500, and 750 µM. A 10 nl volume of each MO solution was injected into the yolk during the one-cell stage. All other MO sequences have been reported previously [8], [12], [52], [88], except Inverse-sequence *p53* MO (inv. *p53* MO); 5′-GTTAAGAACGTTTCGTTACCGCG3′.

### MitoTracker, LysoTracker, LysoSensor and DQ Red BSA staining

The vital mitochondrial and lysosomal dyes MitoTracker Green FM (Invitrogen; molecular probes, M7514), LysoTracker Red DND-99 (Invitrogen; molecular probes, L7528), LysoSensor Green DND-189 (Invitrogen; molecular probes, L7535) and LysoSensor Green DND-153 (Invitrogen; molecular probes, L7534) were diluted to final concentrations of 1 µM, 10 µM, 1 µM and 1 µM, respectively, in E3 medium (5 mM NaCl, 0.17 mM KCl, 0.33 mM CaCl_2_, 0.33 mM MgSO_4_), and pre-warmed to 28.5°C. Each dye was then added to an equal volume of fresh water on embryos and incubated at 28.5°C in the dark for 30 min to 1 h. Embryos were then rinsed four times in fresh E3 medium before imaging. DQ Red BSA (Invitrogen; molecular probes, D12050) was diluted to a final concentration of 0.5 mg/ml in E3 medium, directly injected into the yolk sac at 72–84 hpf, and subjected animals were incubated for 4 h prior to observation by microscopy.

### Transmission electron microscopy

Zebrafish larvae were fixed in 4% paraformaldehyde, 2.5% glutaraldehyde, 0.02% picric acid, 0.1 M Na cacodylate buffer, washed and fixed in 1% osmium tetroxide in 0.1 M Na cacodylate buffer. They were subsequently treated with 0.5% tannic acid followed by 1% sodium sulfate. The pellets were treated with propylene oxide and embedded in Epon/Araldite. Thin sections (70 nm) of the pelleted samples were cut on a Reichert Ultracut E (Leica, Deerfield, IL) using a diamond knife (Diatome, Electron Microscopy Sciences, Hatfield, PA), mounted on parlodion-coated copper slot grids and stained in uranyl acetate and lead citrate. Sections were examined on a Philips CM100 transmission electron microscope (FEI, Hillsbrough, OR). Images were documented and measurements were taken using a Megaview III CCD camera (Olympus Soft Imaging Solutions, Lakewood CO). Transverse sections were obtained through the trunk muscle region, the yolk and the eye region.

### RNA isolation and RT-PCR analysis for zebrafish *beclin 1*

RT-PCR analysis of a single zebrafish embryo was performed to determine the effects of the splice-block MO for the zebrafish *beclin 1* gene. Total RNA was extracted from 24–48 hpf embryos injected with control MO, *beclin 1* MO, or *beclin 1* plus *spns1* MO, using TRIzol reagent according to the manufacturer's protocol (Invitrogen). cDNA was synthesized using M-MLV reverse transcriptase (Promega), followed by PCR with Ex*Taq* (Takara). For semi-quantitative analysis, the linear amplification ranges were then determined for each of the primer sets. PCR primers used to amplify the fragments of the zebrafish *beclin 1* gene were designed using a web-based primer design tool, PrimerQuest (Integrated DNA Technology, Inc.) (*zbeclin 1* EX3 forward primer; 5′-CAAACAAGATGGCGTGGCTCGAAA-3′, *zbeclin 1* EX4 forward primer; 5′-GTGGAACTATGGAGAACTTGAGTCGCA-3′, and *zbeclin1* EX7 reverse primer; 5′-TCCAACTCCAGCTGCTGTCTCTT-3′). The amplified products were validated by sequencing. As controls for these PCR analyses, *ef1α* and *β-actin* were examined. The forward and reverse primers used to amplify *ef1α* were 5′-ACCACCGGCCATCTGATCTACAAA-3′ and 5′-ACGGATGTCCTT GACAGACACGTT-3′, respectively, and for *β-actin* were 5′-CCCAGACATCAGGGAGTGAT-3′ and 5′-CACCGATCCAGACGGAGTAT-3′, respectively. For amplification by PCR, the initial denaturing step at 94°C for 5 min was followed by 18–25 amplification cycles of 30 sec at 94°C; 30 sec at 60°C; 60 sec at 72°C, and a final extension period of 10 min at 72°C. Amplified products were separated on a 1.5% agarose gel stained with ethidium bromide and the bands were visualized and recorded using a Multi Image Light Cabinet (Cell Bioscience). Other PCR primers, parameters and conditions are summarized in **Supplemental Table S1 and S2**.

### SA-β-gal assay and quantification

Zebrafish embryos and larvae at 48–72 dpf were washed three times in phosphate buffered saline (PBS) and fixed overnight in 4% paraformaldehyde with PBS at 4°C. After fixation, the samples were washed three times in PBS, pH 7.5, twice again in PBS, pH 6.0 at 4°C, and then incubated at 37°C (in the absence of CO_2_) for 12–16 h with SA-β-gal staining solution (5 mM potassium ferricyanide, 5 mM potassium ferrocyanide, 2 mM MgCl_2_ in PBS at pH 6.0). All animals were photographed under the same conditions using reflected light with a macro microscope, AZ100 (Nikon). SA-β-gal activity in each animal was quantified using a selection tool in Adobe Photoshop software for a color range that was chosen using 25 additive color selections of regions that showed visual SA-β-gal staining. For analyses of embryos, these regions were selected in each embryo proper only and not in the yolk in order to exclude variability in the initial yolk volume and yolk consumption levels over time. Since the yolk stains much more intense for SA-β-gal at all stages of development than any other embryonic tissues in general, it was desirable to eliminate this as a source of variability. Following pixel selection, a fuzziness setting of 14 was used, and the chosen pixel number was determined using the image histogram calculation.

### Immunoblotting

Embryos were dechorionated, deyolked and homogenized in RIPA buffer. Protein concentrations of embryo lysates were determined using the bicinchoninic acid (BCA) protein assay. The lysates were mixed with equal volumes of 2× SDS sample buffer, heated at 95°C for 2 min, and resolved on 12.5% or 15% gels. After transfer, the polyvinylidene difluoride membranes were incubated with primary antibodies [anti-LC3A/B (Cell Signaling Technology, Inc., #4108), anti-β-actin (Cell Signaling Technology, #4967), or anti-GFP (Life Technologies, A11122) antibody], diluted in TBST overnight at 4°C. After washing, the blot was then incubated with a secondary anti-rabbit horseradish peroxidase-conjugated antibody (Cell Signaling Technology, #7074) at room temperature for 1 h and visualized using an ECL kit (Perkin Elmer) in accordance with the manufacturer's instructions.

### Generating transgenic zebrafish

To generate transgenic zebrafish expressing mCherry-tagged LC3, the corresponding expression construct pminiTol2-mCherry-LC3 was generated using the QuikChange Site-Directed Mutagenesis Kit (Stratagene) in accordance with the manufacturer's instructions. pT3TS-Tol2 was linearized by XbaI and transcribed with T3 RNA polymerase using the Ambion mMESSAGE mMACHINE kit (Ambion, AM1348) to produce Tol2 transposase mRNA. Approximately 5 nl of the mixture of plasmid DNA (100 ng/µl) (pminiTol2-mCherry-LC3) and 50 pg of Tol2 transposase mRNA (100 ng/µl) were coinjected into newly fertilized embryos at the one-cell stage to produce transgenic fish. Injected embryos were raised to adulthood and out-crossed to wild-type fish to identify germline-transmitted transgenic founders (F_0_) as described previously [22]. Positive founders were determined by screening F_1_ embryos for visible mCherry expression. The mCherry-positive offspring were then allowed to grow to maturity for further experiments.

### Chemical treatments

Bafilomycin A_1_ (BafA) (LC Laboratories, B-1080), omeprazole, lansoprazole, and pantoprazole (Sigma, O104, L8533, and P0021, respectively) treatment was performed from 36 through 48 hpf or 48 through 60 hpf in E3 medium at 28.5°C in 12- or 6-well plates. The chemicals dissolved in DMSO were added to the embryo water (E3 medium) at the final concentrations of 200 nM for BafA and 25 µM for lansoprazole, omeprazole and pantoprazole. Pepstatin A (Fisher BioReagent, BP26715) and E-64-d (Enzo Life Sciences, BML-PI107) treatment was administrated from 60 through 72 hpf for 12 h in E3 medium at 28.5°C in 12- or 6-well plates. These reagents were both dissolved in DMSO and added to the embryo water (E3 medium) at the final concentration of 5 µg/ml.

### Quantitative analysis and statistics

Data processing and statistical analyses were performed using Statistical Package for the Social Sciences (SPSS) version 14.0. This software was used to generate each of the graphs shown in the text to perform statistical tests where appropriate.

## Supporting Information

Figure S1 Autophagic abnormalities and survival in *spns1*-mutant fish and worms. (**A**) Representative transmission electron microscopy images of normal *wt* or *spns1*-mutant fish larvae at 84 hpf. Compared with wild-type (*wt*) control (left), the *spns1* mutant (*spns1^−/−^*) (right) accumulates abnormal cytoplasmic inclusions at the hypodermal regions adjacent to yolk sac (ys) (upper two panels) or melanophores (me) (middle two panels), and in the retinal pigment epithelium containing melanophores (me) (lower two panels). Arrows indicate cytoplasmic membranous inclusions. In the right-upper panel, the inset shows a magnified image of the cytoplasmic inclusion surrounded with a dotted square. Scale bar, 2 µm. (**B**) Modulation of autophagy activity by a mutation in the *spns1* homolog (*spin-1^−/−^*; *C13C4.5*) in *C. elegans*. Representative images of autophagosomes (EGFP::LGG-1 puncta) in seam cells are shown for wild-type {*wt*, *adIs2122 [lgg-1p::GFP::lgg-1*, *rol-6(su1006)]*} animals and for nematodes carrying a homozygous *spin-1* deletion allele {*spin-1(ok2087); adIs2122 [lgg-1p::GFP::lgg-1+rol-6(su1006)*}. Arrows indicate autophagosomes only in the *spin-1^−/−^* animals. Scale bar, 5 µm. (**C**) Quantification of EGFP::LGG-1 puncta is shown for the indicated genetic backgrounds and conditions. The count of puncta per seam cell was 0.8936±0.0926 for *wt* and 1.9899±0.1396 for *spin-1(ok2087)* L4 larva, respectively [values are the mean ± standard error of mean (S.E.M.) for 94 (*wt*) and 99 [*spin-1(ok2087)*] seam cells; more than 20 animals were examined for each strain] (t-test: *p<0.0001). (**D**) The starvation sensitivity in *spin-1(ok2087)* mutant worms. Percent of worms surviving to adulthood on NGM plates with OP50 bacteria after incubation in M9 buffer in the absence of food at the L1 larval stage for the indicated times. Error bars are for standard errors of means estimated assuming a Poisson distribution, and similar results were obtained in three independent experiments. (**E**) Lifespan in *spin-1(ok2087)* mutant worms is demonstrated by Kaplan-Meier survival analysis. *spin-1(ok2087)* mutant worms are short lived compared with the wild-type N2 strain on HT115 bacteria. The median lifespan was 12 days for N2 and 10 days for *spin-1(ok2087)* (log rank test: χ^2^ = 8.834 on one degree of freedom; *p* = 0.003). Similar results were obtained in 2 experiments with 3 independent replicates each. (**F**) Shorter lifespan in heterozygous *spns1^−/+^* adult fish is demonstrated by Kaplan-Meier survival analysis (log rank test: χ^2^ = 54.05 on one degree of freedom; *p*<0.0001) and validated by Gompertz-Makeham model.
(TIF)

Figure S2 Detection of lysosomal and mitochondrial biogenesis in *spns1*-mutant animals. (**A**) Whole-mount double staining of live embryos with LysoTracker (10 µM, DND-99; red) and MitoTracker (1 µM, green) at 72 hpf. Intense LysoTracker staining was detected only in *spns1* mutants but not in *wt* animals. In contrast, MitoTracker detected equivalent signals between *wt* and *spns1*-mutant animals. Scale bar, 250 µm. (**B**) Whole-mount double staining of live embryos with MitoSox (5 µM, red) and MitoTracker (1 µM, green) at 72 hpf. Both of the probes detected equivalent signals between *wt* and *spns1*-mutant animals. Scale bar (black) in large image, 250 µm. Scale bar (white) in inset, 10 µm. (**C**) Acidity-dependent quenching of EGFP-LC3 at the LysoTracker-positive compartments in the cells from pepstatin A (5 µg/ml)- and E-64-d (5 µg/ml)-co-treated (P/E) zebrafish embryos at 72 hpf. Scale bar, 10 µm. (**D**) Acidity-dependent quenching of EGFP-LC3, but not mCherry-LC3, in cells from pepstatin A (5 µg/ml)- and E-64-d (5 µg/ml)-co-treated (P/E) zebrafish embryos at 72 hpf. Scale bar, 10 µm. (**E**) The degradation capacity of autolysosomes and lysosomes was examined by injection of a lysosomal substrate, DQ Red BSA (DQ-BSA; red) at 60 hpf. The enzyme-catalyzed hydrolysis of the intramolecular self-quenched DQ Red BSA by lysosomal proteases relieves the self-quenching, yielding brightly fluorescent reaction products. DQ Red BSA-injected *wt* control or *spns1-*mutant fish expressing EGFP-LC3 were observed at the cellular level by confocal microscopy. Scale bar, 10 µm. Quantification of data presented in E (n = 6), is shown in the right graph; the number (n) of animals is for each genotype. Three independent areas (periderm or basal epidermal cells above the eye) were selected from individual animals. Error bars represent the mean ± standard deviation (S.D.), *p<0.005.
(TIF)

Figure S3 Impact of Beclin 1 depletion on the yolk opaque phenotype and embryonic senescence in *spns1*-mutant zebrafish. (**A**) Phenotype of *beclin 1* morphant (*beclin 1* MO, 12 ng/embryo) at 24, 48 and 72 hpf. Scale bar, 250 µm. (**B**) Effect of *beclin 1* knockdown in the *spns1* mutant on the phenotypes of yolk opacity (BF; bright field) and on embryonic senescence (SABG; SA-β-gal) in the *spns1* mutant. Following injection of standard control MO or *beclin 1* MO (12 ng/embryo) into *Tg(CMV:EGFP-LC3); spns1^hi891/hi891^* embryos, SA-β-gal staining was performed to determine whether the *beclin 1* knockdown had any impact on embryonic senescence caused by Spns1 depletion at 84 hpf. Scale bar, 250 µm. Quantification of data presented in panel B (n = 12) is shown in the right graph; the number (n) of animals is for each morphant. (**C**) Parallel analyses of SA-β-gal and SA-α-fuc demonstrate the significant inductions of both activities in *spns1*-mutant animals at 84 hpf. As shown in the magnified panels, the caudal venous plexus (CVP) was the most prominently stained region. Staining for SA-β-gal was more intensive than for SA-α-fuc. Scale bar, 250 µm. Quantification of data presented in panel C (n = 12) is shown in the right graph; the number (n) of animals is for each morphant. Error bars represent the mean ± S.D., *p<0.005.
(TIF)

Figure S4 Effect of UV irradiation on *spns1*-mutant zebrafish. (**A**) Acrdine orange (green) and Lysotracker (red) intensities, as well as gross morphology, in wild-type (*wt*) and *spns1*-mutant animals treated with UV. The UV (18 mj/cm^2^) treatment was done at 36 hpf, and phenotypes were observed at 48 hpf. Scale bar, 250 µm. Quantification of data presented in A (n = 9) is shown in the right graphs; the number (n) of animals is for each genotype with or without UV treatment. (**B**) Cellular characteristics in the animals shown in (A) were observed by using confocal microscopy at high magnification (×600). Scale bar, 10 µm. Quantification of data presented in B (n = 6) is shown in the right graphs; the number (n) of animals is for each genotype with or without UV treatment. Three independent areas (periderm or basal epidermal cells above the eye) were selected from individual animals. Error bars represent the mean ± S.D., *p<0.005; ns, not significant.
(TIF)

Figure S5 Undetectable apoptosis in *spns1* and/or *beclin 1* morphants. (**A**) In *spns1* and/or *beclin 1* morphants stained with acridine orange (green) and LysoTracker (red) cellular characteristics were compared with UV-treated specimens by using confocal microscopy at high magnification (×600). Scale bar, 10 µm. Quantification of data presented in A (n = 6) is shown in the right graphs; the number (n) of animals is for each morphant and uninjected (Uninj.) animal with or without UV treatment. Three independent areas (periderm or basal epidermal cells above the eye) were selected from individual animals. (**B**) TUNEL assays demonstrate apoptosis induction in UV-treated zebrafish embryos, but not in *spns1* and/or *beclin 1* morphants. The UV (18 mj/cm^2^) treatment was done at 36 hpf, and phenotypes were observed at 48 hpf. Scale bar, 10 µm. Quantification of the fluorescence intensities is shown at the right-side graph. Quantification of data presented in B (n = 6) is shown in the right graphs; the number (n) of animals is for each morphant and uninjected (Uninj.) animal with or without UV treatment. Three independent areas (periderm or basal epidermal cells above the eye) were selected from individual animals. Error bars represent the mean ± S.D., *p<0.005.
(TIF)

Figure S6 Impact of the *beclin 1* knockdown on UV-induced apoptosis and autophagy. (**A**) Partial but significant suppression of UV-induced apoptosis in *beclin 1* morphants. The UV (18 mj/cm^2^) treatment was done at 66 hpf, followed by the phenotype observations at 72 hpf. Scale bar in the large image, 250 µm. Scale bar in the inset, 10 µm. Quantification of data presented in A (n = 9) is shown in the right graphs; the number (n) of animals is for each morphant with or without UV treatment. Three independent areas (periderm or basal epidermal cells in the caudal fin) were selected from individual animals. (**B**) Sufficient suppression of UV-induced autophagy in *beclin 1* morphants. The UV (18 mj/cm^2^) treatment was done at 69 hpf, followed by the phenotype observations at 72 hpf. Scale bar, 10 µm. Quantification of data presented in A (n = 9) is shown in the right graphs; the number (n) of animals is for each morphant with or without UV treatment. Three independent areas (periderm or basal epidermal cells above the eye) were selected from individual animals. Error bars represent the mean ± S.D., **p<0.005; *p<0.05 in (A), and *p<0.005; ns, not significant in (B).
(TIF)

Figure S7 Effects of *spns1* and *p53* knockdowns on embryonic SA-β-gal activity in *p53* and *spns1* mutants, respectively. (**A**) Effect of *spns1* knockdown on embryonic senescence in *p53* mutants. The impact of transient *spns1* knockdown on SA-β-gal induction was determined in *spns1* MO-injected *tp53^zdf1/zdf1^* animals at 72 hpf. Standard control MO was used for control injections. Scale bar, 250 µm. (**B**) Effect of *p53* knockdown on embryonic senescence in *spns1* mutants. The impact of transient *p53* knockdown on SA-β-gal induction was determined in *p53* MO-injected *spns1^hi891/hi891^* animals at 72 hpf, followed by the MO injections. Inverse *p53* MO (inv. *p53* MO) was used for control injections. Scale bar, 10 µm. (**C**) Quantification of the SA-β-gal intensities shown in (A). Quantification of data presented in panel A (n = 12) is shown in the right graph; the number (n) of animals is for each genotype with MO. (**D**) Quantification of the SA-β-gal intensities shown in (B). Quantification of data presented in panel B (n = 12) is shown in the right graph; the number (n) of animals is for each morphant in genotype. Error bars represent the mean ± S.D., *p<0.005; ns, not significant.
(TIF)

Figure S8 Impact of Beclin 1 depletion on Spns1 deficiency in the presence or absence of p53. (**A**) Yolk opaque phenotype of control MO-injected or *beclin 1* MO-injected wild-type (*spns1^+/+^;tp53^+/+^*), *tp53^zdf1/zdf1^* (*tp53^m/m^*), *spns1^hi891/hi891^* (*spns1^−/−^*), and *spns1^hi891/hi891^;tp53^zdf1/zdf1^* (*spns1^−/−^;tp53^m/m^*) animals is compared at 48 hpf. Opacity is greater in the p53 mutant background with Spns1 deficiency. The attenuated suppressive effect of *beclin 1* MO (12 ng/embryo) yolk opacity in *spns1^hi891/hi891^;tp53^zdf1/zdf1^* animals is shown. Scale bar, 250 µm. (**B**) *spns1^hi891/hi891^* animals coinjected with *beclin 1* MO and *p53* MO or *beclin 1* MO and inverse-sequence *p53* MO (inv. *p53* MO; negative control) were assayed for the SA-β-gal detection at 84 hpf. The *beclin 1* MO-mediated suppression of SA-β-gal in *spns1^hi891/hi891^* animals was attenuated by *p53* MO injection. Scale bar, 250 µm. (**C**) Quantification of the SA-β-gal intensities shown in (B). Quantification of data presented in panel B (n = 10) is shown in the right graph; the number (n) of animals is for each morphant. Error bars represent the mean ± S.D., *p<0.005; ns, not significant.
(TIF)

Figure S9 Impact of UV-induced apoptosis and autophagy on Spns1 deficiency in the presence or absence of p53. (**A**) UV-induced apoptosis can be detectable in either *spns1^+/+^* or *spns1^hi891/hi891^* animals in similar manners only under the normal p53 condition. The UV (18 mj/cm^2^) treatment was done at 60 hpf, followed by the phenotype observations in periderm or basal epidermal cells in the caudal eye at 72 hpf. Scale bar in image in top row, 250 µm. Scale bar in image in lower rows, 10 µm. (**B**) UV-induced autophagy enhances autolysosomal formation in *spns1^hi891/hi891^* animals in the presence of p53. The UV (18 mj/cm^2^) treatment was done at 69 hpf, followed by the phenotype observations in periderm or basal epidermal cells in the caudal fin at 72 hpf. Scale bar, 10 µm. (**C**) Quantification of the EGFP-LC3 and LysoTracker fluorescence intensities shown in (B). Quantification of data presented in panel B (n = 6) is shown in the right graph; the number (n) of animals is for each genotype with MO. Three independent areas (periderm or basal epidermal cells in the caudal fin) were selected from individual animals. Error bars represent the mean ± S.D., *p<0.005; ns, not significant.
(TIF)

Figure S10 Detection of DNA damage response and DNA synthesis in *spns1* mutants in the presence or absence of p53. (**A**) γH2AX- and BrdU detection in *spns1* mutants in p53- and DNA damage-dependent manners. As shown in the green fluorescent panels, unaltered γH2AX intensities between *spns1^+/+^* and *spns1^hi891/hi891^* (*spns1^−/−^*) were apparent irrespective of p53 status without UV irradiation. Increased γH2AX intensities in response to UV irradiation were observed in the presence of p53 regardless of Spns1 status. Of note, certain basal increases of γH2AX intensities were detected in the *p53* mutant background. As shown in the red fluorescent panels, reduced BrdU incorporation in *spns1^hi891/hi891^* animals was detected in either normal or mutant p53 condition in the absence of UV treatment. UV-induced inhibition of DNA synthesis (reduction of BrdU signals) is apparently seen only in the normal p53 situation. The UV (18 mj/cm^2^) treatment was done at 68 hpf, followed by the phenotype observations at 72 hpf. Scale bar in the large image, 250 µm. Scale bar in the small merged image and inset, 10 µm. (**B**) Quantification of the γH2AX fluorescence intensities shown in (A). Quantification of data presented in panel A (n = 12) is shown in the right graph; the number (n) of animals is for each genotype. Three independent areas (periderm or basal epidermal cells in the trunk) were selected from individual animals. (**C**) Quantification of the BrdU-positive cells [in 25.6±2.2×10^4^ µm areas; the trunk region starting from the rostral start point of the yolk extension (the distal end of the yolk) through the end of the caudal fin] shown in (A). Error bars represent the mean ± S.D., **p<0.005; *p<0.05; ns, not significant.
(TIF)

Figure S11 Detection of mitotic cells in *spns1* mutants in the presence or absence of p53. (**A**) Phosphorylated histone H3 (pH 3) staining in *spns1*-mutant animals with normal or mutant p53 backgrounds. The UV (18 mj/cm^2^) treatment was done at 68 hpf, followed by the phenotype observations at 72 hpf. Scale bar, 250 µm. (**B**) Quantification of the pH 3-positive cells [in 27.2±3.2×10^4^ µm areas; the trunk region starting from the rostral start point of the yolk extension (the distal end of yolk) through the end of the caudal fin] shown in (A). Quantification of data presented in panel A (n = 9) is shown in the right graph; the number (n) of animals is for each genotype. Three independent areas (periderm or basal epidermal cells in the trunk) were selected from individual animals. Reduction of the pH 3 level was statistically significant in *spns1^hi891/hi891^* (*spns1^−/−^*) animals in the presence of p53, and a reduced tendency (with no statistical significance) was also observed in *spns1* mutants. Error bars represent the mean ± S.D., **p<0.05; *p<0.01; ns, not significant.
(TIF)

Figure S12 Impact of UV irradiation on embryonic SA-β-gal activity in *p53* and/or *spns1* mutants. (**A**) Effect of UV treatment on embryonic SA-β-gal activity was validated in *spns1*-mutant animals with normal or mutant p53 backgrounds. The UV (18 mj/cm^2^) treatment was done at 68 hpf, followed by the phenotype observations at 72 hpf. Scale bar, 250 µm. (**B**) Quantification of the SA-β-gal intensities shown in (A). Quantification of data presented in panel A (n = 12) is shown in the right graph; the number (n) of animals is for each genotype. Error bars represent the mean ± S.D., **p<0.05; *p<0.01; ns, not significant.
(TIF)

Figure S13 Semi-quantitative RT-PCR analyses of the expression of *p21*, *pai-1*, *smp-30*, *and bax* genes in *spns1* and/or *p53* mutants at 72 hpf. (**A**) A representative gel-loading pattern of each gene expression. (**B**) Quantification of the gene expression shown in (A). Data are mean ± SD [n = 6 samples (3 embryos/sample) per genotype]. Asterisks denote significant changes compared to *wt* values. *p<0.05.
(TIF)

Figure S14 Gene-expression profiles of potential markers and/or mediators of senescence in *spns1*-defective zebrafish embryos. (**A**) Semi-quantitative RT-PCR analyses of senescence markers and/or mediators and of p53-downstream target genes in *spns1* and/or *beclin 1* morphants. The expression was detected at 72 hpf. Data are mean ± SD [n = 4 samples (3 embryos/sample) per morphant]. Asterisks denote significant changes from standard control MO injected values. *p<0.05. (**B**) Semi-quantitative RT-PCR analyses of senescence marker and/or mediator expression as well as p53-downstream target genes in *spns1* and/or *p53* mutants with or without UV treatment. The UV (18 mj/cm^2^) treatment was done at 66 hpf, and the expression was detected at 72 hpf. Data are mean ± SD [n = 6 samples (3 embryos/sample) per genotype]. Asterisks denote significant changes between values. *p<0.05.
(TIF)

Figure S15 Semi-quantitative RT-PCR analyses of *ink4ab* gene expression in *spns1* and/or *p53* mutants with or without UV treatment. The UV (18 mj/cm^2^) treatment was done at 66 hpf, and the expression was detected at 72 hpf. Data are mean ± SD [n = 6 samples (3 embryos/sample) per genotype]. Asterisks denote significant changes from *p53^+/+^;spns1^+/+^* without UV treatment (-) values. *p<0.05.
(TIF)

Figure S16 Suppression of *spns1*-mutant phenotypes by BafA treatment in zebrafish embryos. Suppression of yolk opacity by treatment with BafA (200 nM; 12 h treatment from 48 hpf through 60 hpf) in pigmented (AB line) and unpigmented (*casper* line) zebrafish embryos is shown. Scale bar, 250 µm.
(TIF)

Figure S17 Suppression of *spns1*-mutant phenotypes by knockdown of the *atp6v0c* gene in zebrafish embryos. (**A**) Gross morphology, EGFP-LC3 and LysoTracker intensities in wild-type (*wt*) and *spns1*-mutant animals injected with *atp6v0c* MO (4 ng/embryo) at 48 hpf. Suppression of yolk opacity and SA-β-gal (SABG) by injection of *atp6v0c* MO in zebrafish embryos was observed at 48 and 60 hpf, respectively. Scale bar, 250 µm. (**B**) Quantification of the SA-β-gal intensities shown in (A). Quantification of data presented in panel A (n = 10) is shown in the right graph; the number (n) of animals is for each genotype with MO. (**C**) Effect of the PPIs (omeprazole; OPZ, lansoprazole; LPZ, and pantoprazole; PPZ) on embryonic senescence (SABG; SA-β-gal) in the *spns1* mutant at 48 hpf. The drug treatments were done for 12 h from 36 hpf through 48 hpf. Scale bar, 250 µm. Error bars represent the mean ± S.D., *p<0.005; ns, not significant.
(TIF)

Figure S18 Validations of lysosomal biogenesis and acidity in zebrafish embryos. (**A**) Whole-mount double staining with LysoTracker (10 µM, DND-99; red) and LysoSensor 189 (1 µM, DND-189; green). Live animals at 72 hpf were counterstained with LysoTracker and acidic pH-sensitive LysoSensor 189, simultaneously. LysoSensor 189 weakly detects acidic lysosomal signals in the *spns1*-mutant animals. Scale bar, 250 µm. Quantification of data presented for LysoSensor 189 (green) and LysoTracker (red) signals in panel A (n = 12) is shown in the right graph; the number (n) of animals is for each genotype. (**B**) Whole-mount double staining with LysoTracker (10 µM, DND-99; red) and LysoSensor 153 (1 µM, DND-153; green). Animals at 72 hpf were simultaneously counterstained by LysoTracker and neutral pH-sensitive LysoSensor 153. LysoSensor 153 can detect relatively neutral lysosomal signals in the *spns1*-mutant animals. Scale bar, 250 µm. Quantification of data presented for LysoSensor 153 (green) and LysoTracker (red) signals in panel B (n = 12) is shown in the right graph; the number (n) of animals is for each genotype. (**C**) Acidic pH-sensitive LysoSensor 189 (1 µM, green) probe in combination with LysoTracker (10 µM, red) was used in *wt* and *spns1*-mutant animals, and detectable signals in cells were obtained at 72 hpf. In *wt* fish treated with pepstatin A and E-64-d (P/E) (5 µg/ml each for 12 h), autolysosomal and/or lysosomal compartments were more prominently detected by LysoSensor 189 at the cellular level with enhanced accumulation of enlarged compartments under the identical LysoTracker staining condition. In contrast, in *spns1*-mutant animals, the cellular compartments were only weakly detectable by LysoSensor 189. Importantly, the short-term BafA treatment (for 1 h) largely attenuated or abolished staining of acidic compartments by both LysoSensor and LysoTracker, indicating that these autolysosomal and lysosomal compartments in *wt* animals treated with pepstatin A and E-64-d may retain some strong (lower pH) acidity. Scale bar, 10 µm. Quantification of data presented for LysoSensor 189 (green) and LysoTracker (red) signals in panel C (n = 12) is shown; the number (n) of animals is for each genotype with DMSO, pepstatin A and E-64-d (P/E) and/or BafA (+BafA; 1 h treatment). Three independent areas (periderm or basal epidermal cells above the eye) were selected from individual animals. (**D**) Using neutral pH-sensitive LysoSensor 153 (green) probes in combination with LysoTracker (red), *wt* and *spns1*-mutant animals were examined for detectable signals in cells when stained at 72 hpf. In *spns1*-mutant animals, autolysosomal and/or lysosomal compartments were more prominently detected by LysoSensor 153 at the cellular level with enhanced accumulation of enlarged compartments. In stark contrast, the cellular compartments in *wt* fish treated with pepstatin A and E-64-d (P/E) (5 µg/ml each for 12 h) were less detectable by LysoSensor 153 under the same staining condition used with LysoTracker. The short-term BafA treatment (for 1 h) still abolished the acidic compartments stained by both LysoSensor and LysoTracker, suggesting that the autolysosomal and lysosomal compartments observed in *spns1*-mutants may still retain some weak (higher pH) acidity. Scale bar, 10 µm. Quantification of data presented for LysoSensor 153 (green) and LysoTracker (red) signals in panel D (n = 12) is shown; the number (n) of animals is for each genotype with DMSO, pepstatin A and E-64-d (P/E) and/or BafA (+BafA; 1 h treatment). Three independent areas (periderm or basal epidermal cells above the eye) were selected from individual animals. Error bars represent the mean ± S.D., *p<0.005; ns, not significant in (A), (B) and (D), and **p<0.005; *p<0.05; ns, not significant in (C).
(TIF)

Figure S19 Validations of autolysosome formation and lysosomal biogenesis in zebrafish embryos. (**A**) Gross morphologies of BafA (100 nM)-treated or pepstatin A (5 µg/ml)- and E-64-d (5 µg/ml)-co-treated (P/E) *wt* [*Tg(CMV:EGFP-LC3*)] and *spns1*-mutant [*Tg(CMV:EGFP-LC3); spns1^hi891/hi891^*] animals. Embryos at 60 hpf were incubated with BafA or P/E for 12 h, and stained with LysoTracker at 72 hpf. Scale bar, 250 µm. Quantification of data presented in the middle and bottom rows (green; EGFP, red; mCherry) in panel A (n = 12) is shown; the number (n) of animals is for each genotype with DMSO, BafA or pepstatin A and E-64-d (P/E). (**B**) Intracellular autolysosome formation and lysosomal biogenesis in vehicle (DMSO)-treated, BafA (100 nM)-treated or pepstatin A (5 µg/ml)- and E-64-d (5 µg/ml)-treated (P/E) *wt* [*Tg(CMV:EGFP-LC3*)] and *spns1*-mutant [*Tg(CMV:EGFP-LC3);spns1^hi891/hi891^*] animals. Numerous large EGFP-LC3 puncta are evident in the BafA-treated embryos, with minimal LysoTracker staining. Some increased EGFP-LC3 speckles and strong enhancement of enlarged LysoTracker signals are evident in the cells from P/E-treated embryos. The same samples analyzed in (A) were observed by using confocal microscopy at a high magnification (×600). Scale bar, 10 µm. Quantification of data presented in the middle and bottom rows (green; EGFP, red; mCherry) in panel A (n = 6) is shown. The number (n) of animals is for each genotype with DMSO, BafA or pepstatin A and E-64-d (P/E). Three independent areas (periderm or basal epidermal cells above the eye) were selected from individual animals. (**C**) Impaired autolysosomal acidification in BafA-treated *wt* or in *spns1*-mutant embryos, but not in pepstatin A- and E-64-d-treated (P/E) *wt* embryos. EGFP-LC3 and mCherry-LC3 double-transgenic *wt* [*Tg(CMV:EGFP-LC3:mCherry-LC3*)] and *spns1*-mutant [*Tg(CMV:EGFP-LC3:mCherry-LC3);spns1^hi891/hi891^*] zebrafish were used to monitor autolysosome formation. Embryos at 60 hpf were incubated with BafA (100 nM) or P/E (5 µg/ml each) for 12 h, to be observed later at 72 hpf. Quenching of EGFP-LC3 signals but not mCherry-LC3 signals is seen in the P/E-treated embryos, whereas unquenched EGFP-LC3 signals are evident in the BafA-treated as well as the *spns1* MO-injected embryos. Whole-mount samples were observed by using confocal microscopy at a high magnification (×600). Scale bar, 10 µm. Quantification of data presented in the middle and bottom rows (green; EGFP, red; mCherry) in panel A (n = 6) is shown; the number (n) of animals is for each genotype with DMSO, BafA or pepstatin A and E-64-d (P/E). Three independent areas (periderm or basal epidermal cells above the eye) were selected from individual animals.
(TIF)

Table S1 List of primers used for qPCR.
(DOC)

Table S2 List of primers used for RT-PCR and list of PCR conditions.
(DOC)

Text S1 Supplemental data.
(DOC)

Text S2 Supplemental Materials and Methods.
(DOC)
