# Supplementary figures and images for: Aberrant Autolysosomal Regulation Is Linked to The Induction of Embryonic Senescence: Differential Roles of Beclin 1 and p53 in Vertebrate Spns1 Deficiency
Source: PLoS Genet. 2014 Jun 26;10(6):e1004409. doi: 10.1371/journal.pgen.1004409 (PMC4072523; doi:10.1371/journal.pgen.1004409)

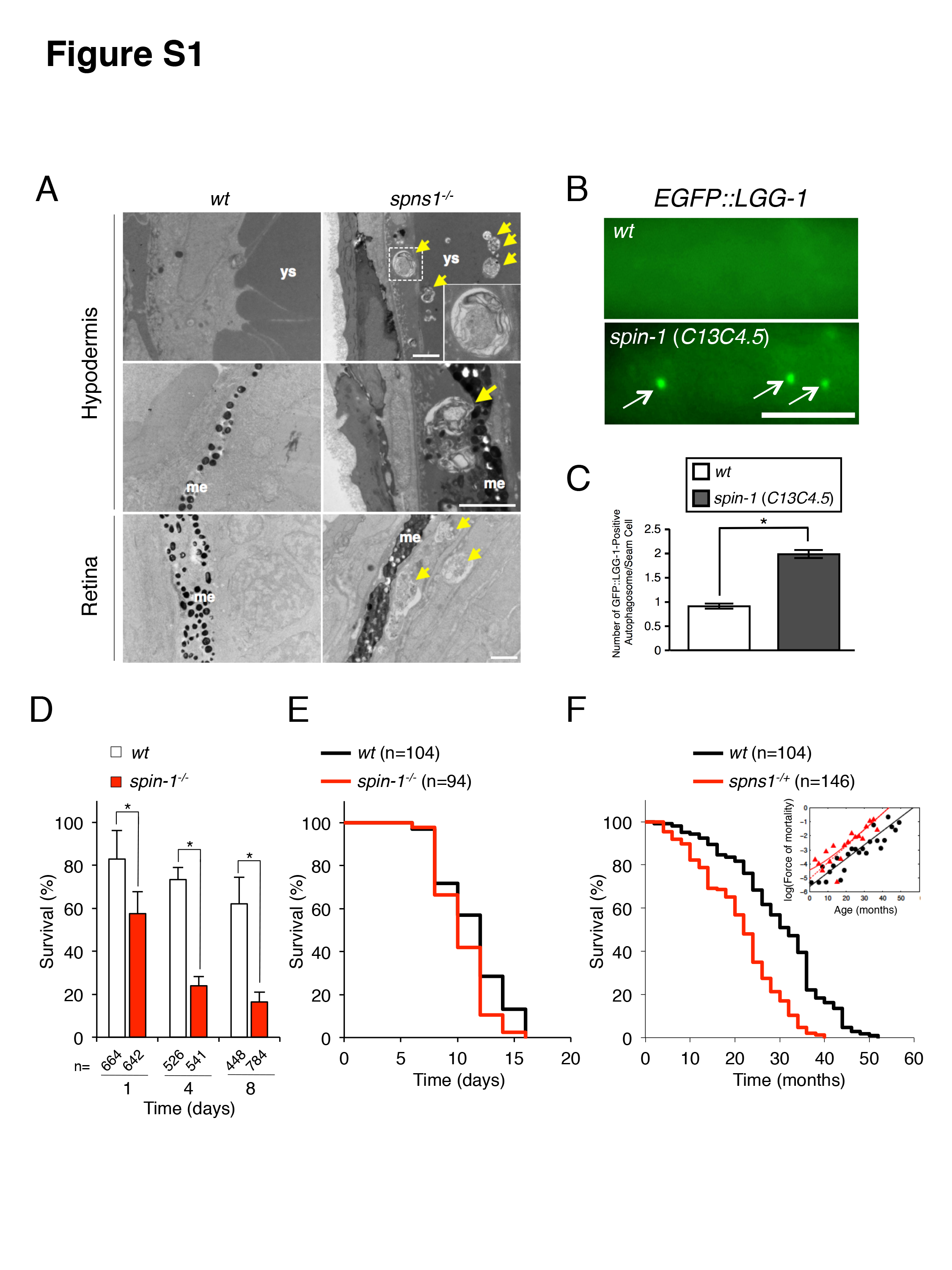

Supplement: Figure S1 — Autophagic abnormalities and survival in spns1-mutant fish and worms. (A) Representative transmission electron microscopy images of normal wt or spns1-mutant fish larvae at 84 hpf. Compared with wild-type (wt) control (left), the spns1 mutant (spns1−/−) (right) accumulates abnormal cytoplasmic inclusions at the hypodermal regions adjacent to yolk sac (ys) (upper two panels) or melanophores (me) (middle two panels), and in the retinal pigment epithelium containing melanophores (me) (lower two panels). Arrows indicate cytoplasmic membranous inclusions. In the right-upper panel, the inset shows a magnified image of the cytoplasmic inclusion surrounded with a dotted square. Scale bar, 2 µm. (B) Modulation of autophagy activity by a mutation in the spns1 homolog (spin-1−/−; C13C4.5) in C. elegans. Representative images of autophagosomes (EGFP::LGG-1 puncta) in seam cells are shown for wild-type {wt, adIs2122 [lgg-1p::GFP::lgg-1, rol-6(su1006)]} animals and for nematodes carrying a homozygous spin-1 deletion allele {spin-1(ok2087); adIs2122 [lgg-1p::GFP::lgg-1+rol-6(su1006)}. Arrows indicate autophagosomes only in the spin-1−/− animals. Scale bar, 5 µm. (C) Quantification of EGFP::LGG-1 puncta is shown for the indicated genetic backgrounds and conditions. The count of puncta per seam cell was 0.8936±0.0926 for wt and 1.9899±0.1396 for spin-1(ok2087) L4 larva, respectively [values are the mean ± standard error of mean (S.E.M.) for 94 (wt) and 99 [spin-1(ok2087)] seam cells; more than 20 animals were examined for each strain] (t-test: *p<0.0001). (D) The starvation sensitivity in spin-1(ok2087) mutant worms. Percent of worms surviving to adulthood on NGM plates with OP50 bacteria after incubation in M9 buffer in the absence of food at the L1 larval stage for the indicated times. Error bars are for standard errors of means estimated assuming a Poisson distribution, and similar results were obtained in three independent experiments. (E) Lifespan in spin-1(ok2087) mutant worms [file pgen.1004409.s001.tif]

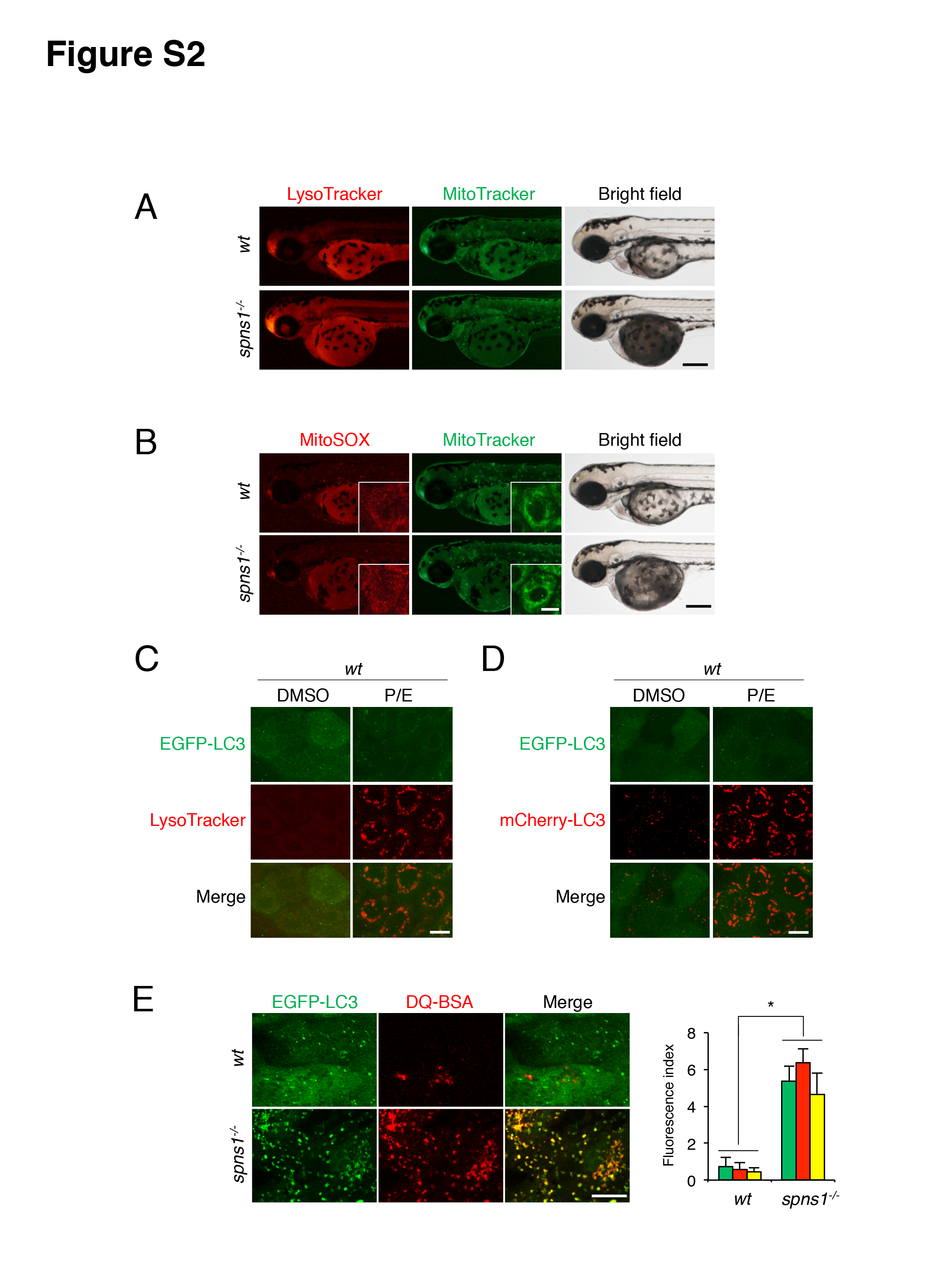

Supplement: Figure S2 — Detection of lysosomal and mitochondrial biogenesis in spns1-mutant animals. (A) Whole-mount double staining of live embryos with LysoTracker (10 µM, DND-99; red) and MitoTracker (1 µM, green) at 72 hpf. Intense LysoTracker staining was detected only in spns1 mutants but not in wt animals. In contrast, MitoTracker detected equivalent signals between wt and spns1-mutant animals. Scale bar, 250 µm. (B) Whole-mount double staining of live embryos with MitoSox (5 µM, red) and MitoTracker (1 µM, green) at 72 hpf. Both of the probes detected equivalent signals between wt and spns1-mutant animals. Scale bar (black) in large image, 250 µm. Scale bar (white) in inset, 10 µm. (C) Acidity-dependent quenching of EGFP-LC3 at the LysoTracker-positive compartments in the cells from pepstatin A (5 µg/ml)- and E-64-d (5 µg/ml)-co-treated (P/E) zebrafish embryos at 72 hpf. Scale bar, 10 µm. (D) Acidity-dependent quenching of EGFP-LC3, but not mCherry-LC3, in cells from pepstatin A (5 µg/ml)- and E-64-d (5 µg/ml)-co-treated (P/E) zebrafish embryos at 72 hpf. Scale bar, 10 µm. (E) The degradation capacity of autolysosomes and lysosomes was examined by injection of a lysosomal substrate, DQ Red BSA (DQ-BSA; red) at 60 hpf. The enzyme-catalyzed hydrolysis of the intramolecular self-quenched DQ Red BSA by lysosomal proteases relieves the self-quenching, yielding brightly fluorescent reaction products. DQ Red BSA-injected wt control or spns1-mutant fish expressing EGFP-LC3 were observed at the cellular level by confocal microscopy. Scale bar, 10 µm. Quantification of data presented in E (n = 6), is shown in the right graph; the number (n) of animals is for each genotype. Three independent areas (periderm or basal epidermal cells above the eye) were selected from individual animals. Error bars represent the mean ± standard deviation (S.D.), *p<0.005. (TIF) [file pgen.1004409.s002.tif]

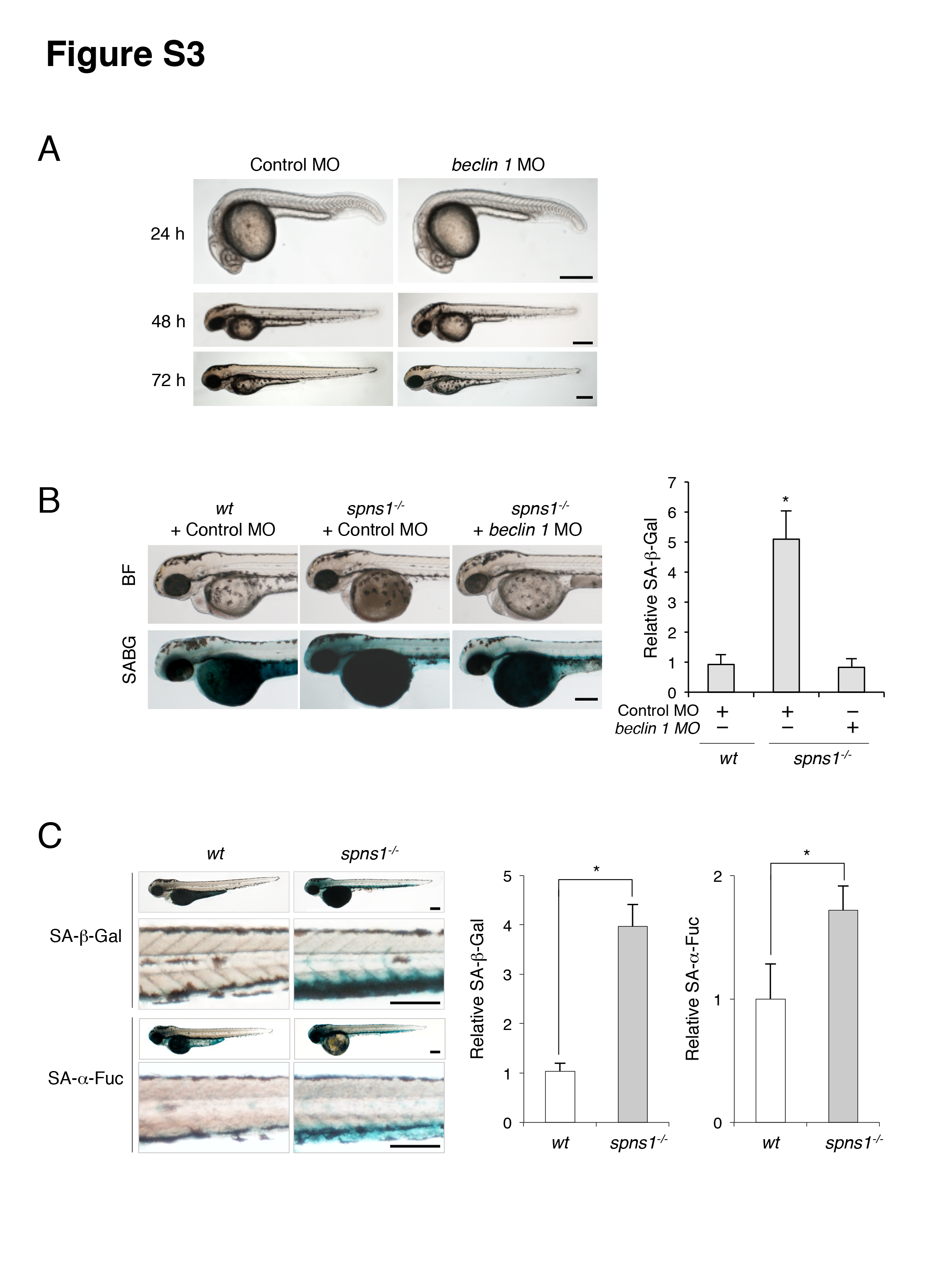

Supplement: Figure S3 — Impact of Beclin 1 depletion on the yolk opaque phenotype and embryonic senescence in spns1-mutant zebrafish. (A) Phenotype of beclin 1 morphant (beclin 1 MO, 12 ng/embryo) at 24, 48 and 72 hpf. Scale bar, 250 µm. (B) Effect of beclin 1 knockdown in the spns1 mutant on the phenotypes of yolk opacity (BF; bright field) and on embryonic senescence (SABG; SA-β-gal) in the spns1 mutant. Following injection of standard control MO or beclin 1 MO (12 ng/embryo) into Tg(CMV:EGFP-LC3); spns1hi891/hi891 embryos, SA-β-gal staining was performed to determine whether the beclin 1 knockdown had any impact on embryonic senescence caused by Spns1 depletion at 84 hpf. Scale bar, 250 µm. Quantification of data presented in panel B (n = 12) is shown in the right graph; the number (n) of animals is for each morphant. (C) Parallel analyses of SA-β-gal and SA-α-fuc demonstrate the significant inductions of both activities in spns1-mutant animals at 84 hpf. As shown in the magnified panels, the caudal venous plexus (CVP) was the most prominently stained region. Staining for SA-β-gal was more intensive than for SA-α-fuc. Scale bar, 250 µm. Quantification of data presented in panel C (n = 12) is shown in the right graph; the number (n) of animals is for each morphant. Error bars represent the mean ± S.D., *p<0.005. (TIF) [file pgen.1004409.s003.tif]

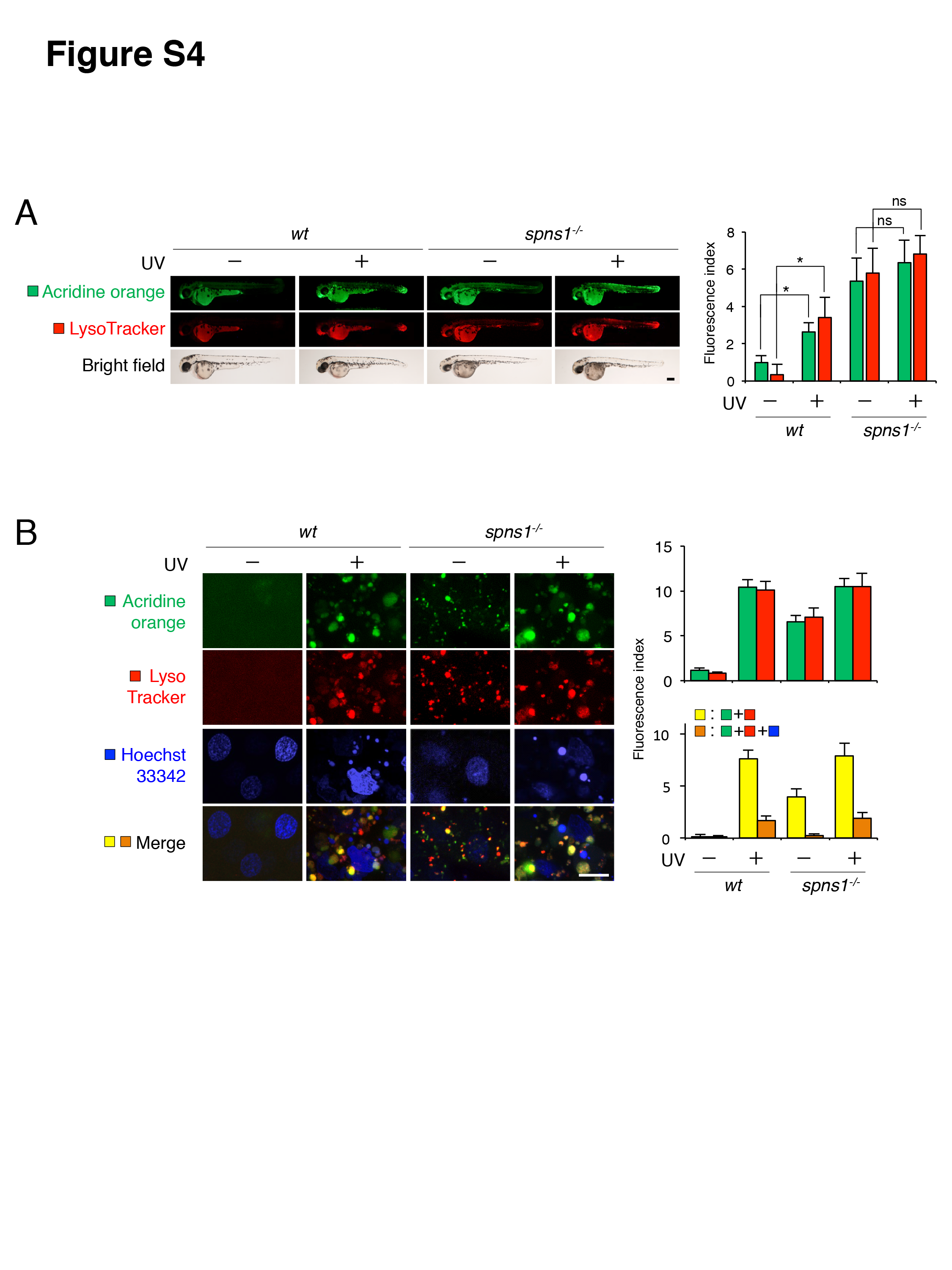

Supplement: Figure S4 — Effect of UV irradiation on spns1-mutant zebrafish. (A) Acrdine orange (green) and Lysotracker (red) intensities, as well as gross morphology, in wild-type (wt) and spns1-mutant animals treated with UV. The UV (18 mj/cm2) treatment was done at 36 hpf, and phenotypes were observed at 48 hpf. Scale bar, 250 µm. Quantification of data presented in A (n = 9) is shown in the right graphs; the number (n) of animals is for each genotype with or without UV treatment. (B) Cellular characteristics in the animals shown in (A) were observed by using confocal microscopy at high magnification (×600). Scale bar, 10 µm. Quantification of data presented in B (n = 6) is shown in the right graphs; the number (n) of animals is for each genotype with or without UV treatment. Three independent areas (periderm or basal epidermal cells above the eye) were selected from individual animals. Error bars represent the mean ± S.D., *p<0.005; ns, not significant. (TIF) [file pgen.1004409.s004.tif]

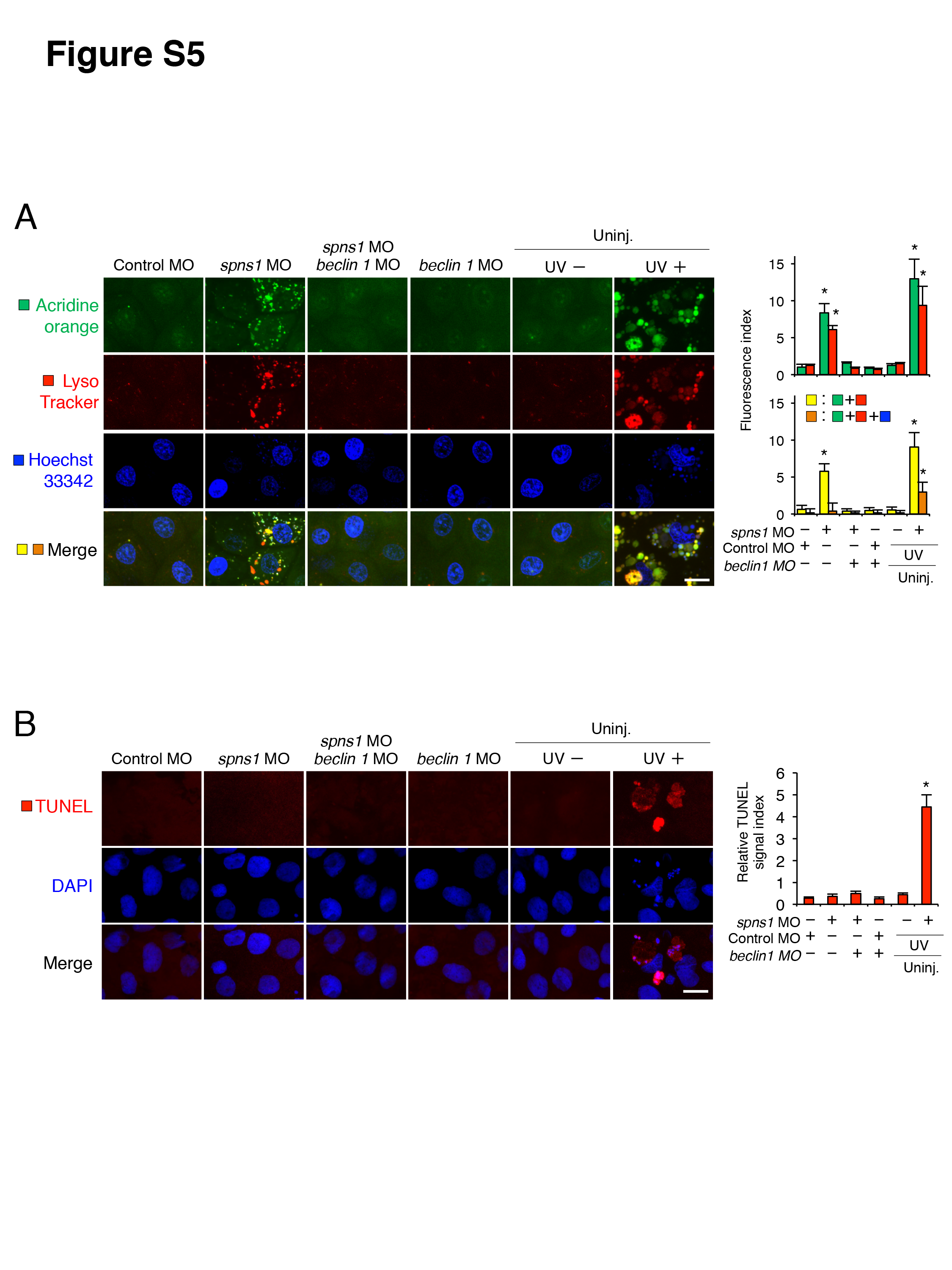

Supplement: Figure S5 — Undetectable apoptosis in spns1 and/or beclin 1 morphants. (A) In spns1 and/or beclin 1 morphants stained with acridine orange (green) and LysoTracker (red) cellular characteristics were compared with UV-treated specimens by using confocal microscopy at high magnification (×600). Scale bar, 10 µm. Quantification of data presented in A (n = 6) is shown in the right graphs; the number (n) of animals is for each morphant and uninjected (Uninj.) animal with or without UV treatment. Three independent areas (periderm or basal epidermal cells above the eye) were selected from individual animals. (B) TUNEL assays demonstrate apoptosis induction in UV-treated zebrafish embryos, but not in spns1 and/or beclin 1 morphants. The UV (18 mj/cm2) treatment was done at 36 hpf, and phenotypes were observed at 48 hpf. Scale bar, 10 µm. Quantification of the fluorescence intensities is shown at the right-side graph. Quantification of data presented in B (n = 6) is shown in the right graphs; the number (n) of animals is for each morphant and uninjected (Uninj.) animal with or without UV treatment. Three independent areas (periderm or basal epidermal cells above the eye) were selected from individual animals. Error bars represent the mean ± S.D., *p<0.005. (TIF) [file pgen.1004409.s005.tif]

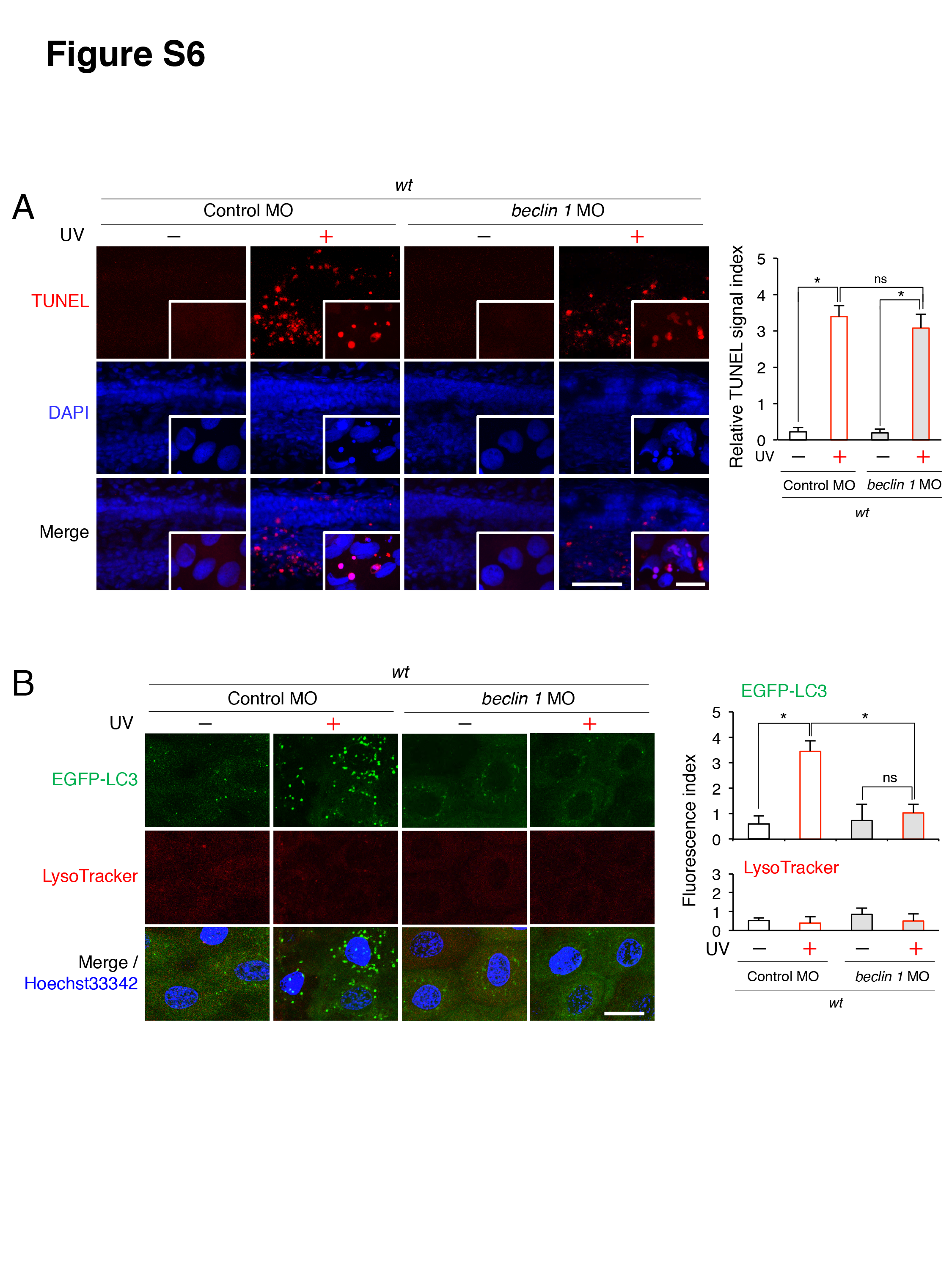

Supplement: Figure S6 — Impact of the beclin 1 knockdown on UV-induced apoptosis and autophagy. (A) Partial but significant suppression of UV-induced apoptosis in beclin 1 morphants. The UV (18 mj/cm2) treatment was done at 66 hpf, followed by the phenotype observations at 72 hpf. Scale bar in the large image, 250 µm. Scale bar in the inset, 10 µm. Quantification of data presented in A (n = 9) is shown in the right graphs; the number (n) of animals is for each morphant with or without UV treatment. Three independent areas (periderm or basal epidermal cells in the caudal fin) were selected from individual animals. (B) Sufficient suppression of UV-induced autophagy in beclin 1 morphants. The UV (18 mj/cm2) treatment was done at 69 hpf, followed by the phenotype observations at 72 hpf. Scale bar, 10 µm. Quantification of data presented in A (n = 9) is shown in the right graphs; the number (n) of animals is for each morphant with or without UV treatment. Three independent areas (periderm or basal epidermal cells above the eye) were selected from individual animals. Error bars represent the mean ± S.D., **p<0.005; *p<0.05 in (A), and *p<0.005; ns, not significant in (B). (TIF) [file pgen.1004409.s006.tif]

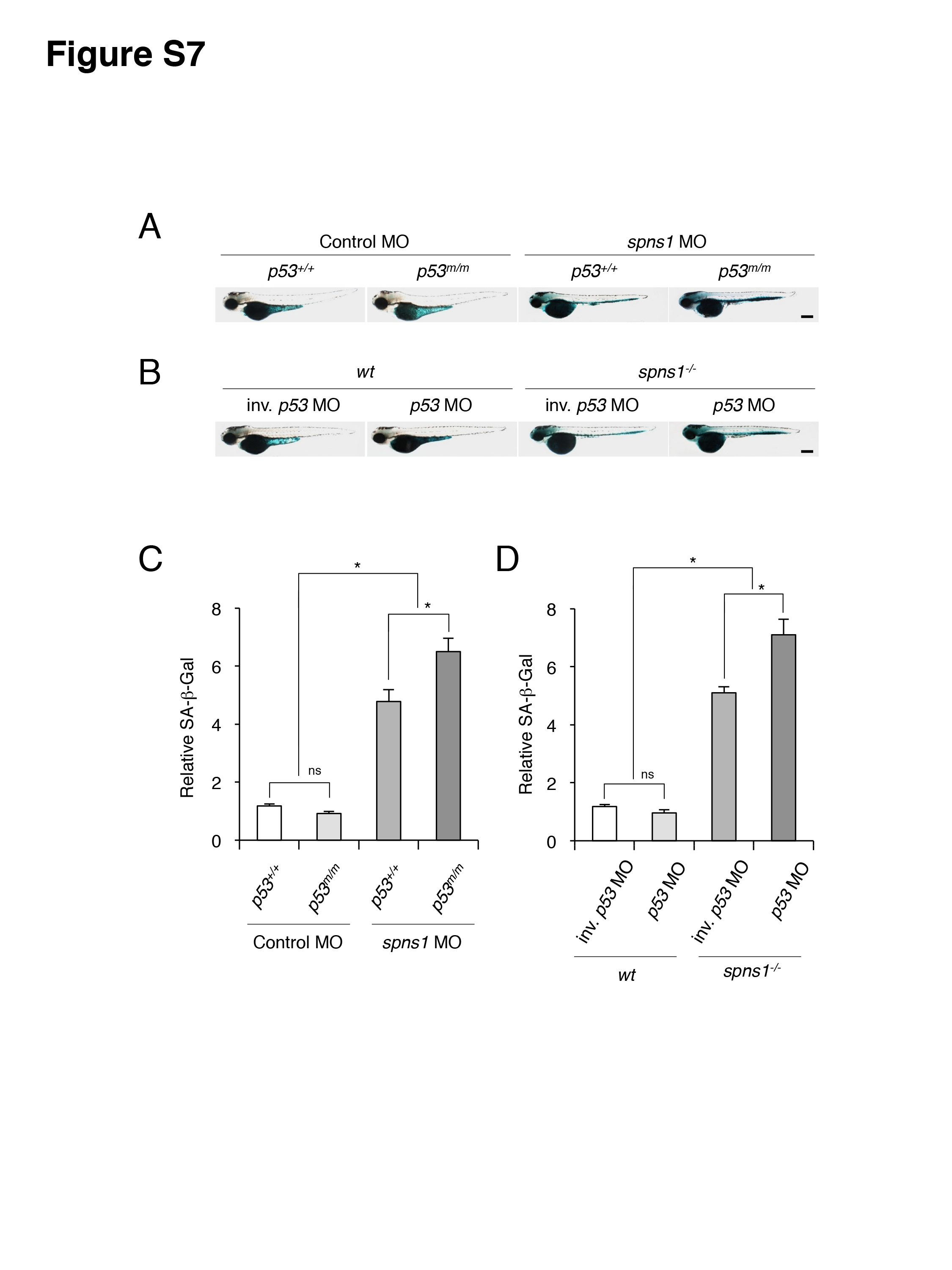

Supplement: Figure S7 — Effects of spns1 and p53 knockdowns on embryonic SA-β-gal activity in p53 and spns1 mutants, respectively. (A) Effect of spns1 knockdown on embryonic senescence in p53 mutants. The impact of transient spns1 knockdown on SA-β-gal induction was determined in spns1 MO-injected tp53zdf1/zdf1 animals at 72 hpf. Standard control MO was used for control injections. Scale bar, 250 µm. (B) Effect of p53 knockdown on embryonic senescence in spns1 mutants. The impact of transient p53 knockdown on SA-β-gal induction was determined in p53 MO-injected spns1hi891/hi891 animals at 72 hpf, followed by the MO injections. Inverse p53 MO (inv. p53 MO) was used for control injections. Scale bar, 10 µm. (C) Quantification of the SA-β-gal intensities shown in (A). Quantification of data presented in panel A (n = 12) is shown in the right graph; the number (n) of animals is for each genotype with MO. (D) Quantification of the SA-β-gal intensities shown in (B). Quantification of data presented in panel B (n = 12) is shown in the right graph; the number (n) of animals is for each morphant in genotype. Error bars represent the mean ± S.D., *p<0.005; ns, not significant. (TIF) [file pgen.1004409.s007.tif]

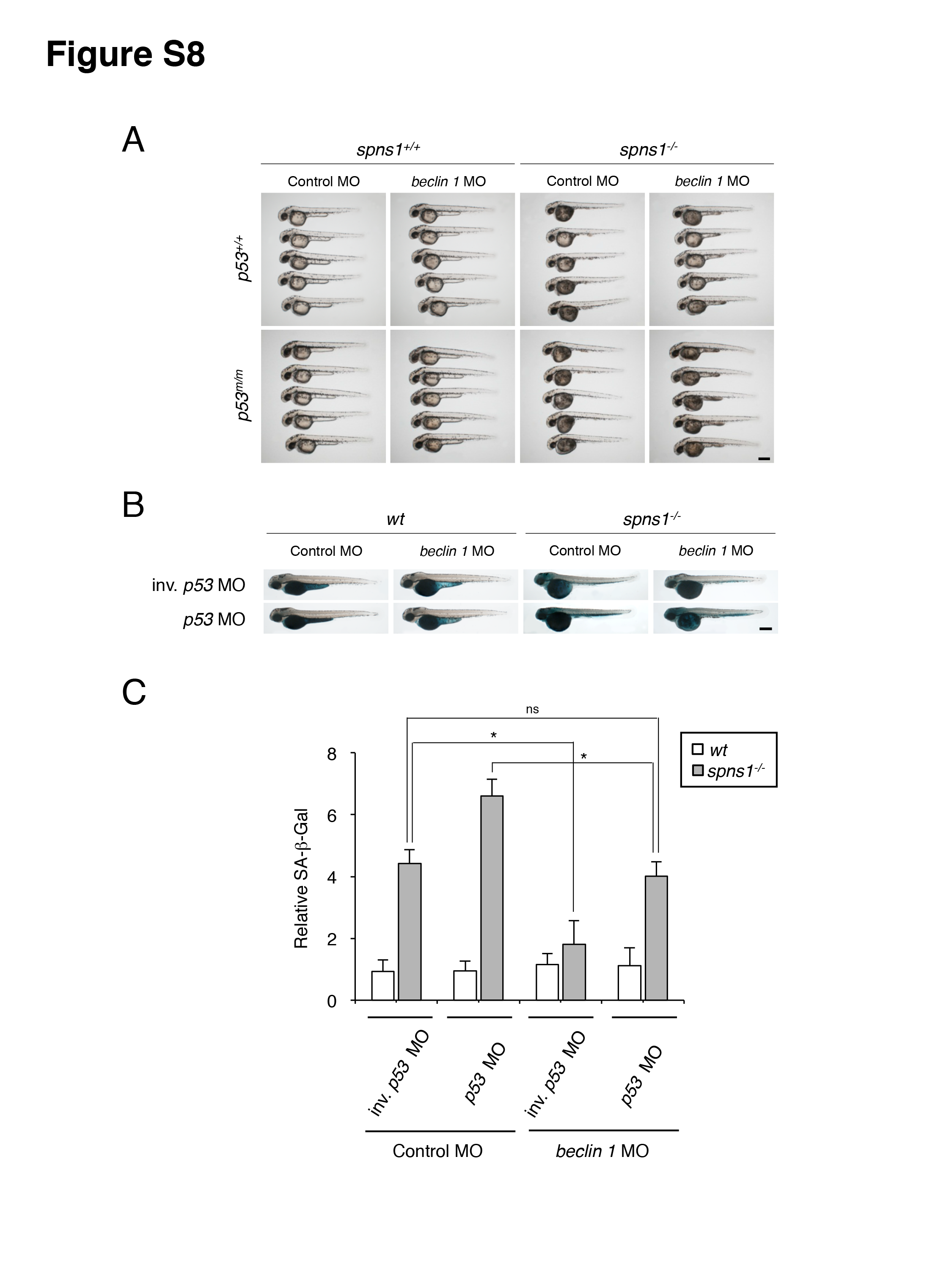

Supplement: Figure S8 — Impact of Beclin 1 depletion on Spns1 deficiency in the presence or absence of p53. (A) Yolk opaque phenotype of control MO-injected or beclin 1 MO-injected wild-type (spns1+/+;tp53+/+), tp53zdf1/zdf1 (tp53m/m), spns1hi891/hi891 (spns1−/−), and spns1hi891/hi891;tp53zdf1/zdf1 (spns1−/−;tp53m/m) animals is compared at 48 hpf. Opacity is greater in the p53 mutant background with Spns1 deficiency. The attenuated suppressive effect of beclin 1 MO (12 ng/embryo) yolk opacity in spns1hi891/hi891;tp53zdf1/zdf1 animals is shown. Scale bar, 250 µm. (B) spns1hi891/hi891 animals coinjected with beclin 1 MO and p53 MO or beclin 1 MO and inverse-sequence p53 MO (inv. p53 MO; negative control) were assayed for the SA-β-gal detection at 84 hpf. The beclin 1 MO-mediated suppression of SA-β-gal in spns1hi891/hi891 animals was attenuated by p53 MO injection. Scale bar, 250 µm. (C) Quantification of the SA-β-gal intensities shown in (B). Quantification of data presented in panel B (n = 10) is shown in the right graph; the number (n) of animals is for each morphant. Error bars represent the mean ± S.D., *p<0.005; ns, not significant. (TIF) [file pgen.1004409.s008.tif]

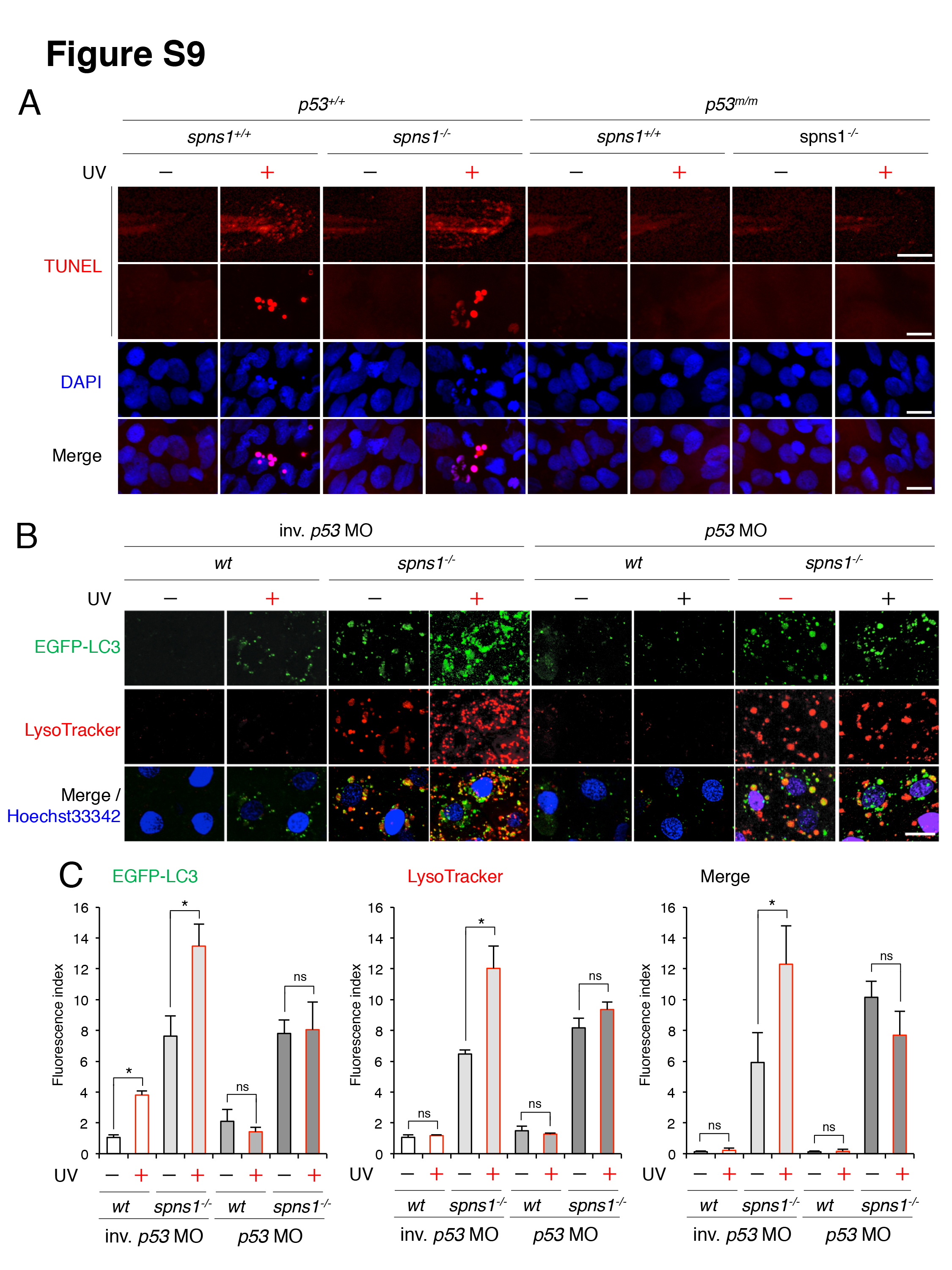

Supplement: Figure S9 — Impact of UV-induced apoptosis and autophagy on Spns1 deficiency in the presence or absence of p53. (A) UV-induced apoptosis can be detectable in either spns1+/+ or spns1hi891/hi891 animals in similar manners only under the normal p53 condition. The UV (18 mj/cm2) treatment was done at 60 hpf, followed by the phenotype observations in periderm or basal epidermal cells in the caudal eye at 72 hpf. Scale bar in image in top row, 250 µm. Scale bar in image in lower rows, 10 µm. (B) UV-induced autophagy enhances autolysosomal formation in spns1hi891/hi891 animals in the presence of p53. The UV (18 mj/cm2) treatment was done at 69 hpf, followed by the phenotype observations in periderm or basal epidermal cells in the caudal fin at 72 hpf. Scale bar, 10 µm. (C) Quantification of the EGFP-LC3 and LysoTracker fluorescence intensities shown in (B). Quantification of data presented in panel B (n = 6) is shown in the right graph; the number (n) of animals is for each genotype with MO. Three independent areas (periderm or basal epidermal cells in the caudal fin) were selected from individual animals. Error bars represent the mean ± S.D., *p<0.005; ns, not significant. (TIF) [file pgen.1004409.s009.tif]

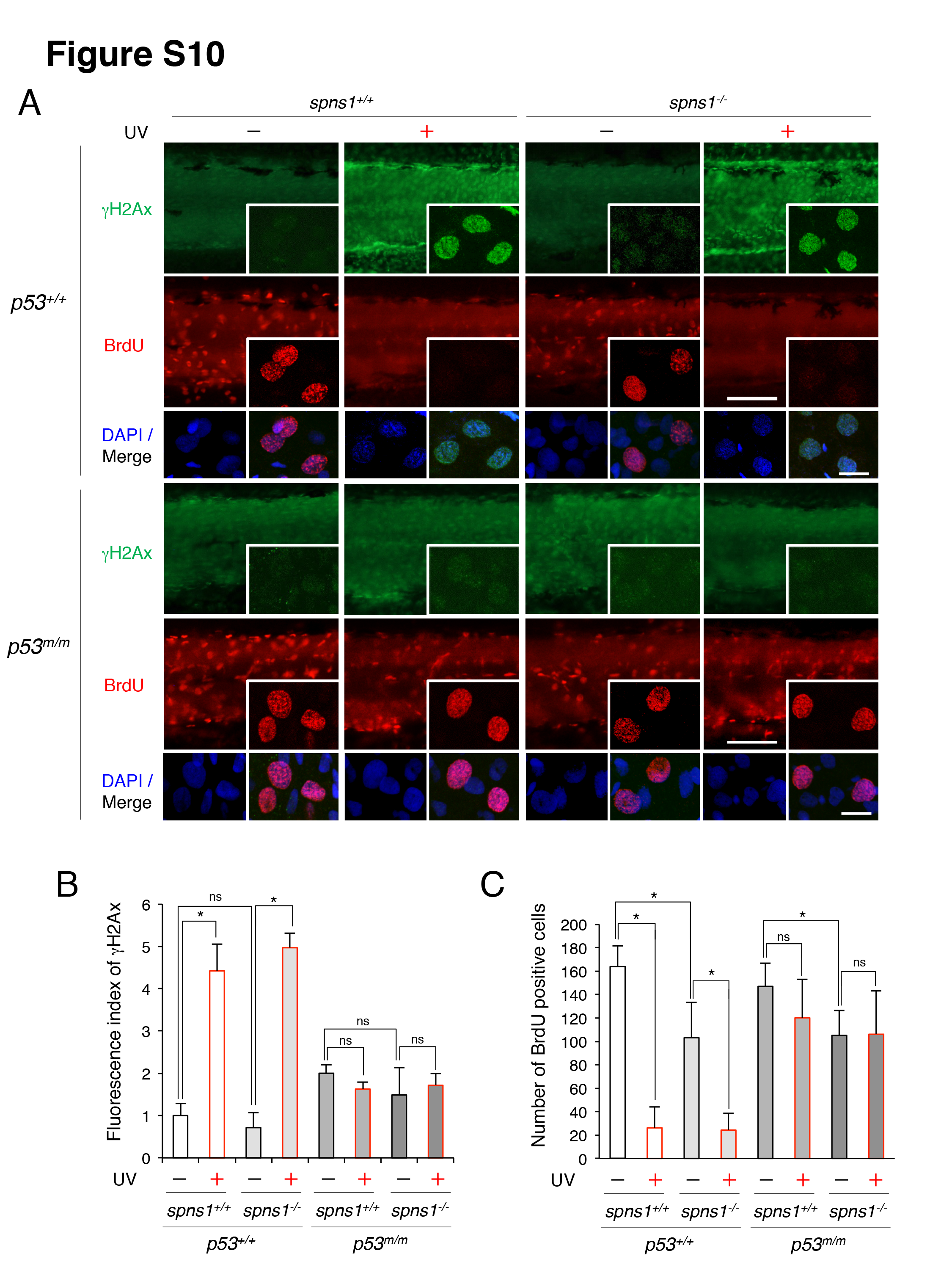

Supplement: Figure S10 — Detection of DNA damage response and DNA synthesis in spns1 mutants in the presence or absence of p53. (A) γH2AX- and BrdU detection in spns1 mutants in p53- and DNA damage-dependent manners. As shown in the green fluorescent panels, unaltered γH2AX intensities between spns1+/+ and spns1hi891/hi891 (spns1−/−) were apparent irrespective of p53 status without UV irradiation. Increased γH2AX intensities in response to UV irradiation were observed in the presence of p53 regardless of Spns1 status. Of note, certain basal increases of γH2AX intensities were detected in the p53 mutant background. As shown in the red fluorescent panels, reduced BrdU incorporation in spns1hi891/hi891 animals was detected in either normal or mutant p53 condition in the absence of UV treatment. UV-induced inhibition of DNA synthesis (reduction of BrdU signals) is apparently seen only in the normal p53 situation. The UV (18 mj/cm2) treatment was done at 68 hpf, followed by the phenotype observations at 72 hpf. Scale bar in the large image, 250 µm. Scale bar in the small merged image and inset, 10 µm. (B) Quantification of the γH2AX fluorescence intensities shown in (A). Quantification of data presented in panel A (n = 12) is shown in the right graph; the number (n) of animals is for each genotype. Three independent areas (periderm or basal epidermal cells in the trunk) were selected from individual animals. (C) Quantification of the BrdU-positive cells [in 25.6±2.2×104 µm areas; the trunk region starting from the rostral start point of the yolk extension (the distal end of the yolk) through the end of the caudal fin] shown in (A). Error bars represent the mean ± S.D., **p<0.005; *p<0.05; ns, not significant. (TIF) [file pgen.1004409.s010.tif]

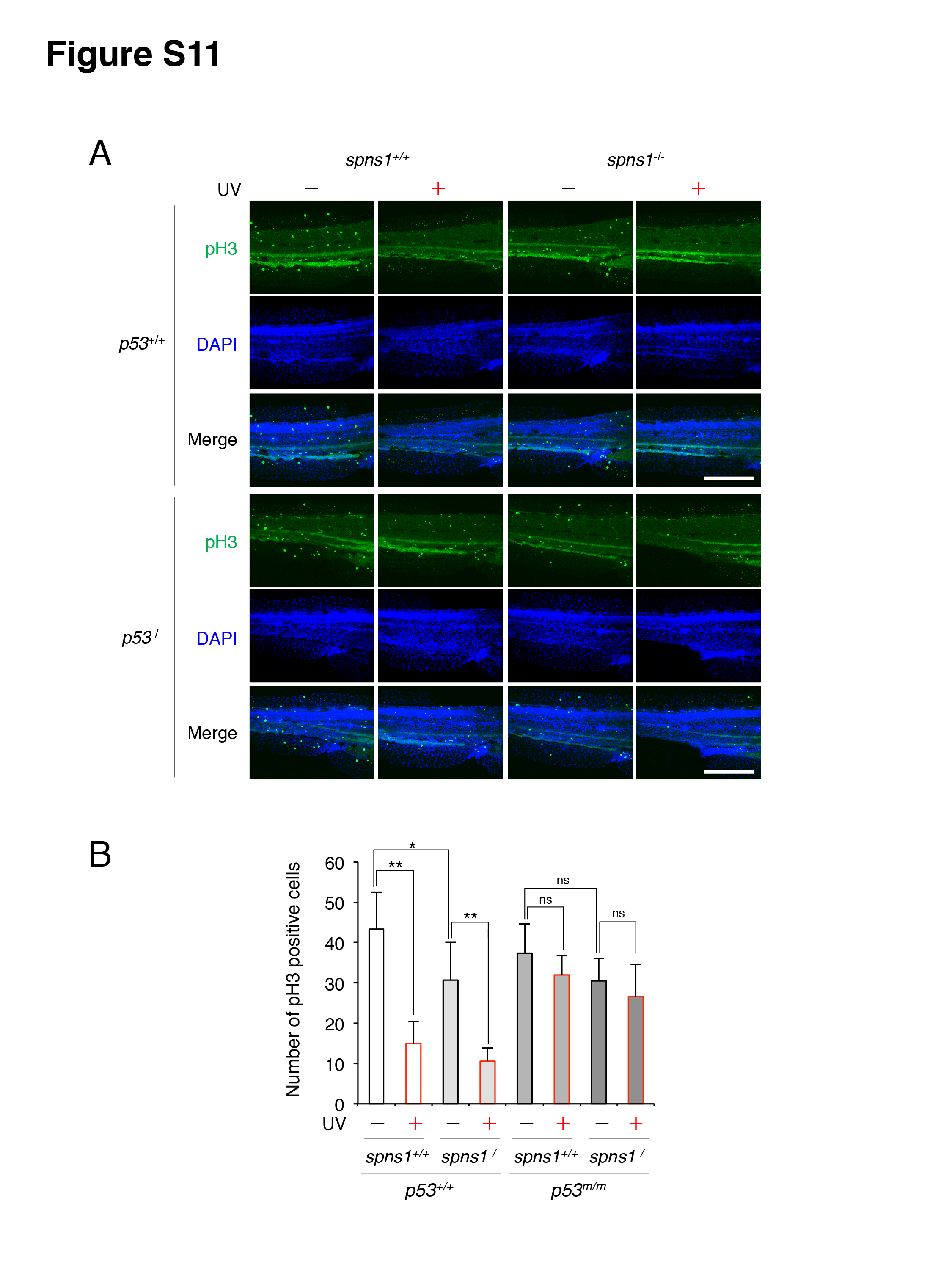

Supplement: Figure S11 — Detection of mitotic cells in spns1 mutants in the presence or absence of p53. (A) Phosphorylated histone H3 (pH 3) staining in spns1-mutant animals with normal or mutant p53 backgrounds. The UV (18 mj/cm2) treatment was done at 68 hpf, followed by the phenotype observations at 72 hpf. Scale bar, 250 µm. (B) Quantification of the pH 3-positive cells [in 27.2±3.2×104 µm areas; the trunk region starting from the rostral start point of the yolk extension (the distal end of yolk) through the end of the caudal fin] shown in (A). Quantification of data presented in panel A (n = 9) is shown in the right graph; the number (n) of animals is for each genotype. Three independent areas (periderm or basal epidermal cells in the trunk) were selected from individual animals. Reduction of the pH 3 level was statistically significant in spns1hi891/hi891 (spns1−/−) animals in the presence of p53, and a reduced tendency (with no statistical significance) was also observed in spns1 mutants. Error bars represent the mean ± S.D., **p<0.05; *p<0.01; ns, not significant. (TIF) [file pgen.1004409.s011.tif]

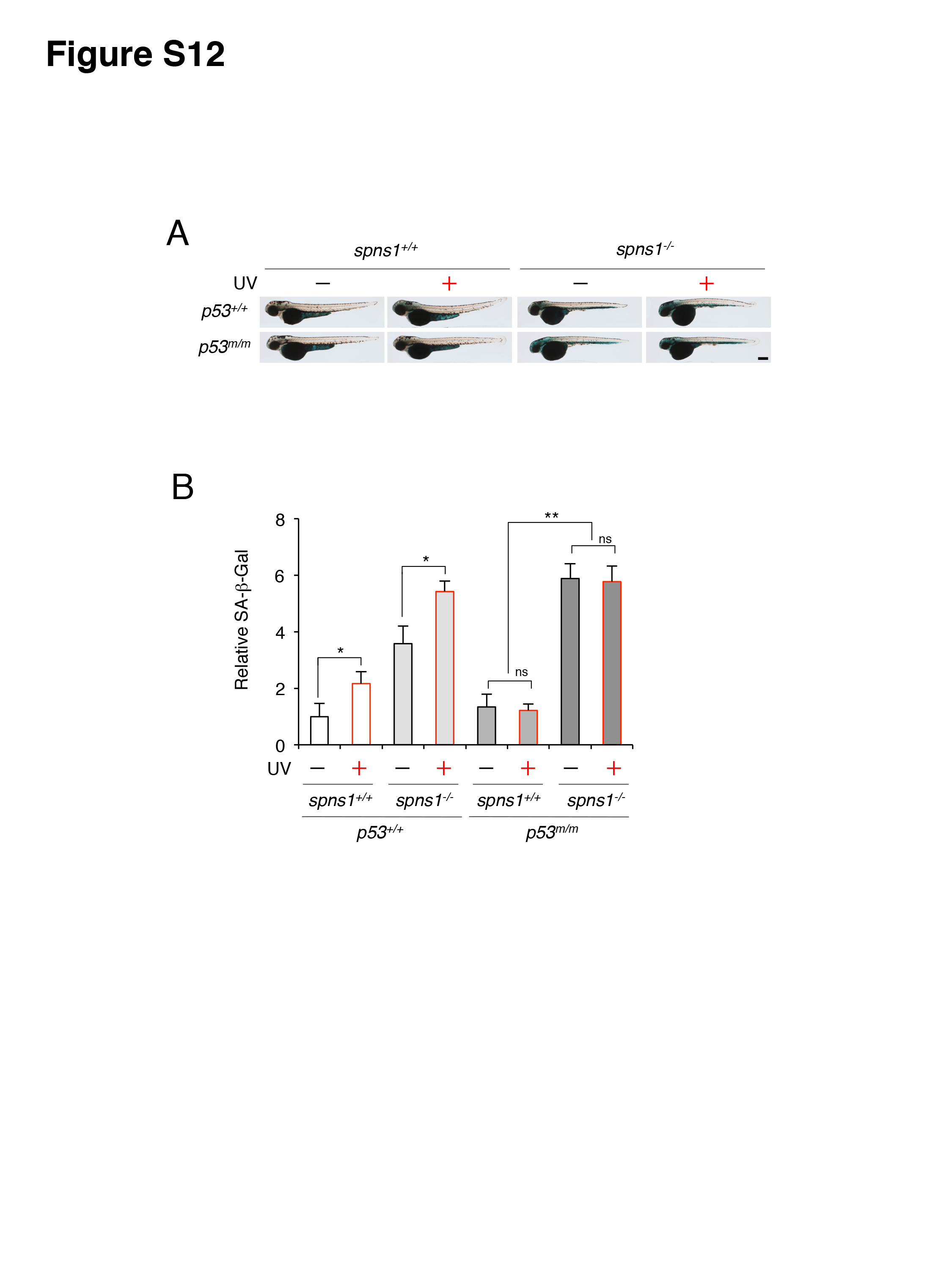

Supplement: Figure S12 — Impact of UV irradiation on embryonic SA-β-gal activity in p53 and/or spns1 mutants. (A) Effect of UV treatment on embryonic SA-β-gal activity was validated in spns1-mutant animals with normal or mutant p53 backgrounds. The UV (18 mj/cm2) treatment was done at 68 hpf, followed by the phenotype observations at 72 hpf. Scale bar, 250 µm. (B) Quantification of the SA-β-gal intensities shown in (A). Quantification of data presented in panel A (n = 12) is shown in the right graph; the number (n) of animals is for each genotype. Error bars represent the mean ± S.D., **p<0.05; *p<0.01; ns, not significant. (TIF) [file pgen.1004409.s012.tif]

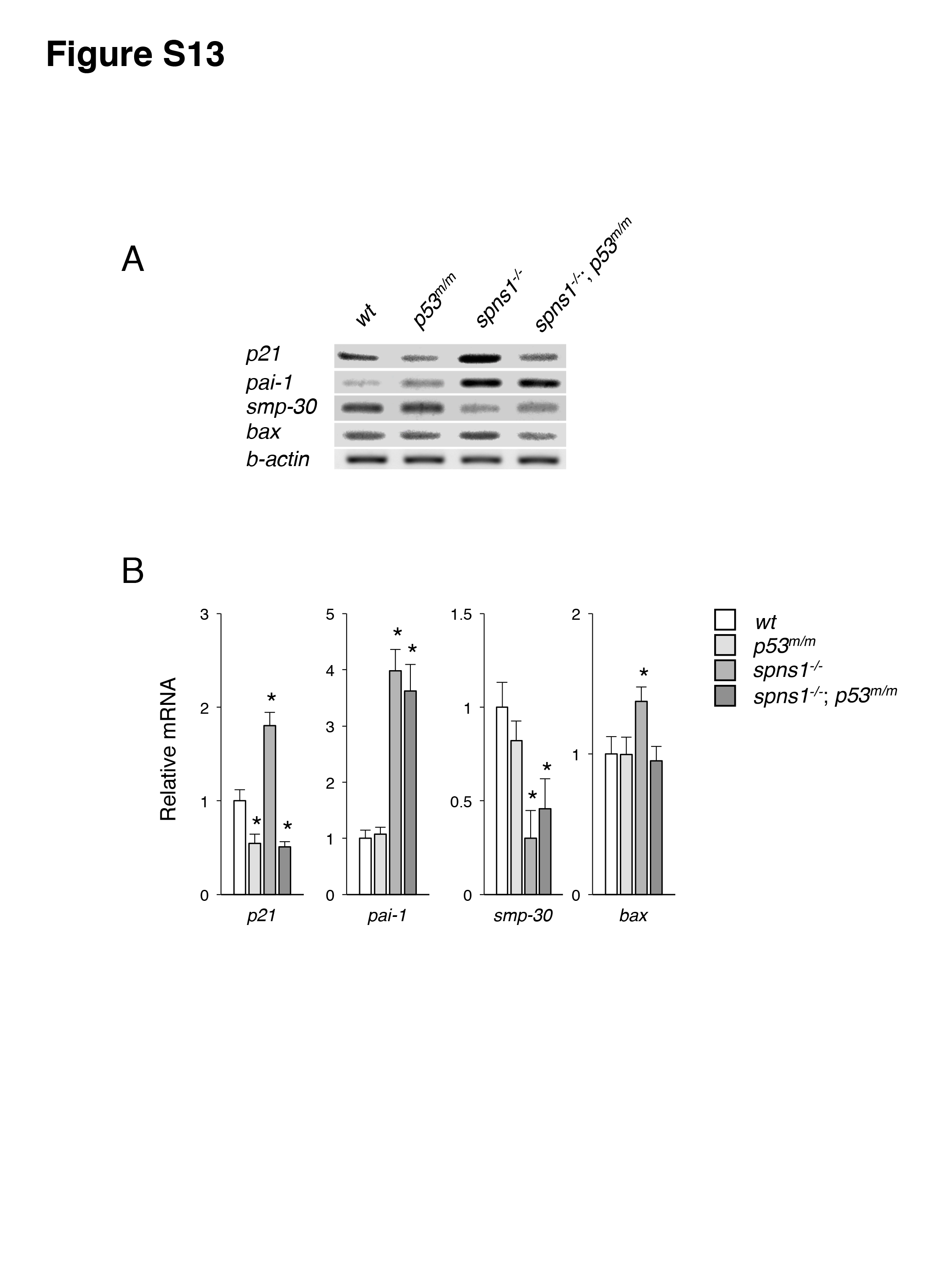

Supplement: Figure S13 — Semi-quantitative RT-PCR analyses of the expression of p21, pai-1, smp-30, and bax genes in spns1 and/or p53 mutants at 72 hpf. (A) A representative gel-loading pattern of each gene expression. (B) Quantification of the gene expression shown in (A). Data are mean ± SD [n = 6 samples (3 embryos/sample) per genotype]. Asterisks denote significant changes compared to wt values. *p<0.05. (TIF) [file pgen.1004409.s013.tif]

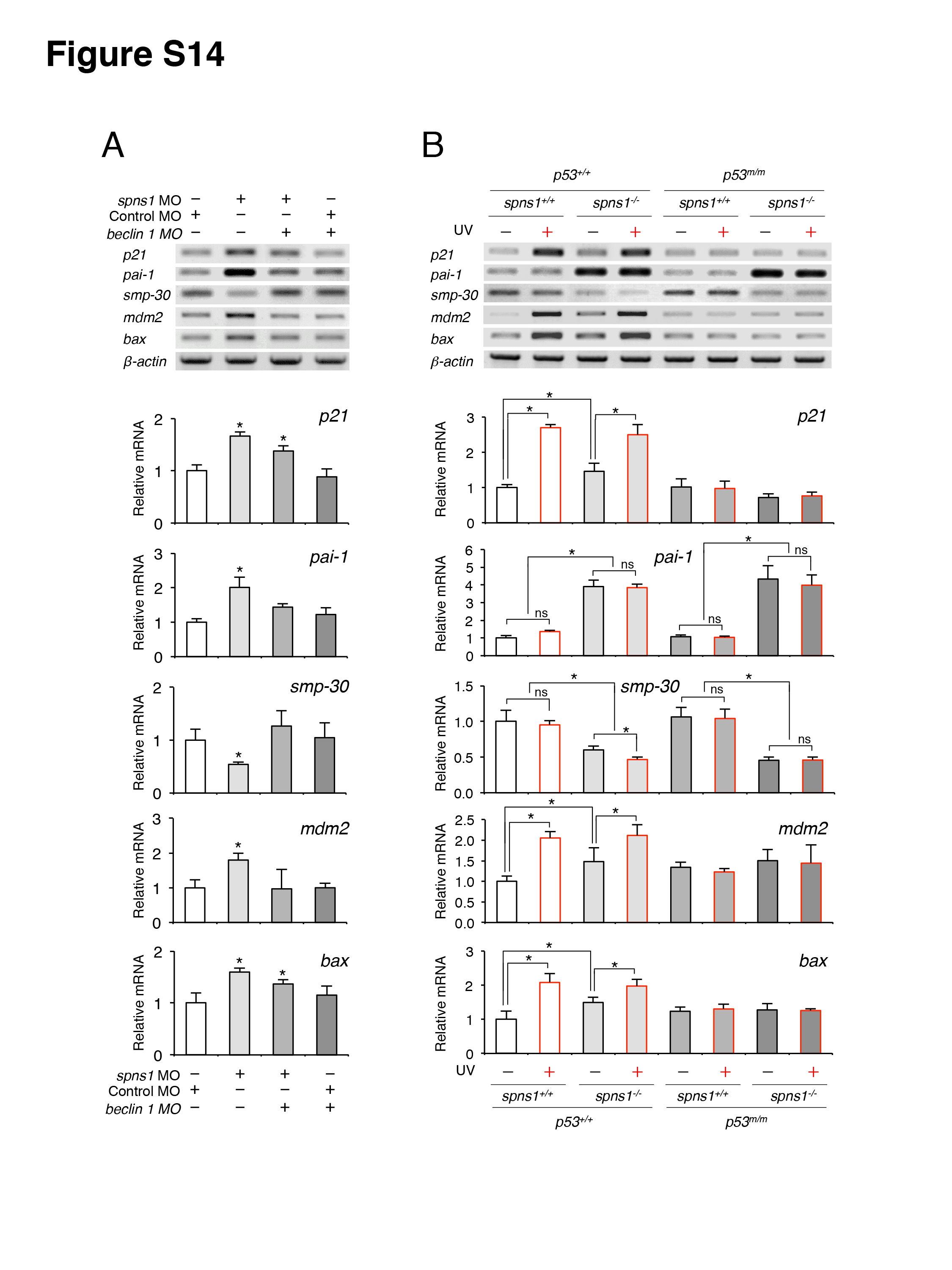

Supplement: Figure S14 — Gene-expression profiles of potential markers and/or mediators of senescence in spns1-defective zebrafish embryos. (A) Semi-quantitative RT-PCR analyses of senescence markers and/or mediators and of p53-downstream target genes in spns1 and/or beclin 1 morphants. The expression was detected at 72 hpf. Data are mean ± SD [n = 4 samples (3 embryos/sample) per morphant]. Asterisks denote significant changes from standard control MO injected values. *p<0.05. (B) Semi-quantitative RT-PCR analyses of senescence marker and/or mediator expression as well as p53-downstream target genes in spns1 and/or p53 mutants with or without UV treatment. The UV (18 mj/cm2) treatment was done at 66 hpf, and the expression was detected at 72 hpf. Data are mean ± SD [n = 6 samples (3 embryos/sample) per genotype]. Asterisks denote significant changes between values. *p<0.05. (TIF) [file pgen.1004409.s014.tif]

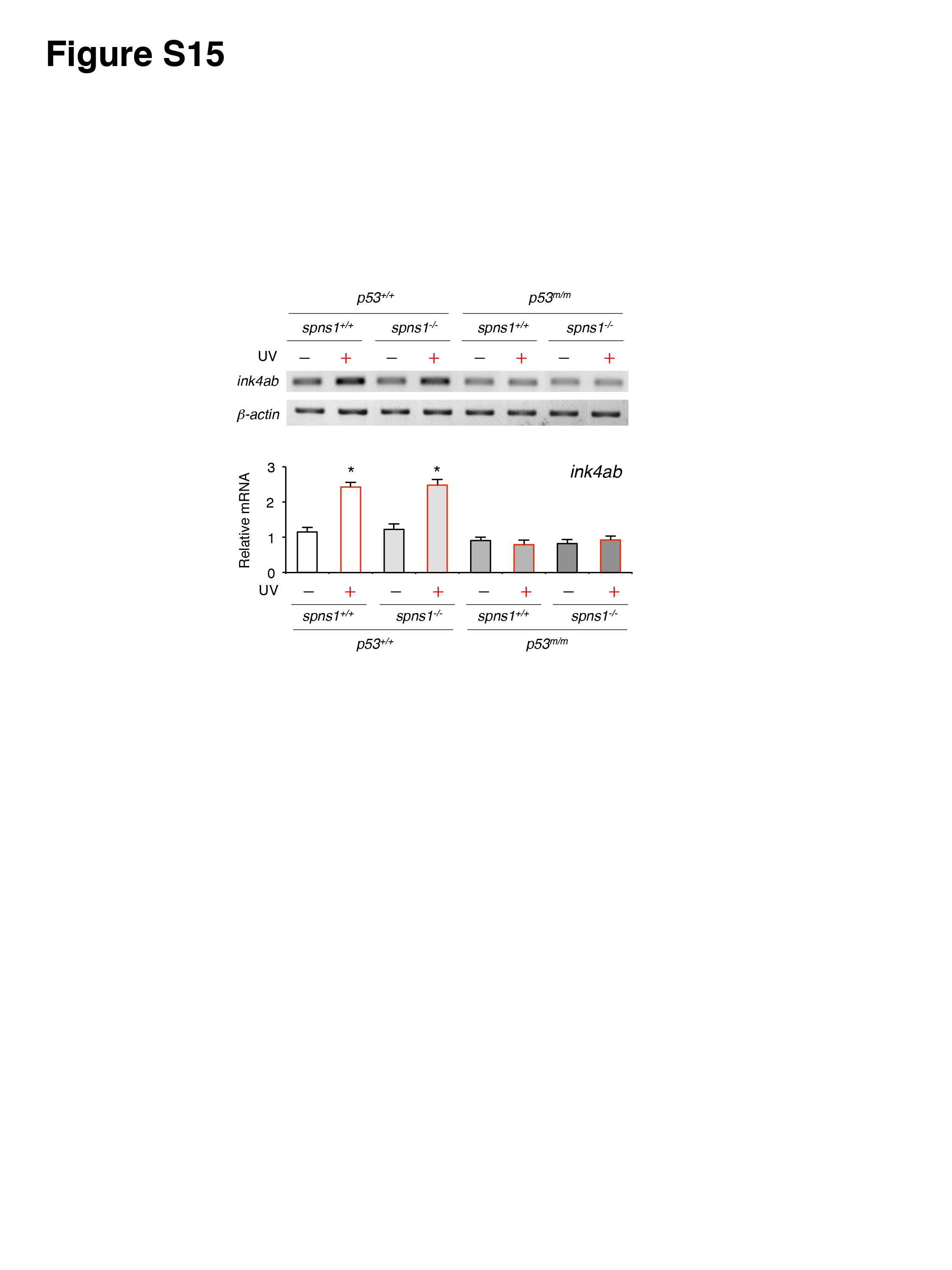

Supplement: Figure S15 — Semi-quantitative RT-PCR analyses of ink4ab gene expression in spns1 and/or p53 mutants with or without UV treatment. The UV (18 mj/cm2) treatment was done at 66 hpf, and the expression was detected at 72 hpf. Data are mean ± SD [n = 6 samples (3 embryos/sample) per genotype]. Asterisks denote significant changes from p53+/+;spns1+/+ without UV treatment (-) values. *p<0.05. (TIF) [file pgen.1004409.s015.tif]

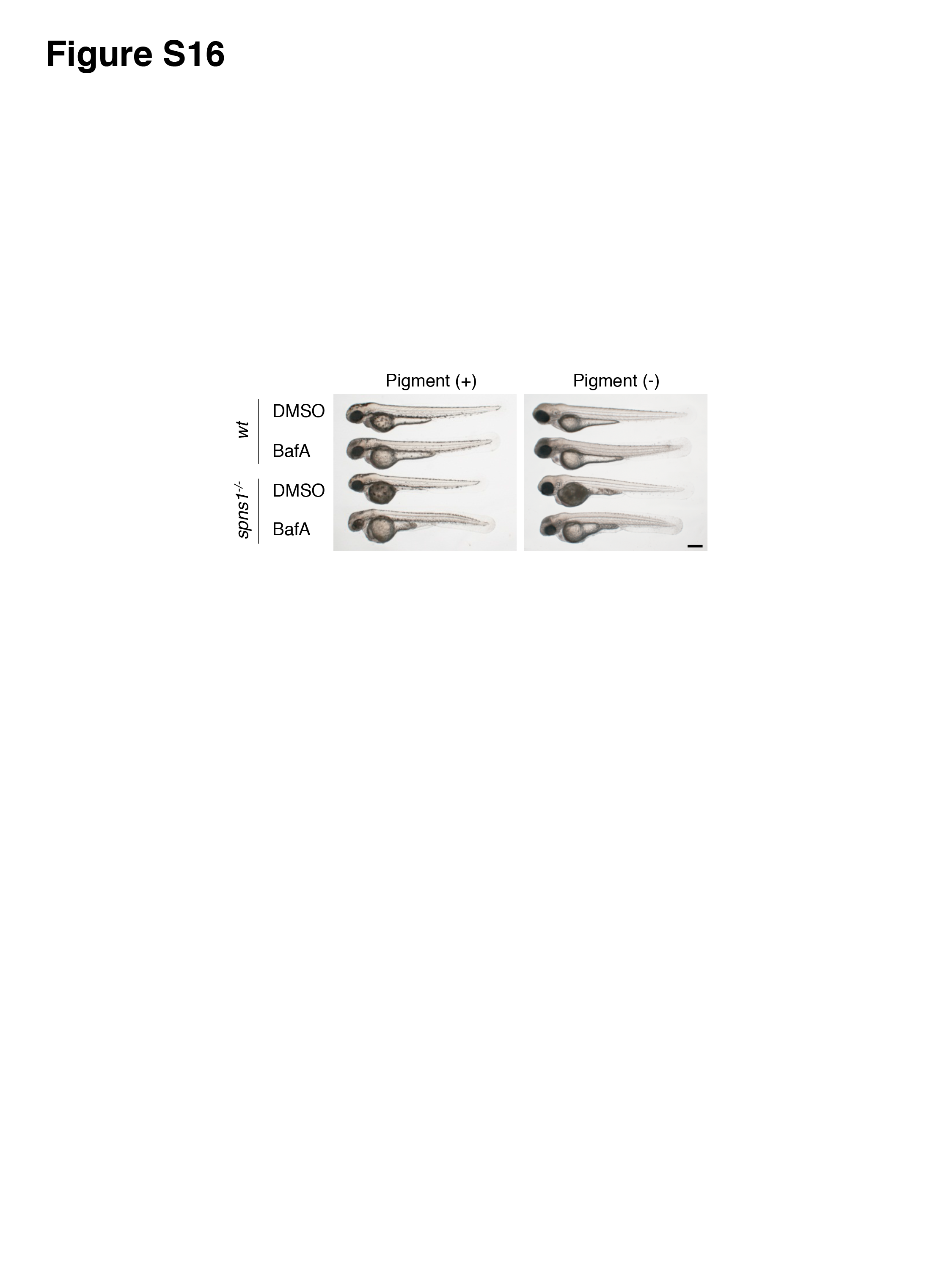

Supplement: Figure S16 — Suppression of spns1-mutant phenotypes by BafA treatment in zebrafish embryos. Suppression of yolk opacity by treatment with BafA (200 nM; 12 h treatment from 48 hpf through 60 hpf) in pigmented (AB line) and unpigmented (casper line) zebrafish embryos is shown. Scale bar, 250 µm. (TIF) [file pgen.1004409.s016.tif]

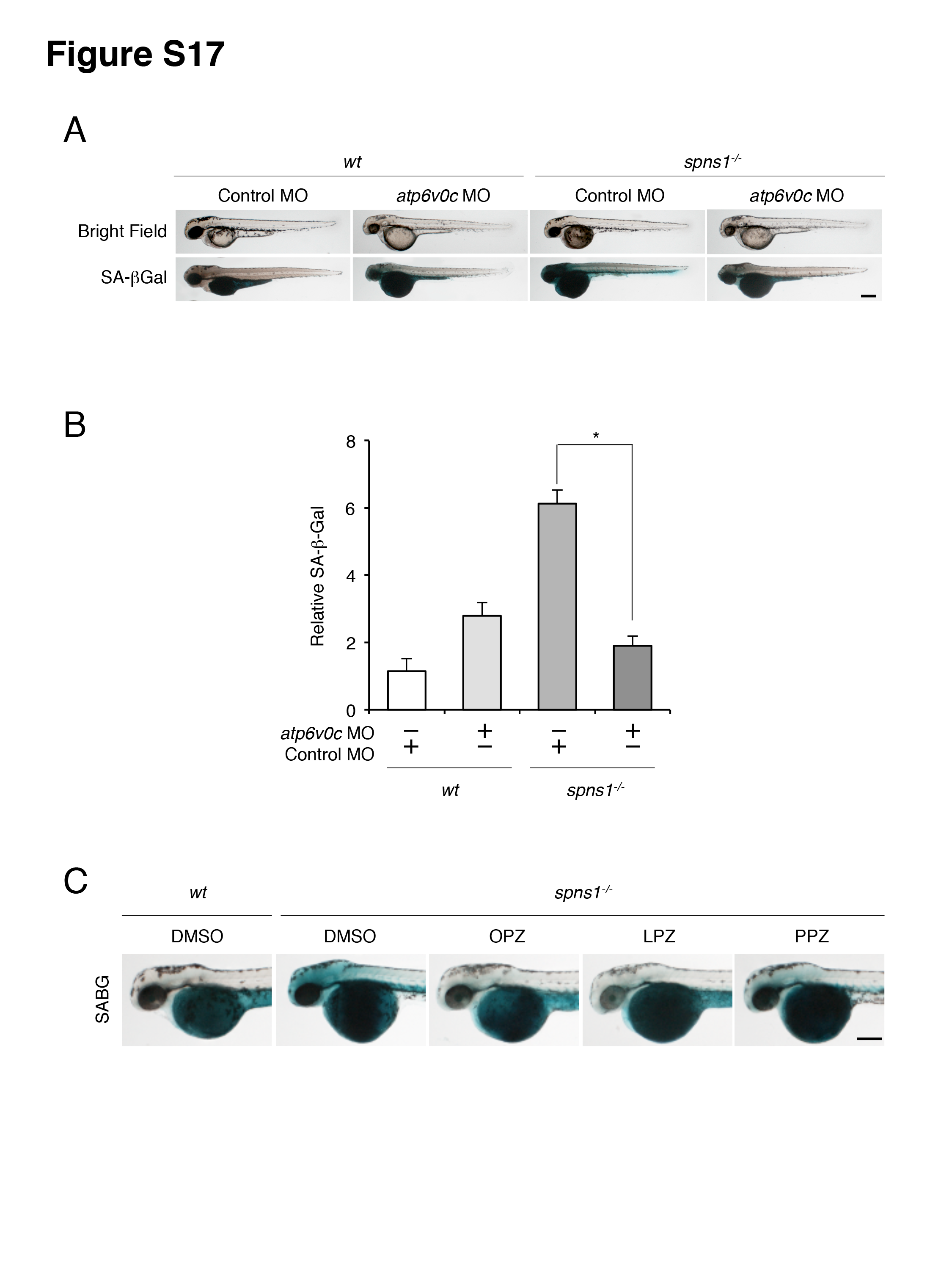

Supplement: Figure S17 — Suppression of spns1-mutant phenotypes by knockdown of the atp6v0c gene in zebrafish embryos. (A) Gross morphology, EGFP-LC3 and LysoTracker intensities in wild-type (wt) and spns1-mutant animals injected with atp6v0c MO (4 ng/embryo) at 48 hpf. Suppression of yolk opacity and SA-β-gal (SABG) by injection of atp6v0c MO in zebrafish embryos was observed at 48 and 60 hpf, respectively. Scale bar, 250 µm. (B) Quantification of the SA-β-gal intensities shown in (A). Quantification of data presented in panel A (n = 10) is shown in the right graph; the number (n) of animals is for each genotype with MO. (C) Effect of the PPIs (omeprazole; OPZ, lansoprazole; LPZ, and pantoprazole; PPZ) on embryonic senescence (SABG; SA-β-gal) in the spns1 mutant at 48 hpf. The drug treatments were done for 12 h from 36 hpf through 48 hpf. Scale bar, 250 µm. Error bars represent the mean ± S.D., *p<0.005; ns, not significant. (TIF) [file pgen.1004409.s017.tif]

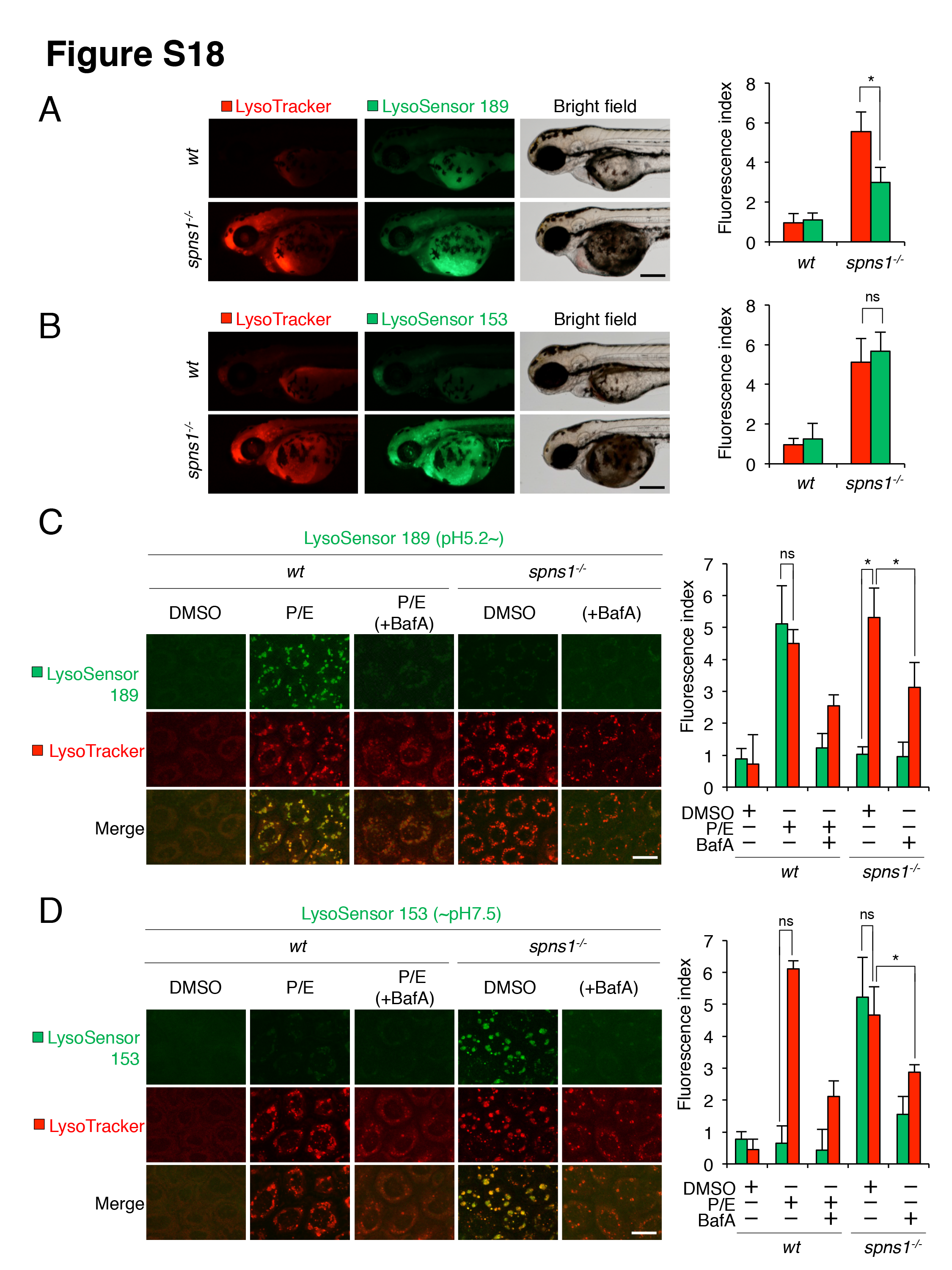

Supplement: Figure S18 — Validations of lysosomal biogenesis and acidity in zebrafish embryos. (A) Whole-mount double staining with LysoTracker (10 µM, DND-99; red) and LysoSensor 189 (1 µM, DND-189; green). Live animals at 72 hpf were counterstained with LysoTracker and acidic pH-sensitive LysoSensor 189, simultaneously. LysoSensor 189 weakly detects acidic lysosomal signals in the spns1-mutant animals. Scale bar, 250 µm. Quantification of data presented for LysoSensor 189 (green) and LysoTracker (red) signals in panel A (n = 12) is shown in the right graph; the number (n) of animals is for each genotype. (B) Whole-mount double staining with LysoTracker (10 µM, DND-99; red) and LysoSensor 153 (1 µM, DND-153; green). Animals at 72 hpf were simultaneously counterstained by LysoTracker and neutral pH-sensitive LysoSensor 153. LysoSensor 153 can detect relatively neutral lysosomal signals in the spns1-mutant animals. Scale bar, 250 µm. Quantification of data presented for LysoSensor 153 (green) and LysoTracker (red) signals in panel B (n = 12) is shown in the right graph; the number (n) of animals is for each genotype. (C) Acidic pH-sensitive LysoSensor 189 (1 µM, green) probe in combination with LysoTracker (10 µM, red) was used in wt and spns1-mutant animals, and detectable signals in cells were obtained at 72 hpf. In wt fish treated with pepstatin A and E-64-d (P/E) (5 µg/ml each for 12 h), autolysosomal and/or lysosomal compartments were more prominently detected by LysoSensor 189 at the cellular level with enhanced accumulation of enlarged compartments under the identical LysoTracker staining condition. In contrast, in spns1-mutant animals, the cellular compartments were only weakly detectable by LysoSensor 189. Importantly, the short-term BafA treatment (for 1 h) largely attenuated or abolished staining of acidic compartments by both LysoSensor and LysoTracker, indicating that these autolysosomal and lysosomal compartments in wt animals treated with pepstatin A and E-64-d may retain some [file pgen.1004409.s018.tif]

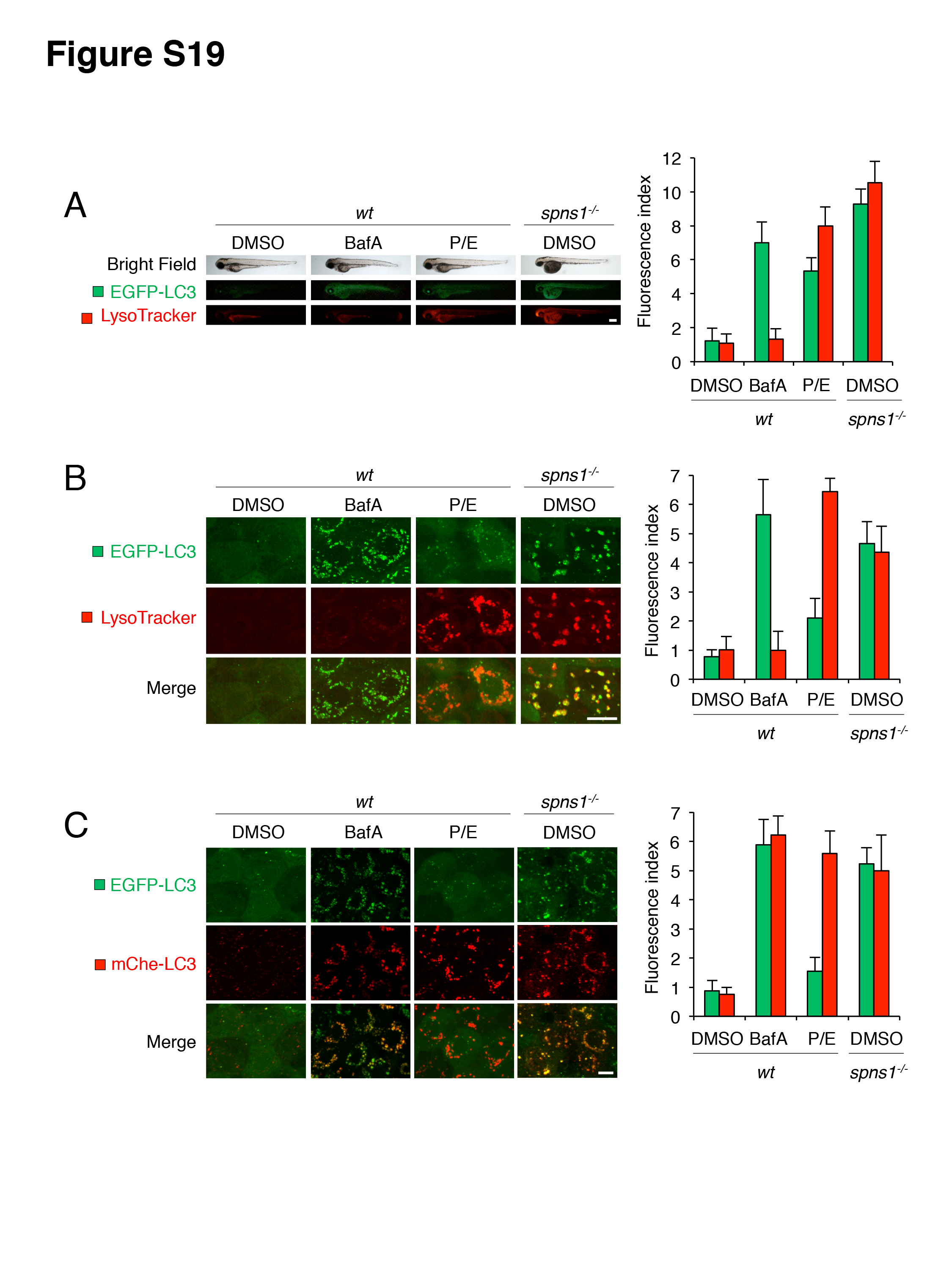

Supplement: Figure S19 — Validations of autolysosome formation and lysosomal biogenesis in zebrafish embryos. (A) Gross morphologies of BafA (100 nM)-treated or pepstatin A (5 µg/ml)- and E-64-d (5 µg/ml)-co-treated (P/E) wt [Tg(CMV:EGFP-LC3)] and spns1-mutant [Tg(CMV:EGFP-LC3); spns1hi891/hi891] animals. Embryos at 60 hpf were incubated with BafA or P/E for 12 h, and stained with LysoTracker at 72 hpf. Scale bar, 250 µm. Quantification of data presented in the middle and bottom rows (green; EGFP, red; mCherry) in panel A (n = 12) is shown; the number (n) of animals is for each genotype with DMSO, BafA or pepstatin A and E-64-d (P/E). (B) Intracellular autolysosome formation and lysosomal biogenesis in vehicle (DMSO)-treated, BafA (100 nM)-treated or pepstatin A (5 µg/ml)- and E-64-d (5 µg/ml)-treated (P/E) wt [Tg(CMV:EGFP-LC3)] and spns1-mutant [Tg(CMV:EGFP-LC3);spns1hi891/hi891] animals. Numerous large EGFP-LC3 puncta are evident in the BafA-treated embryos, with minimal LysoTracker staining. Some increased EGFP-LC3 speckles and strong enhancement of enlarged LysoTracker signals are evident in the cells from P/E-treated embryos. The same samples analyzed in (A) were observed by using confocal microscopy at a high magnification (×600). Scale bar, 10 µm. Quantification of data presented in the middle and bottom rows (green; EGFP, red; mCherry) in panel A (n = 6) is shown. The number (n) of animals is for each genotype with DMSO, BafA or pepstatin A and E-64-d (P/E). Three independent areas (periderm or basal epidermal cells above the eye) were selected from individual animals. (C) Impaired autolysosomal acidification in BafA-treated wt or in spns1-mutant embryos, but not in pepstatin A- and E-64-d-treated (P/E) wt embryos. EGFP-LC3 and mCherry-LC3 double-transgenic wt [Tg(CMV:EGFP-LC3:mCherry-LC3)] and spns1-mutant [Tg(CMV:EGFP-LC3:mCherry-LC3);spns1hi891/hi891] zebrafish were used to monitor autolysosome formation. Embryos at 60 hpf were incubated with BafA (100 nM) or P/E (5 µg/ml each) f [file pgen.1004409.s019.tif]
