# Supplementary material for: Aberrant Autolysosomal Regulation Is Linked to The Induction of Embryonic Senescence: Differential Roles of Beclin 1 and p53 in Vertebrate Spns1 Deficiency
Source: PLoS Genet. 2014 Jun 26;10(6):e1004409. doi: 10.1371/journal.pgen.1004409 (PMC4072523; doi:10.1371/journal.pgen.1004409)
Supplement: Table S1 — List of primers used for qPCR. (DOC) [file pgen.1004409.s020.doc]

**Supplemental Table 1**

| Gene | Accession No. | Target | Primers | References |
| --- | --- | --- | --- | --- |
| *p21* | XM_001923789.4 | EX(2-3)-EX3 | Forward: TGAGAACTTACTGGCAGCTTCA  Reverse: ACGTGCATTCGTCTCGTAGC | [1] |
| *pai-1* | EH445414 | EX(2-3)-EX3 | Forward: CTGATCTTTGCCCTTTGCGCATCA  Reverse: TTTGCTCAAGCTGCGCCTAAAGAC | this work |
| *smp-30* | NM_205746.1 | EX4-EX5 | Forward: ACTATGACATCCAAACTGGAGGA  Reverse: CTTCTGTGTCTATGCACATACCG | [2] |
| *bax* | AF231015 | EX(3-4)-EX4 | Forward: GCAGTGGCAATGACCAGATA  Reverse: GGAAAACTCCGACTGTCTGC | this work |
| *β−actin* | NM_181601.3 | EX5-EX6 | Forward: CCCAGACATCAGGGAGTGAT  Reverse: CACCGATCCAGACGGAGTAT | [1] |

**List of primers used for qPCR**

**References**

1. Soares J, Castro LF, Reis-Henriques MA, Monteiro NM, Santos MM (2012) Zebrafish (Danio rerio) life-cycle exposure to chronic low doses of ethinylestradiol modulates p53 gene transcription within the gonads, but not NER pathways. Ecotoxicology 21: 1513-1522.

2. Fujisawa K, Terai S, Hirose Y, Takami T, Yamamoto N, et al. (2011) Senescence marker protein 30 (SMP30)/regucalcin (RGN) expression decreases with aging, acute liver injuries and tumors in zebrafish. Biochem Biophys Res Commun 414: 331-336.
