# Supplementary material for: Aberrant Autolysosomal Regulation Is Linked to The Induction of Embryonic Senescence: Differential Roles of Beclin 1 and p53 in Vertebrate Spns1 Deficiency
Source: PLoS Genet. 2014 Jun 26;10(6):e1004409. doi: 10.1371/journal.pgen.1004409 (PMC4072523; doi:10.1371/journal.pgen.1004409)
Supplement: Table S2 — List of primers used for RT-PCR and list of PCR conditions. (DOC) [file pgen.1004409.s021.doc]

**Supplemental Table 2**

| Gene | Accession No. | Target | Primers | References |
| --- | --- | --- | --- | --- |
| *beclin 1* | NM_200872.1 | EX3-EX7 | Forward: CAAACAAGATGGCGTGGCTCGAAA  Reverse: TCCAACTCCAGCTGCTGTCTCTT | this work |
|  |  | EX4-EX7 | Forward: GTGGAACTATGGAGAACTTGAGTCGCA  Reverse: TCCAACTCCAGCTGCTGTCTCTT | this work |
| *p21* | XM_001923789.4 | EX3-EX3 | Forward: TGAGAACTTACTGGCAGCTTCA  Reverse: ACGTGCATTCGTCTCGTAGC | [1] |
| *pai-1* | EH445414 | EX2-EX3 | Forward: CTGATCTTTGCCCTTTGCGCATCA  Reverse: TTTGCTCAAGCTGCGCCTAAAGAC | this work |
| *smp-30* | NM_205746.1 | EX4-EX5 | Forward: ACTATGACATCCAAACTGGAGGA  Reverse: CTTCTGTGTCTATGCACATACCG | [2] |
| *mdm2* | AF010255 | EX1-EX10 | Forward: CAGCAAGGTTGACAACGAGA  Reverse: CGAAGGTTGTGTTGGGAGTT | [1] |
| *bax* | AF231015 | EX2-EX6 | Forward: GCAGTGGCAATGACCAGATA  Reverse: GGAAAACTCCGACTGTCTGC | [1] |
| *β−actin* | NM_131031 | EX(2-3)-EX6 | Forward: CCCAGACATCAGGGAGTGAT  Reverse: CACCGATCCAGACGGAGTAT | [1] |

**1) List of primers used for RT-PCR**

**References**

1. Liu TX, Howlett NG, Deng M, Langenau DM, Hsu K, et al. (2003) Knockdown of zebrafish Fancd2 causes developmental abnormalities via p53-dependent apoptosis. Dev Cell 5: 903-914.

2. Fujisawa K, Terai S, Hirose Y, Takami T, Yamamoto N, et al. (2011) Senescence marker protein 30 (SMP30)/regucalcin (RGN) expression decreases with aging, acute liver injuries and tumors in zebrafish. Biochem Biophys Res Commun 414: 331-336.

**2) List of PCR conditions**

| Gene | Annealing temp  (°C) | Extension time  (sec) | Cycles | Amplicon  (bp) |
| --- | --- | --- | --- | --- |
| *beclin 1* | 53 | 35 | 40 | 512 |
|  | 53 | 35 | 40 | 421 |
| *p21* | 55 | 60 | 25 | 396 |
| *pai-1* | 55 | 30 | 25 | 354 |
| *smp-30* | 51 | 15 | 25 | 98 |
| *mdm2* | 55 | 60 | 25 | 1007 |
| *bax* | 55 | 30 | 25 | 505 |
| *b-actin* | 55 | 60 | 18 | 896 |
