# Supplementary material for: Aberrant Autolysosomal Regulation Is Linked to The Induction of Embryonic Senescence: Differential Roles of Beclin 1 and p53 in Vertebrate Spns1 Deficiency
Source: PLoS Genet. 2014 Jun 26;10(6):e1004409. doi: 10.1371/journal.pgen.1004409 (PMC4072523; doi:10.1371/journal.pgen.1004409)
Supplement: Text S1 — Supplemental data. (DOC) [file pgen.1004409.s022.doc]

**Text S1**

**Supplemental Data**

***Activation of another senescence-associated lysosomal hydrolase in spns1 mutant zebrafish embryos***

While there is currently no single specific marker that can unequivocally detect senescent cells, thus far the most widely utilized method for senescence is the cytochemical detection of lysosomal β-galactosidase, as the senescence-associated β-galactosidase (SA-β-gal) [5,6], which has been used for detection of embryonic/larval senescence in ours and other studies [8,9,10,11,12,70,71]. Although the detection of SA-β-gal activity is currently the gold standard to validate cellular senescence, the mechanistic regulation and its link to increased lysosomal mass are still largely unknown, except for the observation that its hydrolase activity increases with senescence [28]. Therefore, any other hallmarks would be supportive to further clarification of the embryonic senescence phenotype in Spns1 deficiency.

Recently, another lysosomal hydrolase/glycosidase, α-L-fucosidase (α-fuc) was reported as a novel sensitive biomarker for cellular senescence [29]. Regardless of the stress stimulus and cell type, at least in mammals, α-fuc activity is induced in all canonical types of cellular senescence, including replicative, DNA damage- and oncogene-induced senescence. Thus, we also examined the utility of α-fuc as an alternative senescence marker in zebrafish. The induction of SA-α-fuc was significantly higher in *spns1* mutants than wild-type animals, showing some background staining at the head (**Fig. S3C**). The caudal venous plexus of *spns1* mutants was the most prominent region of staining (**Fig. S3C**). Whereas in most other mammalian models with senescence, the degree of SA-α-fuc upregulation was shown to be higher than that of SA-β-gal [29], in zebrafish, the detection of SA-β-gal was still found to be more sensitive (**Fig. S3C**).

***Undetectable apoptotic cell death in spns1-depleted zebrafish embryos***

To detect cell death by a conventional method in zebrafish, we first performed acridine orange (AO) staining [32,33]. AO is cell-permeable and interacts with DNA and RNA by intercalation or electrostatic attractions, which allow us to readily identify engulfed apoptotic cells, because it will fluoresce upon engulfment. However, AO can also enter acidic compartments such as lysosomes and become protonated and sequestered [30,31]. Therefore, AO is a versatile but non-specific dye to detect apoptotic cell death. In fact, in *spns1* mutant fish, AO-stained enlarged lysosomal compartments, identified by co-staining with LysoTracker. This AO-staining was detectable in cells with intact nuclei, as confirmed by Hoechst 33342 staining (**Fig. S4**). The induction of AO- and LysoTracker-costained compartments were eliminated following coinjections of *beclin 1* MO into *spns1* morphants, while *beclin 1* MO itself did not induce any particular impact on either staining pattern (**Fig. S5A**). Therefore, to distinguish apoptotic cell death specifically, we performed a TUNEL assay in the *spns1*-deficient animals and found that apoptosis was negligible (**Fig. S5B**). Since developmentally required apoptosis is reduced in *Drosophila* *spinster* mutants [13], our observation in *spns1*-deficient zebrafish embryos is consistent with this evidence.

***UV-mediated DNA damage leading to apoptosis induction in spns1-deficient zebrafish embryos***

We utilized ultraviolet (UV)-mediated DNA damage to induce apoptosis in zebrafish embryos [34]. UV-irradiated zebrafish embryos obviously showed fragmented nuclei stained by Hoechst 33342 (**Fig. S4B)**. These Hoechst-positive fragmented nuclei were also occasionally colocalized with the compartments stained by AO as well as LysoTracker (**Fig. S4B and Fig. S5A)**, suggesting that lysosomal compartments potentially engulfed apoptotic nuclei. To observe apoptotic cell death more specifically, *spns1*-deficient animals were also exposed to UV irradiation followed by TUNEL assays. We found an increased TUNEL-positive signal in both *spns1*-defective fish and wild-type fish (**Fig. S9A**).

***Activated p53-dependent autophagic phenotypes leading to apoptosis and senescence upon UV-mediated DNA damage***

Next, we further examined if and how UV exposure could affect the autophagic progression in zebrafish embryos, and found that the aggregations of GFP-LC3 were increased after irradiation. This enhanced autophagy by UV treatment was only observed in the presence of p53, suggesting that activated p53 exacerbates the *spns1*-defective state through the induction of both autophagy and apoptosis (**Fig. S9**). Thus, under the *spns1*-defective condition, while basal p53 functions as a suppressor of *spns1* defect-mediated senescence by preventing autophagy, activated p53 can be a canonical inducer of both apoptosis and senescence, followed by promotion of autophagy (**Fig. S9 and Fig. S12**).

***The effects of DNA damage response and p53 on cell proliferation in spns1-defective zebrafish embryos***

Many senescence-inducing stimuli generate a sustained DNA damage response (DDR) that can be visualized by persistent nuclear DNA damage foci, termed “DNA segments with chromatin alterations reinforcing senescence” (DNA-SCARS) and the accumulation of DDR proteins, including ATM, Chk2/CHEK2, p53-binding protein-1/TP53BP1 and the histone variant γH2AX [40]. Therefore, we adopted the well-established γH2AX (phospho-histone H2AX) detection in zebrafish [32]. As indicated by an increased abundance of γH2AX, the elevated TUNEL-positive intensities consistently correlated with increased levels of DNA damage in the UV-irradiated *spns1*-deficient fish as well as wild type, but not in either of the non-irradiated animals (**Fig. S10**). Thus, our data indicate that the *spns1* deficiency itself may not significantly affect the accumulation of DNA damage, and irrespective of the *spns1* state, both DDR and apoptotic induction can still occur.

Given the obvious appearance of senescent cells in Spns1 deficiency, we assessed whether cell proliferation was altered. Being consistent with DDR by UV treatment, synergistic suppression of 5-bromo-2-deoxyuridine (BrdU) incorporation was prominently detected in either wild-type or *spns1*-deficient fish in a p53-dependent manner. Conversely, under the untreated basal condition, the incorporation of BrdU in *spns1*-deficient animals was slightly but significantly reduced in a p53-independent manner (**Fig. S10**). Since DDR was not prominent in *spns1*-deficient embryos unstimulated by UV, this result suggests that progression through the S phase (DNA synthesis) during the cell cycle is decreased due to the Spns1 defect itself (**Fig. S10**). In addition, the immunodetection of a mitotic marker, phosphorylated histone H3 (pH3), revealed a significant reduction in *tp53^+/+^*-*spns1* mutant animals even in the absence of UV irradiation. There was also a similar tendency of such pH3 reduction in non-irradiated *spns1;tp53*-double mutants, but it was not statistically significant (**Fig. S11**). Embryonic SA-β-gal activity was consistently increased by the UV stimulation in both wild-type and *spns1* mutant animals in the presence of p53 (**Fig. S12**).

***The impact of senescence-associated gene expression in spns1-defective zebrafish embryos***

Next, to demonstrate senescence-associated gene expression in *spns1*-defective zebrafish embryos, semi-quantitative RT-PCR was used to assay individual embryos for the gene expression of *p21^waf1/cip1^*, *plasminogen activator inhibitor-1* (*pai-1*) *bax*, and *mdm2*, which are downstream targets of the p53 pathway*,* and *senescence marker protein-30* (*smp-30*), whose expression decreases with age in rodents and zebrafish [42,43], accompanied by *β-actin*, which was used as a normalization control.

A significant upregulation of all four p53-target genes (i.e., *p21^waf1/cip^*, *pai-1*, *bax*, and *mdm2*) was observed in both *spns1* mutants and morphants, when compared with wild-type and control MO-injected embryos, respectively (**Fig. S13 and S14**). Moreover, *smp-30* was downregulated in *spns1*-deficient animals compared with the corresponding controls. By contrast, solely injected *beclin 1* morphants did not show significant changes in the expression of these genes (**Fig. S14A**). Importantly, however, suppression of *beclin 1* significantly counteracted the impact of the *spns1* depletion on the expression of the *pai-1* *smp-30*, and *mdm2* genes by restoring the levels substantially, but not for *p21^waf1/cip^* and *bax* (**Fig. S14A**). The induction of the *p21^waf1/cip1^*, *bax*, and *mdm2* genes in the *spns1*-defective conditions (both *spns1* morphants and mutants) was p53-dependent, as confirmed by the levels of the expression of these genes in *p53* mutants (**Fig. S14B**).

Intriguingly, irrespective of the p53 state and/or UV treatment, the *pai-1* expression levels were much higher in *spns1*-defective animals, though it has been reported that *pai-1* is a critical downstream target of p53 for senescence induction [39]. We could still detect a significant increase of the *pai-1* expression in *spns1* morphants and mutants, under the p53-depleted/defective conditions (**Fig. S14B**). The expression of *smp-30* was decreased irrespective of the p53 status in *spns1*-defective fish embryos, which is consistent with a characteristic of accelerated senescence in animals [44,45]. While no obvious p53-dependent alteration of the *smp-30* expression was observed in wild-type (*p53^+/+^/spns1^+/+^*) fish upon UV treatment, in *spns1* mutant (*p53^+/+^/spns1^-/-^*) fish a minor but significant reduction was observed. In the absence of p53, however, the *smp-30* expression level was already reduced in *spns1* mutant fish and its further reduction was not detected in UV-treated animals (**Fig. S14B**). Taken together, both upregulation of *pai-1* and downregulation of *smp-30* in *spns1*-defective fish embryos are symptomatically similar to the induction of senescence characteristics in aging organisms [42]. In addition, since even in the absence of p53, the up- and downregulation of these two critical senescence markers, *pai-1* and *smp-30*, respectively, were still detectable in *spns1*-deficit animals, it seems likely that p53-independent regulation may contribute to the expression of these genes.
