# Supplementary material for: Aberrant Autolysosomal Regulation Is Linked to The Induction of Embryonic Senescence: Differential Roles of Beclin 1 and p53 in Vertebrate Spns1 Deficiency
Source: PLoS Genet. 2014 Jun 26;10(6):e1004409. doi: 10.1371/journal.pgen.1004409 (PMC4072523; doi:10.1371/journal.pgen.1004409)
Supplement: Text S2 — Supplemental Materials and Methods. (DOC) [file pgen.1004409.s023.doc]

**Text S2**

**Supplemental Materials and Methods**

***Whole mount staining for senescence-associated lysosomal α-L-fucosidase (SA-α-fuc)***

The SA-α-fuc assay was basically preformed as described by Hildebrand et al. [29]. After fixation in 4% paraformaldehyde (PFA)/phosphate buffered saline (PBS) and washing in PBS (pH 5.0), samples were incubated in McIlvain phosphate-citrate buffer (pH 5.0) containing 3 mM potassium ferrocyanide and 3 mM potassium ferricyanide, using 4 mM 5-bromo-4-chloro-3-indolyl-α-L-fucopyranoside (X-fuc) (Carbosynth US LLC, CA) as a substrate, at 37°C for 24-48 h.

***Acridine orange staining of zebrafish embryos***

Live zebrafish embryos were dechorionated in pronase (2.0 mg/ml, in E3 medium; 5 mM NaCl, 0.17 mM KCl, 0.33 mM CaCl_2_, 0.33 mM MgSO_4_) for 3 to 5 min and rinsed five times in E3 medium. Embryos were then incubated in 10 µg/ml acridine orange (AO) (Sigma A-6014) in E3 medium for 1 h at 28.5°C, followed by 3 quick rinses. Subjected embryos were anesthetized in 160 µg/ml tricaine (3-aminobenzoic acid ethyl ester) (A-5040, Sigma), and mounted laterally on a glass slide, as described previously [33].

***Quantitative and semi-quantitative RT-PCR analysis***

Total RNA was extracted from tissues or pools of three embryos using TRIzol (Invitrogen, CA). SuperScript III reverse transcriptase (Invitrogen, CA) was used to synthesize cDNA from 1 μg of total RNA. Quantitative RT-PCR (q-PCR) was carried out with a StepOnePlus Real-Time PCR System (Applied BioSystems, Foster City, CA) using 2x SYBR-Green PCR Master Mix (Applied Biosystems, Foster City, CA). Total RNA from each sample was normalized to *β-actin*. In each qPCR experiment, pools of three embryos were run in quadruplicate. Primer pairs for qPCR are listed in **Supplemental Table 1**. In each semi-qPCR, RT-PCR products were electrophoresed on an agarose gel and visualized with ethidium bromide staining. The intensity of the bands was measured using the ImageJ software (National Institutes of Health, Bethesda, MD) and the normalization was performed using the amount of *β-actin* in each condition. Each experiment was performed at least four times and representative data of gel bands are shown. Primer pairs, parameters and conditions for semi-qPCR were summarized in **Supplemental Table 2**.

***Detection for apoptosis, γH2AX, pH3, and BrdU labeling***

For the TUNEL assay, embryos grown at particular developmental stages were fixed overnight in 4% PFA with PBS at 4°C and stored in 100% methanol at -20°C until the samples were used. Samples were then incubated in 100% acetone at -20°C for 20 min. Following fixation, the embryos were rinsed three times with PBS containing 0.1% Tween-20 (PBST; 5 min each). Samples were then permeabilized by treatment with 0.5% Triton X-100 and 0.1% sodium citrate in PBS for 15 min, then with 5 to 50 mg/ml proteinase K (Invitrogen) for 5 to 25 min depending on embryo stages. Embryos were subjected to the TUNEL assay by using the ApopTag Red *in situ* Apoptosis Detection Kit (EMD Millipore, Billerica, MA) according to the manufacturer’s instructions. For whole-mount immunostaining with γH2AX or pH3 antibody, embryos were fixed overnight in 4% PFA with PBS at 4°C, incubated in 100% methanol at -20°C, and then treated with 100% acetone at -20°C for 7 min. After fixation, embryos were rinsed in 50% PBS/50% methanol at -20°C for 1 h, rinsed once in water and then twice in PBST (5 min each). Embryos were blocked for 1 h in blocking solution [PBS containing 1% BSA, 2% normal goat serum, 0.1% Tween-20, 1% dimethyl sulfoxide (DMSO)] and then incubated overnight at 4°C with a polyclonal anti-pH3 (Ser10) antibody (Upstate-Millipore, MA) (1:200) or a polyclonal anti-γH2AX (Ser139) antibody (GTX127342, GeneTex, CA) (1:200). Finally, the embryos were washed 4 times in PBS-DT (PBS including, 0.1% Tween-20 and 1% DMSO) for 15 min, and incubated in TRITC-conjugated anti-rabbit secondary antibody (Southern Biotechnology, AL) (1:300) in blocking solution for 4 h at room temperature. In the 5-bromo-2’-deoxyuridine (BrdU) incorporation assay, BrdU (Sigma-Aldrich, MO) with a final concentration of 10 mM was dissolved in E3 medium containing 15% dimethyl sulfoxide (DMSO). Embryos at 47 or 71 hpf were manually dechorionated and soaked in the 10 mM BrdU solution with 1% DMSO in E3 medium at 28.5°C. Samples were fixed for 2 h in 4% PFA with PBS at room temperature, rinsed twice in PBS, incubated in 2 N HCl for 30 min at room temperature, and transferred in 0.1 M Tris-HCl (pH 7.5) for 5 min, blocked in FCS-PBST (10% fetal calf serum, 1% DMSO, in PBST) solution for 30 min, and incubated with anti-BrdU Cy3-labeled antibody (BD-Biosciences-Pharmingen, CA) (1:100). In some experiments, stained embryos were counterstained with 4’,6-diamidino-2-phenylindole (DAPI) for 5 min, rinsed in PBS twice, flat mounted, and processed to generate microscopy images.

***UV irradiation to zebrafish embryos***

Dechorionated embryos placed in 6-well culture plates were exposed to UV light by UV Crosslinker (Stratalinker 2400, Stratagene, CA) with 0.18 mj/cm^2^.

***Quantitative analysis and statistics***

Data processing and statistical analyses were performed using Statistical Package for the Social Sciences (SPSS) version 14.0. This software was used to generate each of the scatter plots, tables and graphs shown in the text and perform statistical tests where appropriate. Additional statistical analyses were performed using a custom MATLAB script to analyze survival estimates by the Kaplan and Meier method, and to compare survival between mutant fish and their wild-type controls using the log-rank test. The Gompertz-Makeham model for age-specific mortality M(x)=A_0_⋅exp(G⋅x)+M_0_, with initial mortality A_0_, rate of aging G, age x, and age-independent mortality M_0_ can be shown to give the survival proportion S(x)=exp[(A_0_/G)(1-exp(G⋅x) - M_0_⋅x]. Non-linear-least-squares fits to S(x) was performed using a Trust-Region Reflective Newton algorithm implemented by the MATLAB *fit()* function (The MathWorks, Natick, MA). Maximum lifespan was estimated as the age of 1% survival.
